# Smad7 effects on TGF-**β** and ErbB2 restrain myofibroblast activation and protect from postinfarction heart failure

**DOI:** 10.1172/JCI146926

**Published:** 2022-02-01

**Authors:** Claudio Humeres, Arti V. Shinde, Anis Hanna, Linda Alex, Silvia C. Hernández, Ruoshui Li, Bijun Chen, Simon J. Conway, Nikolaos G. Frangogiannis

**Affiliations:** 1 The Wilf Family Cardiovascular Research Institute, Department of Medicine (Cardiology), Albert Einstein College of Medicine, Bronx, New York, USA.; 2 Herman B. Wells Center for Pediatric Research, Indiana University School of Medicine, Indianapolis, Indiana, USA.

**Keywords:** Cardiology, Immunology, Fibrosis, Growth factors, Heart failure

## Abstract

Repair of the infarcted heart requires TGF-β/Smad3 signaling in cardiac myofibroblasts. However, TGF-β–driven myofibroblast activation needs to be tightly regulated in order to prevent excessive fibrosis and adverse remodeling that may precipitate heart failure. We hypothesized that induction of the inhibitory Smad, Smad7, may restrain infarct myofibroblast activation, and we examined the molecular mechanisms of Smad7 actions. In a mouse model of nonreperfused infarction, Smad3 activation triggered Smad7 synthesis in α-SMA^+^ infarct myofibroblasts, but not in α-SMA^–^PDGFRα^+^ fibroblasts. Myofibroblast-specific Smad7 loss increased heart failure–related mortality, worsened dysfunction, and accentuated fibrosis in the infarct border zone and in the papillary muscles. Smad7 attenuated myofibroblast activation and reduced synthesis of structural and matricellular extracellular matrix proteins. Smad7 effects on TGF-β cascades involved deactivation of Smad2/3 and non-Smad pathways, without any effects on TGF-β receptor activity. Unbiased transcriptomic and proteomic analysis identified receptor tyrosine kinase signaling as a major target of Smad7. Smad7 interacted with ErbB2 in a TGF-β–independent manner and restrained ErbB1/ErbB2 activation, suppressing fibroblast expression of fibrogenic proteases, integrins, and CD44. Smad7 induction in myofibroblasts serves as an endogenous TGF-β–induced negative feedback mechanism that inhibits postinfarction fibrosis by restraining Smad-dependent and Smad-independent TGF-β responses, and by suppressing TGF-β–independent fibrogenic actions of ErbB2.

## Graphical abstract

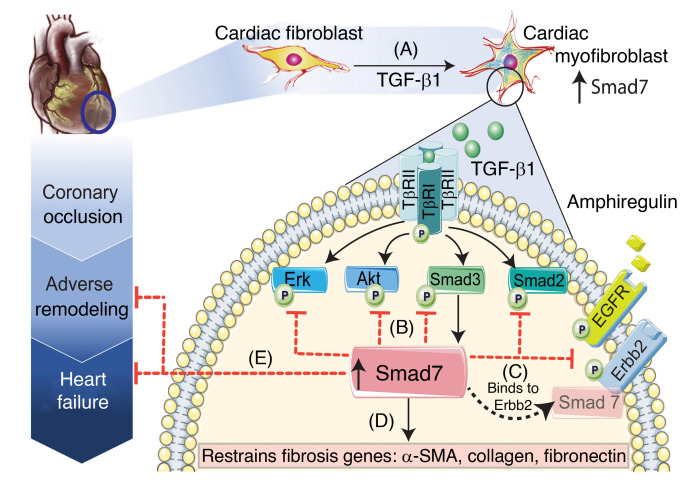

## Introduction

The adult mammalian myocardium contains abundant fibroblasts (1, 2). Following myocardial infarction (MI), fibroblasts become activated and play a critical role in repair, protecting the heart from catastrophic rupture (3, 4). However, the cardiac reparative response requires tight regulation of fibroblast activation. Early expansion of matrix-synthesizing myofibroblasts during the proliferative phase of infarct healing is followed by deactivation and acquisition of a quiescent fibroblast phenotype in the mature scar (5). Considering the importance of timely stimulation and suppression of fibroblast activity in the healing heart, impaired myofibroblast deactivation following MI would be expected to cause progressive fibrosis and may contribute to the pathogenesis of post-MI heart failure. Thus, endogenous negative regulators of fibrogenic pathways may play a crucial role in protection of the infarcted heart from adverse remodeling. The molecular signals responsible for suppression of the cardiac fibrotic response following injury remain poorly understood.

The members of the transforming growth factor β (TGF-β) family are central mediators of fibroblast activation (6, 7). TGF-βs signal by binding to a heteromeric receptor complex composed of the constitutively active type II TGF-β receptor (TβRII) and a type I receptor (8). Phosphorylation of the type I receptor upon ligand binding recruits and activates intracellular effectors, the receptor-activated Smads (R-Smads), or transduces signals through Smad-independent pathways (9). In fibroblasts, TGF-β stimulation triggers myofibroblast conversion (10), induces synthesis of collagens and fibronectin, and activates a matrix-preserving program that is associated with inhibition of protease activity and upregulation of antiproteases, such as tissue inhibitors of metalloproteinases (TIMPs) (11) and plasminogen activator inhibitor 1 (PAI-1) (12). Moreover, TGF-βs stimulate cardiac fibroblast migration and induce integrin expression on the cell surface. In cardiac fibroblasts, TGF-βs exert their activating effects predominantly through the Smad3 cascade (4, 11, 13, 14) or via non-Smad pathways, such as p38 MAPK (15).

Although activation of TGF-β/Smad3 in fibroblasts is critical for repair of the infarcted heart, contributing to formation of organized myofibroblast arrays and protecting from ventricular rupture and adverse dilative remodeling (4), fibrogenic TGF-β signaling needs to be tightly regulated in order to prevent uncontrolled fibrogenic activation. The inhibitory Smads, Smad6 and Smad7, lack the C-terminal SSXS motif and cannot be phosphorylated upon binding to type I receptors, but serve as endogenous inhibitors of signals transduced by TGF-β superfamily ligands through interactions with TβRs or R-Smads (16). Smad6 preferentially inhibits bone morphogenetic protein (BMP) responses, mediated through ALK3 and ALK6, whereas Smad7 inhibits both TGF-β– and BMP-induced cascades (17). The potential role of Smad7 as a regulator of cardiac fibrosis has not been studied.

We hypothesized that Smad7 may be induced in infarct fibroblasts, thus limiting responsiveness to TGF-βs and restraining fibrosis. We found that following infarction, Smad7 is induced in myofibroblasts (and not in α-smooth muscle actin^–^ [α-SMA^–^]/PDGFRα^+^ fibroblasts) in a Smad3-dependent manner. Cell-specific loss-of-function approaches showed that myofibroblast Smad7 protects the infarcted heart from heart failure–related mortality and from adverse remodeling, limiting fibrosis in the infarct border zone and in the papillary muscles. The effects of Smad7 on the TGF-β cascade are mediated through actions downstream of the TβRs, involving inhibition of both Smad2/3-mediated and Smad-independent TGF-β–stimulated pathways. Surprisingly, unbiased transcriptomic and proteomic studies and pharmacologic inhibition experiments demonstrated that the antifibrotic effects of fibroblast Smad7 involve inhibition of epidermal growth factor receptor (EGFR) signaling through a TGF-β–independent interaction with ErbB2. Thus, Smad7 acts not only as a TGF-β inhibitor, but exerts its potent antifibrotic actions, at least in part, by interfering with the EGFR/ErbB system.

## Results

### TGF-βs induce Smad7 synthesis in cultured cardiac fibroblasts.

Mouse ventricular fibroblasts cultured in collagen lattices were stimulated with TGF-β superfamily members. All 3 TGF-β isoforms, but not activins, myostatin, or GDF11, induced *Smad7* synthesis in cardiac fibroblasts (Supplemental Figure 1A; supplemental material available online with this article; https://doi.org/10.1172/JCI146926DS1).

### Smad7 expression is markedly upregulated in infarct fibroblasts, is associated with fibroblast to myofibroblast conversion, and is dependent on Smad3 signaling.

**Figure 1:**
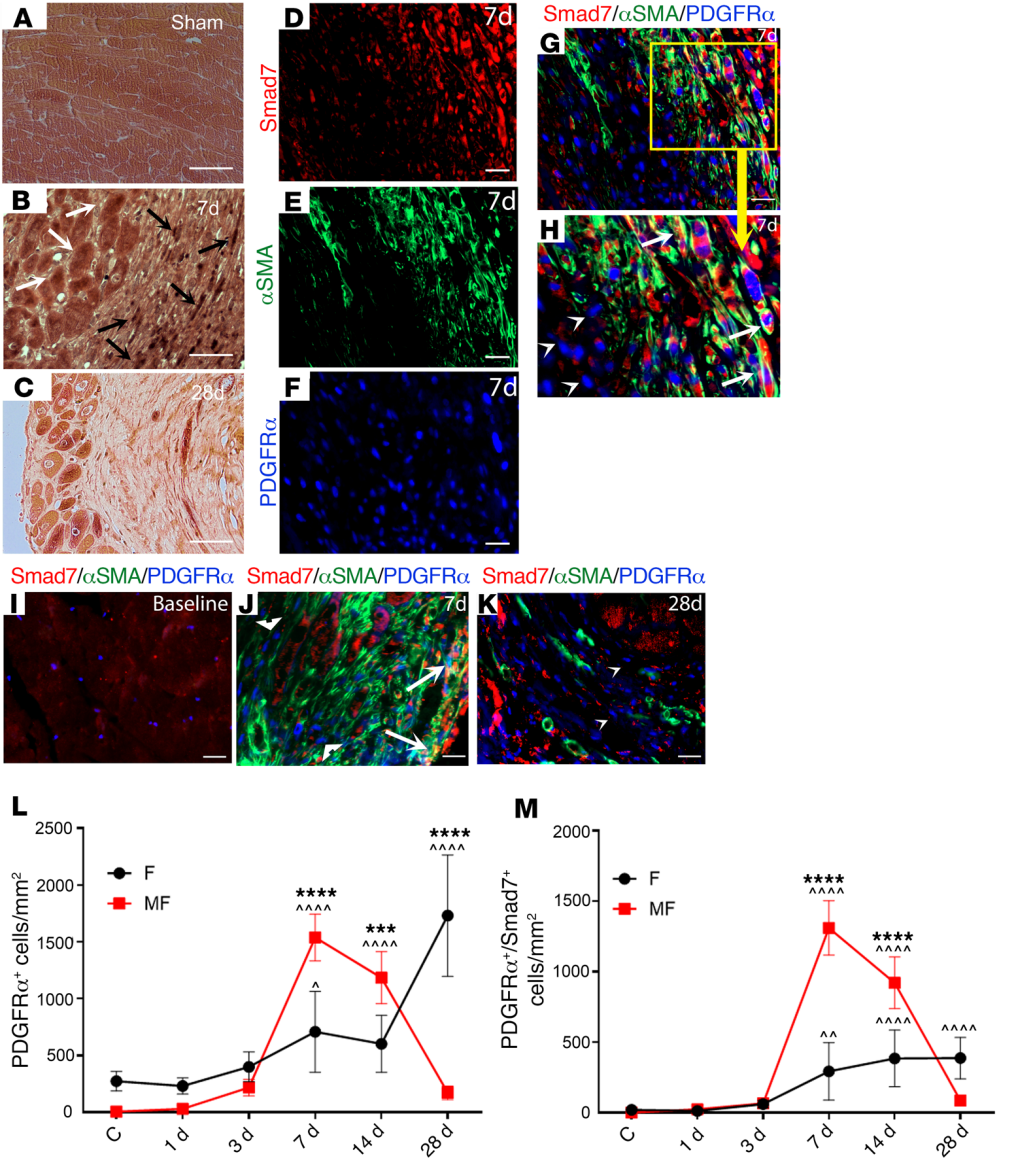
Smad7 expression is markedly upregulated in infarcted fibroblasts and is associated with fibroblast-to-myofibroblast conversion. (**A**) Smad7 immunohistochemical staining shows low-level Smad7 immunoreactivity in sham mouse hearts. (**B** and **C**) Myocardial infarction is associated with a marked increase in Smad7 expression levels, peaking after 7 days of coronary occlusion. Smad7 immunoreactivity is localized in both interstitial cells (black arrows) and in border zone cardiomyocytes (white arrows). Smad7 immunoreactivity is reduced 28 days after coronary occlusion. (**D**–**K**) In PDGFRα-GFP fibroblast reporter mice, triple immunofluorescent staining of Smad7, α-SMA, and PDGFRα-GFP was used to identify infarct fibroblasts (PDGFRα^+^α-SMA^–^) and myofibroblasts (PDGFRα^+^α-SMA^+^) expressing Smad7. Individual (**D**–**F**) and merged (**G** and **H**) channels for Smad7, α-SMA, and PDGFRα-GFP fluorescence show that Smad7 is predominantly expressed by α-SMA^+^ myofibroblasts (arrows) and not by α-SMA^–^ fibroblasts (arrowheads). The time course of Smad7 expression shows that (**I**) in sham hearts, myofibroblasts are absent and Smad7 levels are low. (**J**) Seven days after infarction, abundant myofibroblasts express high levels of cytosolic Smad7 (arrows), whereas fibroblasts have negligible Smad7 immunoreactivity (arrowheads). (**K**) Relatively few α-SMA–expressing myofibroblasts are noted 28 days after infarction. Abundant fibroblasts are present; the majority of these cells are Smad7^–^ (arrow). (**L** and **M**) Quantitative analysis shows that at the 7- and 14-day time points, abundant myofibroblasts express Smad7, whereas in the mature scar (28 days after infarction), Smad7 is expressed by a fraction of fibroblasts. Statistical comparison (**L** and **M**) was performed using 1-way ANOVA followed by Tukey’s multiple comparison test (*n =* 6/group). ^^^*P <* 0.05, ^^^^*P <* 0.01, ^^^^^^*P <* 0.0001 vs. control (C); ***P < 0.001, ****P < 0.0001 between fibroblasts (F) and myofibroblasts (MF) at the same time point. Scale bars: 50 μm (**A**–**C**) and 20 μm (**D**–**K**).

Immunohistochemical staining showed low-level Smad7 immunoreactivity in sham mouse hearts (Figure 1A). MI was associated with a marked increase in Smad7 expression levels, peaking after 7 days of permanent coronary occlusion. Smad7 immunoreactivity was localized in both interstitial cells and in border zone cardiomyocytes (Figure 1, B and C). In order to examine whether Smad7 is expressed in infarct fibroblasts and myofibroblasts, we performed Smad7/α-SMA immunofluorescent staining in infarcted PDGFRα^GFP^ fibroblast reporter mice. In this model, infarct myofibroblasts are identified as α-SMA^+^PDGFRα^+^ cells, whereas fibroblasts that do not undergo myofibroblast conversion are α-SMA^–^PDGFRα^+^. Triple immunofluorescence for Smad7, α-SMA, and PDGFRα showed that the number of Smad7^+^ fibroblasts peaked 7 days after MI (Figure 1, D–M), and that the majority of these cells were myofibroblasts (Figure 1, D–K, and Supplemental Figure 1H). Moreover, PDGFRα^+^α-SMA^–^ infarct fibroblasts at the 1-day and 3-day time points (prior to myofibroblast conversion) had negligible Smad7 expression. PDGFRα staining identified a large population of fibroblasts in the mature scar, after 28 days of coronary occlusion (Figure 1, K–L). A relatively small fraction of these cells expressed Smad7 (Figure 1M and Supplemental Figure 1H). Localization of Smad7 in myofibroblasts was confirmed by performing Smad7 staining in *Postn*-Cre;ROSA26^EYFP^ mice, in which activated myofibroblasts are labeled (Supplemental Figure 1, I–O). Periostin^+^ infarct myofibroblasts infiltrating the infarcted myocardium 7 days after coronary occlusion exhibited intense Smad7 immunoreactivity. In vitro, we compared *Smad7* expression levels between cardiac fibroblasts cultured in plates (which exhibit a myofibroblast phenotype; ref. 18) and fibroblasts cultured in collagen pads (which have low levels of α-SMA expression; ref. 18). Myofibroblasts in culture plates had much higher *Smad7* expression than fibroblasts populating collagen lattices (Supplemental Figure 1B).

TGF-β signals through Smad-dependent and non-Smad pathways. In order to examine the mechanism of TGF-β–induced Smad7 induction in cardiac fibroblasts, we studied the effects of Smad3 loss on fibroblast Smad7 expression. Cardiac fibroblasts harvested from *Smad3*-KO mice (13) had markedly attenuated *Smad7* expression upon stimulation with TGF-β1 (Supplemental Figure 1C). Moreover, infarct fibroblasts harvested from mice with myofibroblast-specific Smad3 loss (4) had significantly lower *Smad7* levels in comparison to cells harvested from *Smad3^fl/fl^* infarcts (Supplemental Figure 1D). These findings suggest that Smad7 induction is predominantly found in infarct myofibroblasts and is mediated through activation of TGF-β/Smad3 signaling.

### Mice with myofibroblast-specific loss of Smad7 have no baseline defects.

In order to investigate the role of Smad7 in regulation of the fibroblast phenotype, we generated mice with myofibroblast-specific Smad7 loss (MFS7KO, Supplemental Figure 2A). Dual immunofluorescence of mouse hearts after 7 days of permanent occlusion demonstrated myofibroblast-restricted Smad7 loss; in contrast, other cell types (such as border zone cardiomyocytes) exhibited Smad7 expression (Supplemental Figure 2, B–E). MFS7KO mice appeared normal and had no baseline defects. In the absence of injury, fibroblasts harvested from MFS7KO and *Smad7^fl/fl^* mice had comparable expression of matrix genes, reflecting the absence of recombination in control cardiac fibroblasts (Supplemental Figure 2F). Body weight, heart rate, and echocardiographic parameters reflecting systolic and diastolic function were comparable between MFS7KO and *Smad7^fl/fl^* animals in both male and female groups (Supplemental Figure 3).

### Myofibroblast-specific Smad7 loss increases heart failure–related mortality in infarcted mice.

**Figure 2:**
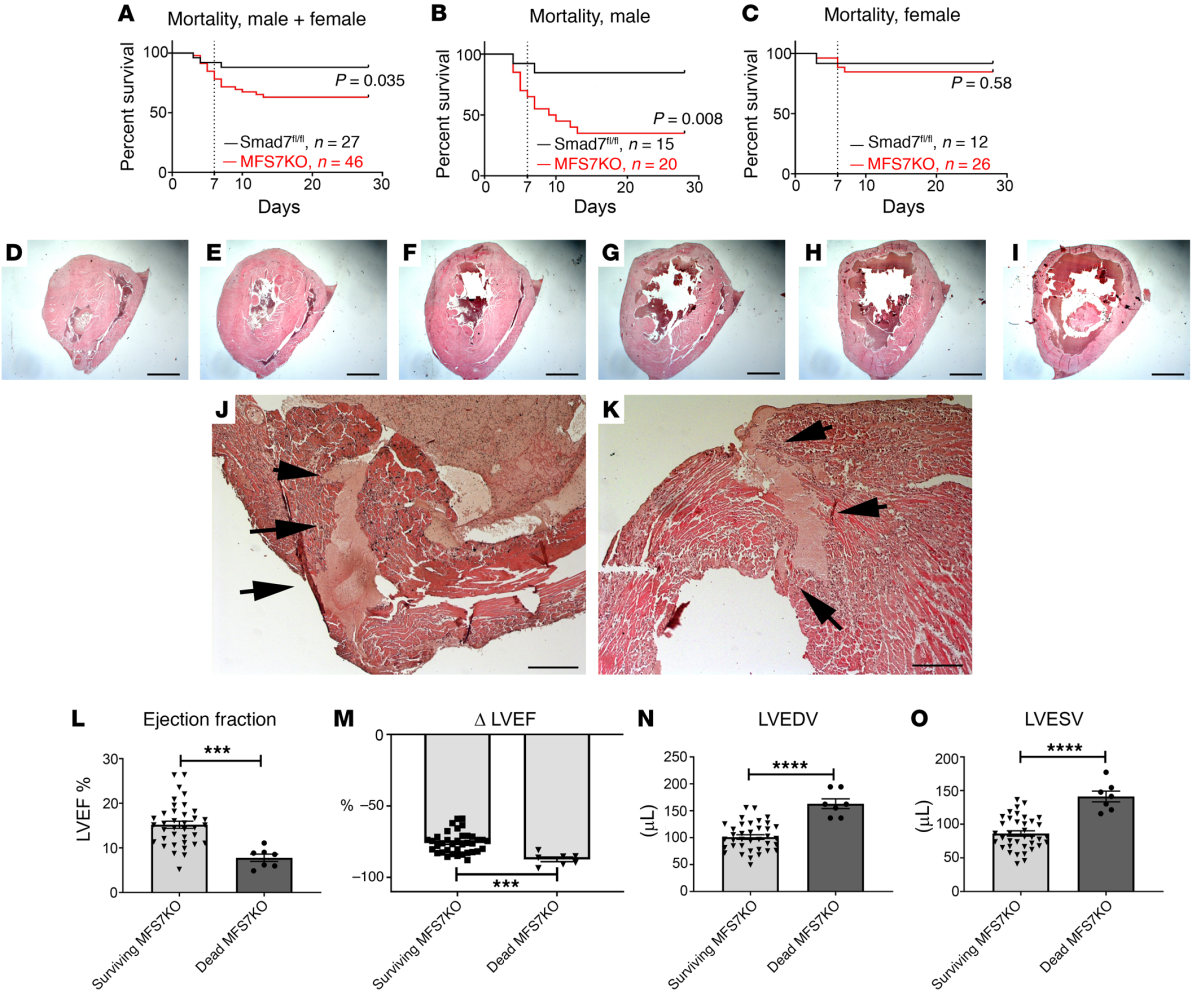
Myofibroblast-specific Smad7 loss increases heart failure–related mortality in infarcted mice. (**A**–**C**) Comparison of survival curves between *Smad7^fl/fl^* and myofibroblast-specific *Smad7*-knockout (MFS7KO) mice after 28 days of permanent coronary occlusion. (**A**) When compared with *Smad7^fl/fl^* mice, MFS7KO mice have increased late mortality following nonreperfused infarction (*Smad7^fl/fl^*: 81% survival, *n =* 27; MFS7KO: 63% survival, *n =* 46; *P =* 0.0035). (**B** and **C**) Increased mortality is due to markedly accentuated death rates in male MFS7KO animals after 28 days of permanent occlusion when compared with sex-matched *Smad7^fl/fl^* mice (*Smad7^fl/fl^*: 73% survival, *n =* 15; MFS7KO: 35% survival, *n =* 20; *P =* 0.008), whereas for female MFS7KO mice, the difference in mortality does not reach statistical significance (*Smad7^fl/fl^*: 91% survival, *n =* 12; MFS7KO: 84% survival, *n =* 26; *P =* 0.58). (**D**–**I**) To determine the cause of increased mortality in MFS7KO mice, systematic postmortem histological analysis was performed by sectioning the entire heart from base to apex into 300-μm segments (2 *Smad7^fl/fl^* and 11 MFS7KO hearts from dead mice after infarction). Original magnification, ×10. (**J** and **K**) Consecutive myocardial sections studied to identify rupture sites show that only 2 of 11 MFS7KO hearts had intramural rupture track (arrows). Thus, the excess mortality in MFS7KO mice was not due to rupture. Original magnification, ×100. (**L** and **M**) In order to further understand the basis for increased mortality in MFS7KO mice, we compared echocardiographic parameters measured at the 7-day time point between the MFS7KO mice that died between 7 and 28 days and the survivors that completed the protocol. MFS7KO mice that died between 7 and 28 days had much lower left ventricular ejection fraction (LVEF) and (**N** and **O**) increased left ventricular end-diastolic volume (LVEDV) and left ventricular end-systolic volume (LVESV), suggestive of heart failure–related mortality. Survival analysis was performed using the Kaplan-Meier method. Mortality was compared using the log-rank test (**A**–**C**). Statistical comparison (**L**–**O**) was performed using Student’s *t* test (MFS7KO survivors, *n =* 37; dead MFS7KO, *n =* 7). ***P < 0.001, ****P < 0.0001. Scale bars: 1 mm (**D**–**I**) and 100 μm (**J** and **K**).

When compared with *Smad7^fl/fl^* mice, MFS7KO animals had significantly increased mortality following nonreperfused infarction (Figure 2A). The increased mortality was due to markedly accentuated death rates in male animals (Figure 2, B and C). In order to determine the cause of increased mortality in mice with myofibroblast-specific Smad7 loss, we prospectively performed systematic postmortem histologic analysis to determine the cause of death in a subpopulation of 40 mice undergoing 28-day permanent coronary occlusion protocols (19 *Smad7^fl/fl^*, 21 MFS7KO). In this subpopulation, 2 *Smad7^fl/fl^* and 11 MFS7KO animals died and the hearts were harvested, sectioned from base to apex, and systematically studied to identify rupture sites (Figure 2, D–K), as previously described (19). Two of 2 deaths in *Smad7^fl/fl^* mice and 9 of 11 deaths in MFS7KO animals were not associated with rupture, suggesting that increased mortality in MFS7KO was unrelated to rupture events. In order to further explore the basis for increased mortality in MFS7KO mice, we compared cardiac function and left ventricular geometry at the 7-day time point between MFS7KO mice that died between 7 and 28 days and corresponding survivors. Mice dying between 7 and 28 days exhibited markedly reduced ejection fraction and increased left ventricular end-diastolic volume (measured at the 7-day time point) when compared with animals that survived through the entire 28-day protocol (Figure 2, L–O). Thus, these findings suggest that heart failure is the main cause of death in MFS7KO animals.

### Myofibroblast-specific Smad7 loss accentuates adverse postinfarction remodeling.

**Figure 3:**
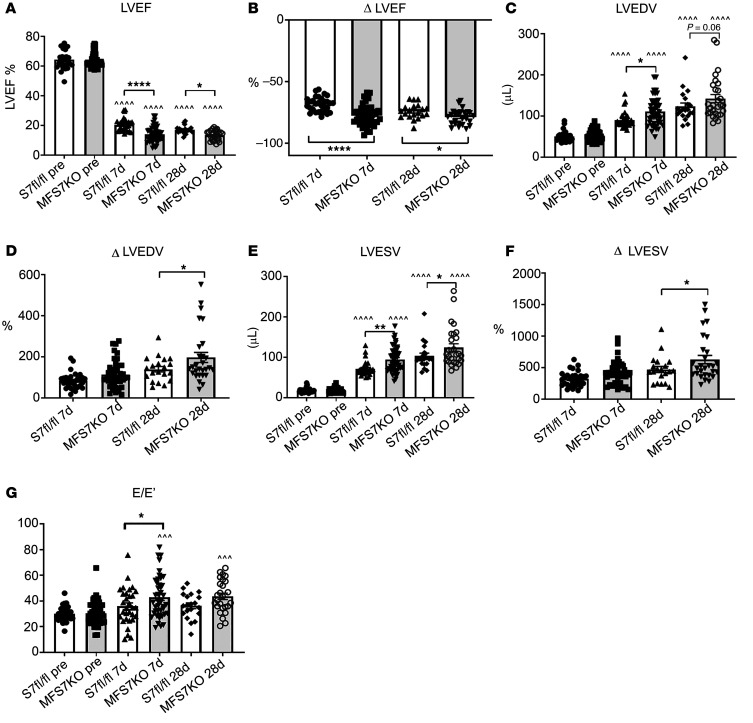
Myofibroblast-specific Smad7 loss increases left ventricular dysfunction and accentuates adverse postinfarction remodeling. (**A** and **B**) Echocardiographic assessment of adverse remodeling after 7 to 28 days following infarction shows that MFS7KO mice have worse systolic dysfunction demonstrated by a lower ejection fraction at both time points (**A**), and significantly higher reduction in ejection fraction (ΔLVEF, **B**) in comparison with *Smad7^fl/fl^* (S7fl/fl) mice. (**C**–**F**) MFS7KO mice have accentuated dilative remodeling evidenced by increased left ventricular end-diastolic volume (LVEDV, **C**), accentuated increases in LVEDV (ΔLVEDV, **D**), higher left ventricular end-systolic volume (LVESV, **E**), and accentuated ΔLVESV (**F**). (**G**) The E/E′ ratio, an indicator of diastolic dysfunction, is significantly increased in MFS7KO at the 7-day time point. Statistical comparison (**A**–**G**) was performed using 1-way ANOVA followed by Tukey’s multiple comparison test (*Smad7^fl/fl^*, *n =* 30; MFS7KO, *n =* 44). *P < 0.05, **P < 0.01, ****P < 0.0001; ^^^^^*P* < 0.001, ^^^^^^*P <* 0.0001 versus baseline.

Echocardiographic analysis showed that 7 to 28 days after MI, MFS7KO mice had worse systolic dysfunction (evidenced by lower ejection fraction) and accentuated dilative ventricular remodeling (suggested by increased end-diastolic left ventricular volumes) when compared with *Smad7^fl/fl^* animals. (Figure 3). Moreover, myofibroblast-specific Smad7 loss was associated with a higher E/E′ ratio (the ratio between early mitral inflow velocity and mitral annular early diastolic velocity) 7 days after MI, suggesting worse diastolic dysfunction (Figure 3G). Myofibroblast-specific Smad7 loss increased adverse postinfarction remodeling in both male (Supplemental Figure 4) and female mice (Supplemental Figure 5).

### Myofibroblast-specific Smad7 loss does not affect scar size, but is associated with expansion of myocardial fibrosis in the infarct border zone.

Mortality data and functional echocardiographic analysis show that myofibroblast-specific Smad7 protects the infarcted heart from adverse remodeling and reduces heart failure–related mortality. In order to explore the cellular mechanism responsible for the protective effects of myofibroblast Smad7 in the infarcted heart, we first examined whether Smad7 loss affects the size of the infarct. Systematic morphometric analysis of infarcted hearts sectioned from base to apex showed that *Smad7^fl/fl^* mice and MFS7KO animals had comparable scar sizes after 7 to 28 days of permanent occlusion (Supplemental Figure 6, A–D). Sex-specific analysis showed no significant effects of myofibroblast-specific Smad7 loss on scar size in both male and female animals (Supplemental Figure 7, A and B). Moreover, triphenyltetrazolium chloride (TTC) staining showed no significant effect of myofibroblast-specific Smad7 loss on acute infarct size 48 hours after coronary occlusion (Supplemental Figure 6, E–G).

**Figure 4:**
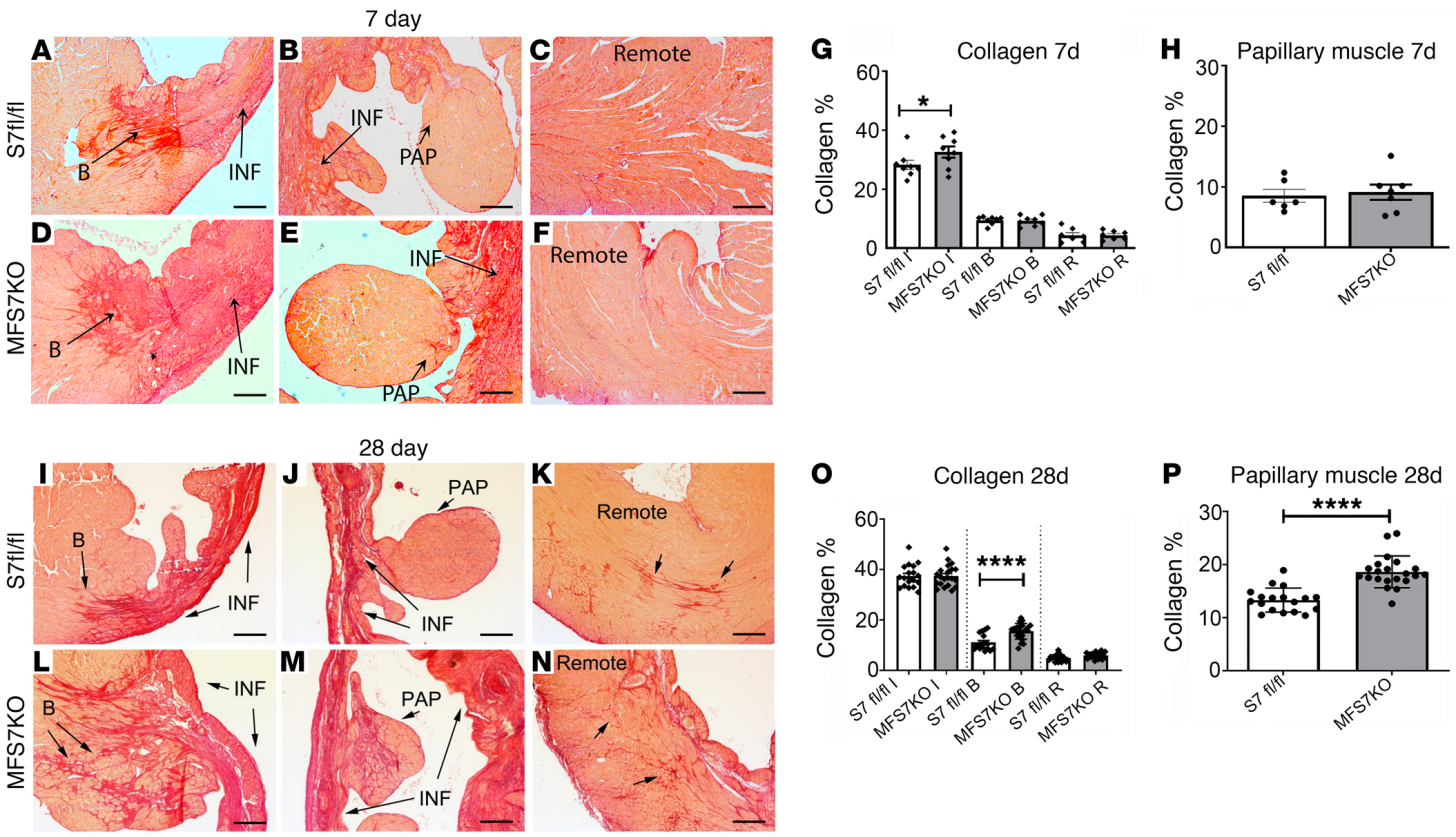
Myofibroblast-specific Smad7 loss accentuates postinfarction fibrosis in the border zone and in the papillary muscles. Collagen staining was performed using picrosirius red, and the collagen-stained area was assessed in the papillary muscle (PAP), infarcted (INF), border (B), and remote remodeling areas of *Smad7^fl/fl^* (S7 fl/fl) and MFS7KO hearts at 7 (**A**–**F**) and 28 days (**I**–**N**) of coronary occlusion. (**G** and **H**) Quantitative analysis demonstrates increased collagen deposition in the infarct zone of MFS7KO hearts compared with *Smad7^fl/fl^* at 7 days after infarction. (**O** and **P**) Twenty-eight days after infarction, increased collagen deposition is noted in the infarct border zone (**L**) and in the papillary muscle (**M**) of MFS7KO hearts compared with *Smad7^fl/fl^*, with comparable collagen levels in the infarct zone and the remote myocardium. For comparisons between multiple groups (**G** and **O**), 1-way ANOVA was performed followed by Tukey’s multiple comparison test. For comparisons between 2 groups (**H** and **P**), unpaired 2-tailed Student’s *t* test with Welch’s correction for unequal variances was performed (7 days *Smad7^fl/fl^*, *n =* 6; MFS7KO, *n =* 7; 28 days *Smad7^fl/fl^*, *n =* 18; MFS7KO, *n =* 21). *P < 0.05; ****P < 0.0001. Scale bars: 100 μm.

Because myofibroblasts are the predominant matrix-secreting and matrix-remodeling cells in the infarct, we examined the effects of myofibroblast-specific Smad7 loss on collagen content in the infarcted area, infarct border zone, and in the remote remodeling myocardium. Seven days after infarction, MFS7KO mice had significantly higher collagen content in the infarct zone when compared with *Smad7^fl/fl^* animals (Figure 4, A–H). Twenty-eight days after coronary occlusion, MFS7KO mice had increased collagen deposition in the infarct border zone (Figure 4L), but comparable collagen content in the infarcted and remote remodeling myocardium. Moreover, fibrosis was significantly increased in the papillary muscles of MFS7KO mice (Figure 4M). The profibrotic effects of myofibroblast-specific Smad7 loss were noted in both male and female mice (Supplemental Figure 7, C and D). The findings suggested that myofibroblast Smad7 protects by restraining fibrotic remodeling in the infarct border zone.

### Myofibroblast-specific Smad7 loss increased myofibroblast and matrifibrocyte density in the infarcted myocardium.

Next, we examined whether expansion of fibrosis in infarcted MFS7KO animals is associated with accentuated infiltration of the infarct and infarct border zone with activated myofibroblasts. Myofibroblasts were identified as α-SMA–expressing cells located outside the vascular wall. Myofibroblast density in infarcted myocardium peaks after 7 days of coronary occlusion and is significantly reduced as the scar matures, after 28 days of coronary occlusion (20). MFS7KO mice had a modest but significant increase in myofibroblast density in the infarcted myocardium after 7 days of coronary occlusion (Supplemental Figure 8, A–C). In contrast, myofibroblast density in the remote remodeling myocardium was not affected by the absence of Smad7. In order to examine whether the increased infiltration of the infarct with myofibroblasts in MFS7KO mice is due to effects on proliferation of these cells, we performed dual immunofluorescence with α-SMA and the proliferation marker Ki67. No significant differences in myofibroblast proliferation were noted between groups (Supplemental Figure 8, D–F). Moreover, TUNEL staining showed that increased myofibroblast infiltration in MFS7KO infarcts was not due to reduced apoptosis (Supplemental Figure 8, G–I).

During scar maturation, infarct fibroblasts and myofibroblasts transition to matrifibrocytes, specialized fibroblasts that synthesize genes typically expressed by chondroblasts and osteoblasts, such as *Comp, Chad*, and *Clip2* (5). In order to examine whether Smad7 loss affects matrifibrocyte transition in the infarcted myocardium, we compared the density of COMP^+^ matrifibrocytes between MFS7KO and *Smad7^fl/fl^* infarcts after 28 days of coronary occlusion. MFS7KO mice had significantly increased matrifibrocyte density in the mature scars, when compared with *Smad7^fl/fl^* controls (Supplemental Figure 9). Next, we examined whether Smad7 loss also affects the gene expression profile of matrifibrocytes. We sorted CD31^–^CD45^–^ cells harvested from MFS7KO and *Smad7^fl/fl^* infarcts and compared expression of genes associated with fibroblast activation, myofibroblast conversion, and matrifibrocyte transition. Cells lacking Smad7 had increased expression of *Col1a1* (collagen type I α1) and accentuated expression of the matrifibrocyte genes *Comp* and *Chad*, but comparable expression of the myofibroblast marker *Postn* (periostin) (Supplemental Figure 10).

Because fibroblasts have been implicated in regulation of angiogenesis (21), we compared the microvascular density between MFS7KO and *Smad7^fl/fl^* infarcts using CD31 staining. Myofibroblast-specific Smad7 loss did not affect microvessel density in the infarct zone and in the remote remodeling myocardium (Supplemental Figure 11).

### Smad7 loss accentuates expression of structural and matricellular matrix genes and increases synthesis of matrix-preserving Timps.

In order to explore the effects of Smad7 on fibroblast activity, we used adenovirus-mediated Cre overexpression to delete Smad7 in fibroblasts harvested from *Smad7^fl/fl^* hearts, and Smad7 overexpression studies. Smad7 loss and overexpression was documented using qPCR and Western blotting (Supplemental Figure 12).

**Figure 5:**
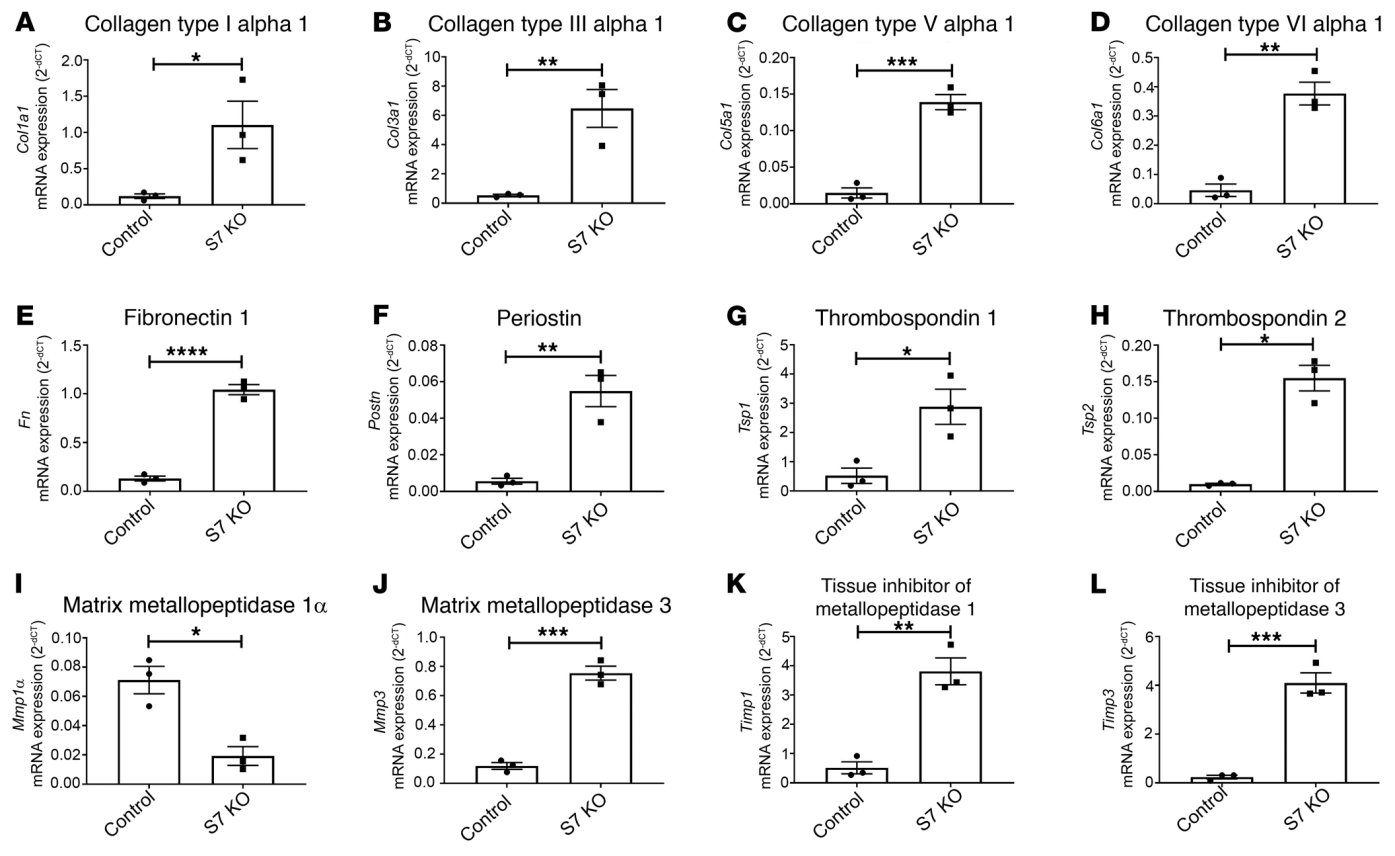
Smad7 loss accentuates synthesis of structural and matricellular genes by cardiac fibroblasts and modulates expression of MMPs and TIMPs. Expression of extracellular matrix genes was assessed using a PCR array and was compared between *Smad7*-KO fibroblasts (S7 KO, induced through overexpression of adeno-Cre in *Smad7^fl/fl^* cells) and control fibroblasts (*Smad7^fl/fl^*). (**A**–**H**) Smad7 loss accentuates fibroblast expression of genes encoding structural matrix proteins, including collagen type I α1, collagen type III α1, collagen type V α1, collagen type VI α1, and fibronectin, and matricellular proteins, such as periostin, thrombospondin 1, and thrombospondin 2. (**I**–**L**) Smad7 also modulates expression of genes associated with matrix remodeling, such as matrix metalloproteinases (MMPs) and tissue inhibitors of metalloproteinase (TIMPs). *Smad7*-KO cells have lower expression of *Mmp1α*, but markedly higher expression of *Mmp3*, *Timp1*, and *Timp2*. Comparisons between 2 groups (**A**–**L**) was performed by unpaired, 2-tailed Student’s *t* test with Welch’s correction for unequal variances (*n =* 3/group). *P < 0.05, **P < 0.01, ***P < 0.001, ****P < 0.0001.

A PCR array (Supplemental Figure 13) demonstrated that Smad7 loss in fibroblasts is associated with markedly accentuated synthesis of mRNAs encoding structural matrix proteins (including *Col1a1*, *Col3a1* [collagen type III α1], *Col5a1* [collagen type V α1], *Col6a1* [collagen type VI α1], and *Fn* [fibronectin]; Figure 5, A–H), basement membrane genes (including those encoding laminins and collagen IV chains; Supplemental Figure 14, A–G), and matricellular proteins (including *Postn*, *Tsp1* [thrombospondin 1], *Tsp2*, *Ccn2* [cellular communication network factor 2], and *Vcan* [versican]; Figure 5, A–H, and Supplemental Figure 14). Smad7 loss also accentuated fibroblast expression of *Itgb1* (Supplemental Figure 14I), which encodes a TGF-β–inducible surface protein (integrin β1) with an important role in fibroblast activation (22) and proliferation (23). Moreover, Smad7 loss had profound effects on expression of genes associated with matrix remodeling, reducing *Mmp1a* levels and increasing expression of *Mmp2*, *Timp1*, *Timp2*, and *Adamts* family members (Supplemental Figure 15). In order to study the effects of fibroblast Smad7 loss on matrix remodeling, we compared collagen synthesis and denaturation in collagen I lattices populated with *Smad7*-KO or control fibroblasts. In TGF-β1–stimulated cells, Smad7 absence increased collagen III labeling (reflecting increased de novo collagen synthesis and deposition), but also enhanced labeling with collagen hybridizing peptide (CHP), a marker of collagen denaturation (ref. 24 and Supplemental Figure 16).

### Smad7 overexpression attenuates myofibroblast conversion and reduces collagen I and fibronectin synthesis, without affecting collagen III levels.

**Figure 6:**
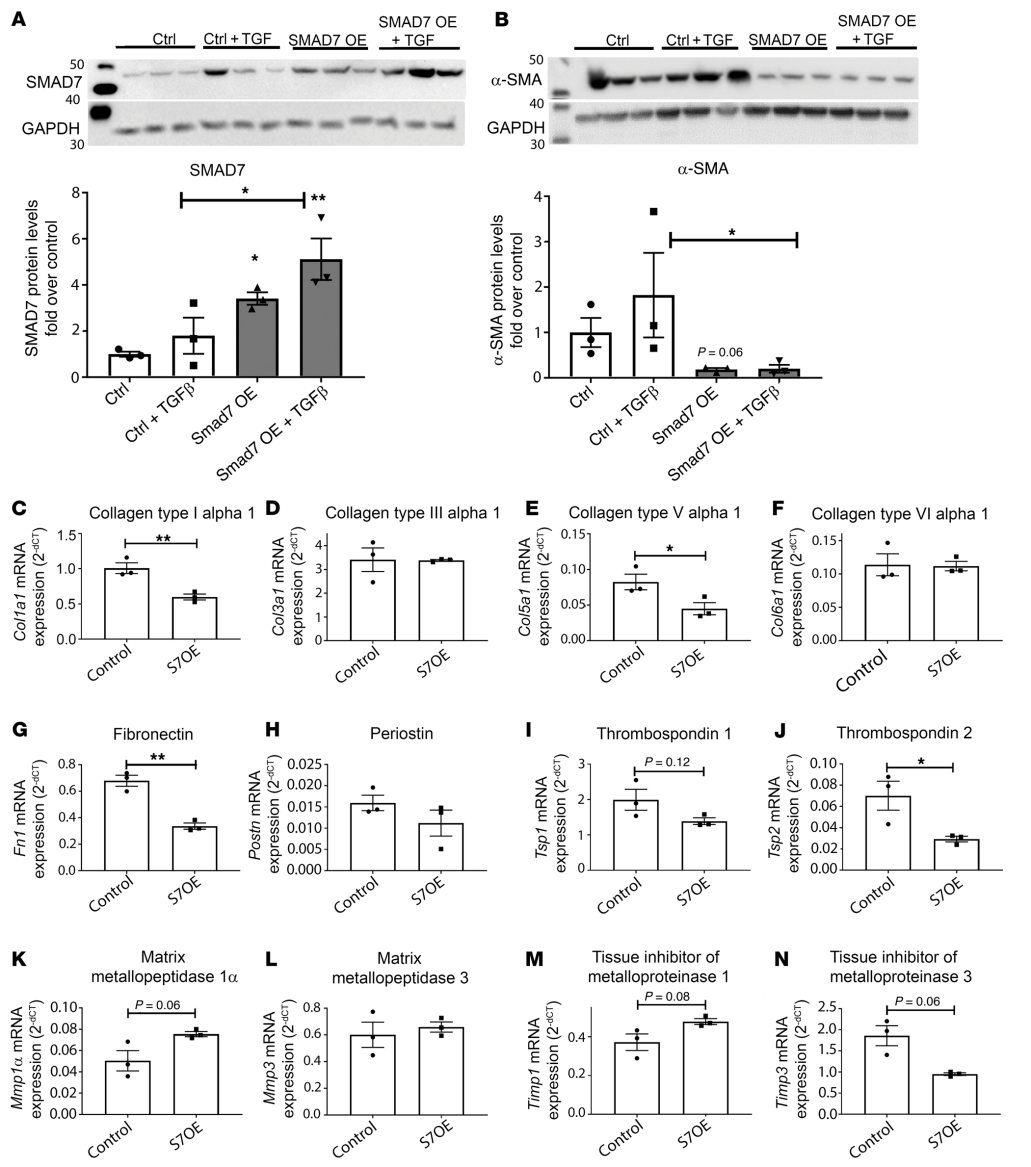
Smad7 overexpression restrains the TGF-β–induced increase in α-SMA and attenuates collagen I and fibronectin expression, without affecting collagen III levels. (**A**) Efficiency of the Smad7 overexpression (OE) strategy was assessed by comparing Smad7 protein levels between cardiac fibroblasts transfected with a *Smad7* cDNA plasmid (S7OE) and cells transfected with a control entry vector (Control). (**B**) Smad7 overexpression restrains myofibroblast conversion, markedly attenuating α-SMA protein levels in basal and TGF-β–induced conditions. (**C**–**N**) A PCR array shows that Smad7 overexpression (S7OE) attenuates synthesis of *Col1a1* (**C**), *Fn* (**G**), and *Tsp2* (**J**) without affecting C*ol3a1* transcription (**D**). (**K**–**N**) In contrast, expression of matrix remodeling genes, such as those encoding MMPs and TIMPs, is modestly affected by Smad7 overexpression. For comparisons between multiple groups (**A** and **B**), 1-way ANOVA was performed followed by Tukey’s post hoc test. For comparisons between 2 groups (**C**–**N**), unpaired, 2-tailed Student’s *t* test was performed with Welch’s correction for unequal variances (*n =* 3 per group). *P < 0.05; **P < 0.01.

Western blotting experiments showed that Smad7 overexpression inhibits acquisition of a myofibroblast phenotype in TGF-β1–stimulated fibroblasts, reducing expression of α-SMA protein (Figure 6, A and B). A PCR array (Supplemental Figure 17) showed that Smad7 overexpression attenuated synthesis of mRNAs encoding collagen type I α1, fibronectin, and thrombospondin 2 (Figure 6, C, G, and J) without affecting *Col3a1* transcription (Figure 6D). Moreover, Smad7 overexpression had modest effects on synthesis of matrix remodeling genes, such as *Mmps*, *Timps* and *Adamts* family members (Figure 6, K–N, and Supplemental Figure 18), but reduced synthesis of *Ccn2* and *Itgb1* (Supplemental Figure 19).

Overall, the *Smad7*-KO and -overexpression experiments suggest that Smad7 restrains expression of structural and matricellular matrix genes (Supplemental Figure 20). For some of the genes (pattern 2, red), the endogenous expression of Smad7 in cultured myofibroblasts is sufficient to maximally restrain gene expression, whereas in many other genes (pattern 1, blue), forced overexpression further reduces gene expression. Only one gene (*Lama2*, pattern 3, green) showed a pattern consistent with inhibitory effects of endogenous Smad7, accompanied by induction upon forced overexpression.

### Smad7 restrains TGF-β–mediated Smad2/3, ERK, and AKT signaling without affecting TβR activation.

**Figure 7:**
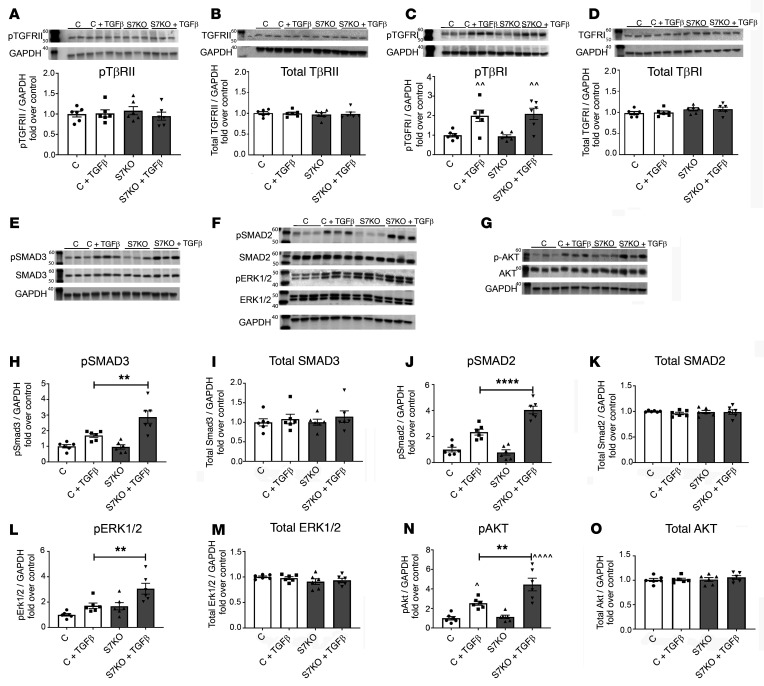
Smad7 acts downstream of the TGF-β receptors, restraining direct TGF-β–induced activation of Smad-dependent and Smad-independent signaling. (**A**) Smad7 loss does not affect activity and expression (**B**) of the constitutively active TGF-βRII. Moreover, Smad7 loss has no effects on activity (**C**) and expression (**D**) of the TGF-β–activated TGF-βRI. In order to examine the effects of Smad7 loss on Smad-dependent and -independent pathways, we performed Western blotting for p-Smad3, Smad3, p-Smad2, Smad2, p-ERK1/2, ERK1/2, p-AKT, and AKT (**E**–**G**). TGF-β–stimulated p-Smad3 (**H**) and p-Smad2 (**J**) activation was accentuated in *Smad7*-KO (S7KO) fibroblasts, in the absence of any effects on total Smad3 (**I**) or Smad2 levels (**K**). Smad-independent ERK1/2 (**L**) and AKT (**N**) activation, induced by TGF-β, was also accentuated in *Smad7*-KO fibroblasts without affecting total ERK (**M**) and AKT levels (**O**). Statistical comparison (**A**–**D** and **H**–**O**) was performed using 1-way ANOVA followed by Tukey’s multiple comparison test (*n =* 6 per group). **P < 0.01, ****P < 0.0001; ^^^^*P <* 0.01, ^^^^^^*P <* 0.0001 vs. unstimulated control.

Next, we investigated the molecular mechanism responsible for the deactivating effects of Smad7 on infarct myofibroblasts. Smad7 has been suggested to act as an endogenous inhibitor of TGF-β superfamily members; however, the level of interaction between Smad7 and the TGF-β signaling cascades (Smad-dependent and Smad-independent) is controversial. It has been suggested that Smad7 may directly associate with TβRs and inhibit TβRI kinase activity, interfere with R-Smad–TβR binding (25–27), or inhibit formation of the R-Smad–Smad4 complex (28). In order to identify the level of interaction between Smad7 and the TGF-β signaling cascade, we examined the effects of Smad7 loss on direct activation of TGF-β–mediated Smad-dependent and Smad-independent signaling. Smad7 loss accentuated Smad3 and Smad2 activation after 30 minutes of stimulation with TGF-β1 (Figure 7, H–K), without affecting activation of TβRII and TβRI (Figure 7, A–D). Moreover, Smad7 loss markedly enhanced ERK MAPK and AKT signaling in response to TGF-β (Figure 7, L–O). These findings suggest that Smad7 acts downstream of the TβRs, restraining activation of Smad-dependent signaling and Smad-independent ERK and AKT cascades.

In order to investigate the long-term interactions between Smad7 and the TGF-β signaling cascade, we studied effects of Smad7 loss on Smad2/3 and on non-Smad signaling pathways after 24 hours of TGF-β1 stimulation. As anticipated, Smad7 loss did not affect phosphorylation of the (constitutively active) TβRII (Supplemental Figure 21, A and B), but significantly increased Smad2 and Smad3 activation, consistent with the direct effects of Smad7 on R-Smad activation suggested by the 30-minute stimulation experiment (Supplemental Figure 21, H–K). However, after 24 hours of stimulation, Smad7 loss also accentuated TβRI activity without affecting total TβRI levels (Supplemental Figure 21, C and D). Considering the absence of a direct effect of Smad7 loss on TβRI activity at the 30-minute time point, the effects at 24 hours likely reflect indirect consequences of Smad7 loss on TGF-β signaling cascades. Moreover, AKT and ERK MAPK activation was not affected by Smad7 loss after 24 hours of TGF-β1 stimulation (Supplemental Figure 21, L–O), supporting the notion that the inhibitory effects of Smad7 on non-Smad pathways are transient.

### Effects of Smad7 loss on the transcriptome of cardiac fibroblasts.

In addition to its inhibitory effects on the TGF-β system, Smad7 may also act through TGF-β–independent mechanisms. In order to explore the actions of Smad7 in an unbiased manner, we performed transcriptomic analysis of the effects of Smad7 loss in cardiac fibroblasts using RNA sequencing (RNA-Seq), followed by bioinformatic analysis.

In unstimulated cardiac fibroblasts, Smad7 loss resulted in differential expression of 2297 genes (1107 upregulated, 1190 downregulated) (Supplemental File 1). In TGF-β1–stimulated *Smad7*-KO cells, 4566 genes were differentially expressed (2409 genes were upregulated and 2157 were downregulated), highlighting the important role of Smad7 in modulating phenotype and function of activated fibroblasts (Supplemental File 2 and Supplemental Figure 22).

Comparison of the transcriptome of unstimulated WT versus *Smad7*-KO cells using the Reactome Pathway Database (https://reactome.org/; accessed February 3, 2020) identified 6 differentially expressed categories. Of these, only “Signaling by receptor tyrosine kinases (RTKs)” was related to intracellular signaling pathways (*P*_adj_ = 0.037) (Supplemental Figure 23A). Comparison of the transcriptome of TGF-β–stimulated WT versus *Smad7*-KO cells using the Reactome Pathway Database identified 53 categories (Supplemental Figure 23B). Of these, 8 categories were related to intracellular signaling cascades and were represented by more than 25 differentially regulated genes (Table 1). Because differential expression of genes associated with RTK signaling was found in both unstimulated and TGF-β–stimulated cells, we reasoned that Smad7 may interact with RTK cascades in a TGF-β–independent manner.

### Effects of Smad7 on RTK activation in cardiac fibroblasts.

**Figure 8:**
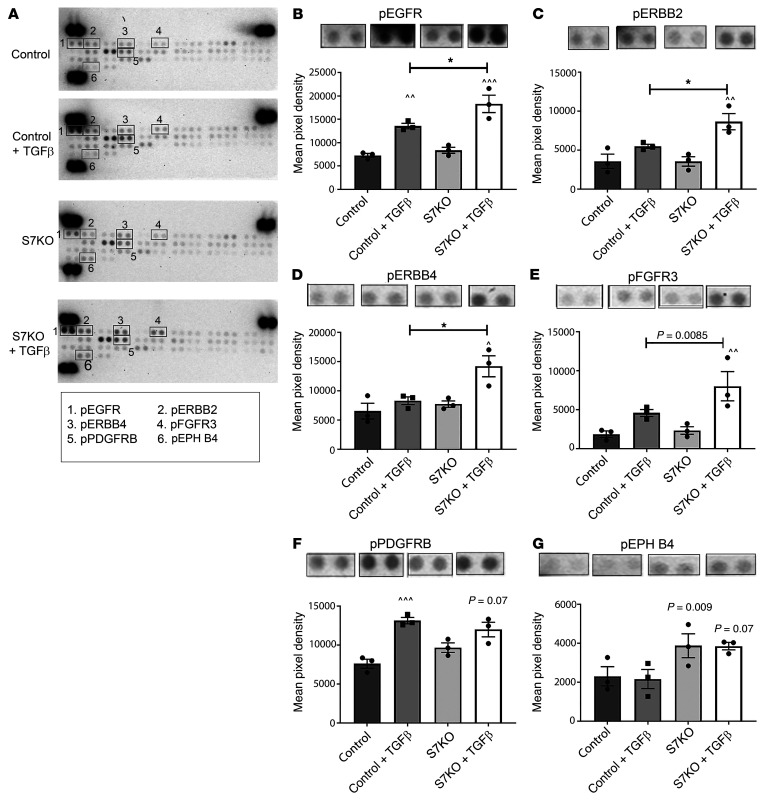
Effects of Smad7 on RTK activation. Because transcriptomic analysis identified receptor tyrosine kinase (RTK) signaling as the top-ranked pathway exhibiting differential gene expression in the absence of Smad7, we used a phospho-RTK proteomic array to identify specific RTKs modulated by Smad7. *Smad7*-KO (S7KO) fibroblasts were compared with control cells in the presence or absence of TGF-β1. (**A**) Representative array images for each condition are shown and phospho-RTKs with significant differences are highlighted in the numbered boxes. (**B**–**E**) Quantitative analysis suggests that Smad7 loss is associated with accentuated activation of EGFR/ErbB1, ErbB2, ErbB4, and FGFR cascades in TGF-β1–stimulated fibroblasts. (**F** and **G**) Other phospho-RTKs such as PDGFRβ and EPH-4 are not affected by Smad7 loss. Intensity of signal was quantified in duplicate as mean pixel density. Statistical comparison (**B**–**G**) was performed using 1-way ANOVA followed by Tukey’s multiple comparison test (*n =* 3). *P < 0.05; ^^^*P <* 0.05, ^^^^*P <* 0.01, ^^^^^*P <* 0.001 vs. unstimulated condition.

In order to identify the specific RTK signaling pathway that is modulated by Smad7, we used an RTK proteomic array (Figure 8). Our findings suggested that Smad7 loss is associated with accentuated activation of EGFR/ErbB1 (Figure 8B), ErbB2 (Figure 8C), and ErbB4 (Figure 8D) cascades in TGF-β–stimulated fibroblasts.

### Smad7 restrains ErbB2 signaling in vitro and in vivo, in a ligand-independent manner.

**Figure 9:**
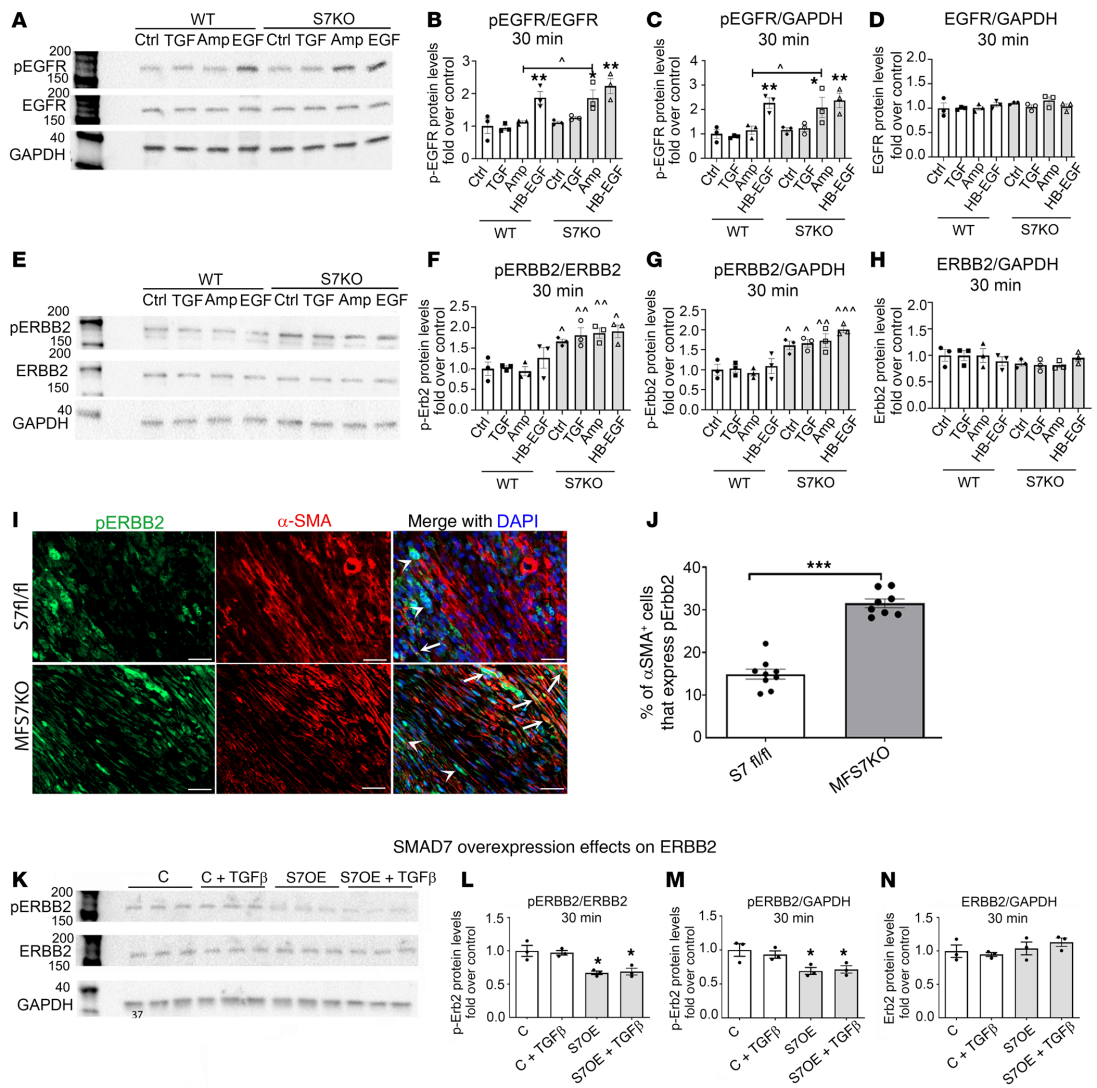
Smad7 restrains ErbB2 activation in a ligand-independent manner and limits amphiregulin-mediated EGFR/ErbB1 activation. Representative blots and quantitative analysis of the effects of Smad7 on EGFR/ErbB1 (**A**–**D**) and ErbB2 activation (**E**–**H**) in the presence or absence of TGF-β1 and the ErbB activators amphiregulin and HB-EGF (30-minute stimulation). (**A**–**D**) Smad7 loss does not affect EGFR/ErbB1 activity at baseline or after stimulation with TGF-β1 or HB-EGF. However, Smad7 loss accentuates amphiregulin-mediated EGFR activation. (**E**–**H**) Markedly increased ErbB2 activation is observed in *Smad7*-KO (S7KO) fibroblasts both at baseline and upon stimulation with TGF-β1, amphiregulin, and HB-EGF. Total ErbB1 or ErbB2 levels are not affected. (**I**) Dual immunofluorescence combining α-SMA and p-ErbB2 staining was used to identify α-SMA^+^ myofibroblasts expressing p-ErbB2 (arrows) in the infarcted myocardium (7-day permanent occlusion) of *Smad7^fl/fl^* and MFS7KO mice. p-ErbB2 is also expressed in non-myofibroblasts (arrowheads). (**J**) Quantitative analysis shows that MFS7KO hearts have a significant increase in p-ErbB2^+^ infarct myofibroblasts, when compared with control floxed infarcted hearts. (**K**–**N**) Smad7 overexpression (S7OE) attenuates ErbB2 activation in the presence or absence of TGF-β1, without affecting total ErbB2 levels (control, C). For comparisons between multiple groups (**B**–**D**, **F**–**H**, and **L**–**N**), 1-way ANOVA was performed followed by Tukey’s multiple comparison test (*n =* 3). *P < 0.05, **P < 0.01 vs. corresponding unstimulated condition; ^^^*P <* 0.05, ^^^^*P <* 0.01, ^^^^^*P <* 0.001 vs. corresponding WT. For comparisons between 2 groups (**J**), unpaired, 2-tailed Student’s *t* test was performed (*Smad7^fl/fl^*, *n =* 8; MFS7KO, *n =* 9). ***P < 0.001. Scale bars: 20 μm.

Next, we used Western blotting to investigate the effects of Smad7 on EGFR/ErbB1, ErbB2, and ErbB4 signaling in the presence or absence of TGF-β1 and the ErbB activators amphiregulin and heparin-binding EGF-like growth factor (HB-EGF) (30-minute stimulation). Smad7 loss did not affect EGFR activity at baseline or after stimulation with TGF-β1 or HB-EGF. However, Smad7 loss accentuated amphiregulin-mediated EGFR activation (Figure 9, A–D). Smad7 loss was associated with markedly increased ErbB2 activation both at baseline and upon stimulation with TGF-β1, HB-EGF, and amphiregulin (Figure 9, E–H). Similar observations were noted when the stimulation interval was extended to 2 hours (Supplemental Figure 24). In order to examine whether the effects of Smad7 on ErbB2 activity are independent of TGF-β, we assessed ErbB2 activity in *Smad7*-KO and WT cells, in the presence or absence of the TβR inhibitor SB431542, which inhibits ALK4/-5/-7 signaling (29). ErbB2 phosphorylation was increased in *Smad7*-KO cells, in the presence or absence of TGF-β signaling disruption (Supplemental Figure 25). In contrast, as expected, the ALK inhibitor abrogated R-Smad signaling. ErbB2 has no known extracellular ligands, but functions as a co-receptor that heterodimerizes with other activated ErbB family members to transduce signaling cascades (30). Thus, the findings demonstrate a ligand-independent inhibitory effect of Smad7 on ErbB2, and suggest that in addition to its actions in inhibition of TGF-β–stimulated cascades, Smad7 also restrains EGFR/ErbB2 activation in a TGF-β–independent manner. In contrast, Smad7 did not affect ErbB4 activation in the presence or absence of TGF-β1 or ErbB ligands (Supplemental Figure 26). In order to examine whether Smad7 loss regulates ErbB2 activity in vivo, we performed immunofluorescence and compared the density of p-ErbB2–expressing myofibroblasts between *Smad7^fl/fl^* and MFS7KO mice. Myofibroblast-specific loss of Smad7 markedly increased the density of infarct myofibroblasts that expressed p-ErbB2 (Figure 9, I and J). Next, we examined whether Smad7 overexpression affects ErbB2 activation in cardiac fibroblasts. Smad7-overexpressing cells had decreased ErbB2 phosphorylation both at baseline and upon stimulation with TGF-β1 (Figure 9, K–M). Total ErbB2 expression was not affected by Smad7 overexpression (Figure 9N).

### Smad7 restrains synthesis of fibrosis-associated genes, at least in part, through TGF-β–independent ErbB1 and -2 inhibition.

**Figure 10:**
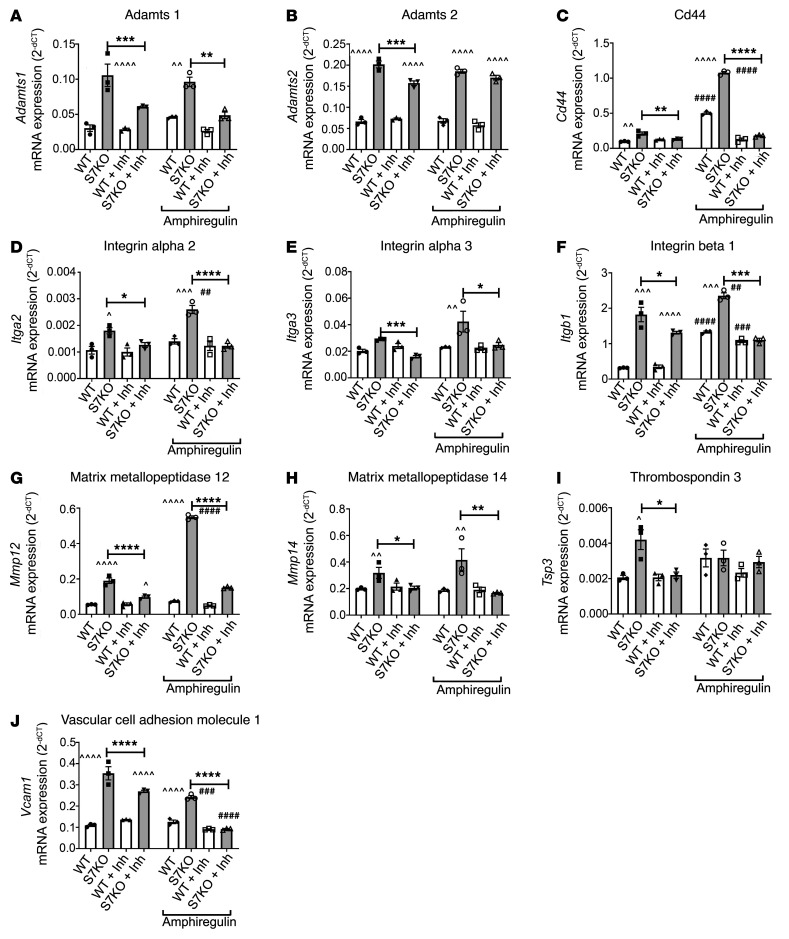
The effects of Smad7 on fibroblast activity are mediated, at least in part, through an interaction with ErbB1/2. Comparison of the effects of the dual ErbB1/2 inhibitor lapatinib (Inh) on extracellular matrix gene expression in *Smad7*-KO and WT fibroblasts, in the presence or absence of amphiregulin. (**A**–**J**) Unstimulated cardiac fibroblasts (first 4 bars of each graph): ErbB1/2 inhibition in unstimulated cardiac fibroblasts abrogates the effects of Smad7 loss on expression of *Adamts1*, *Adamts2*, *Mmp12*, and *Mmp14* (proteases); *Itga2*, *Itga3*, and *Itgb1* (integrins); *Tsp3* (matricellular protein); and *Cd44* and *Vcam-1* (adhesion molecules), without exerting any effects on WT cells. Amphiregulin-stimulated cardiac fibroblasts (last 4 bars of each graph): In the presence of amphiregulin, the effects of Smad7 loss on *Cd44*, *Itga2*, *Itgb1*, and *Mmp12* synthesis are accentuated. ErbB1/2 inhibition markedly attenuates the effects of Smad7 loss. Statistical comparison (**A**–**J**) was performed using 1-way ANOVA followed by Tukey’s multiple comparison test (*n =* 3). *P < 0.05, **P < 0.01, ***P < 0.001, ****P < 0.0001; ^^^*P <* 0.05, ^^^^*P <* 0.01, ^^^^^*P <* 0.001, ^^^^^^*P <* 0.0001 vs. corresponding WT; ^##^*P <* 0.01, ^###^*P <* 0.001, ^####^*P <* 0.0001 vs. unstimulated.

In order to examine the significance of the Smad7/ErbB2 interaction in modulating fibroblast gene expression, we compared the effects of the dual ErbB1/2 inhibitor lapatinib on WT and *Smad7*-KO cells using a PCR array (Supplemental Figures 27 and 28). ErbB1/2 inhibition in unstimulated cardiac fibroblasts abrogated or attenuated the effects of Smad7 loss on expression of *Adamts1*, *Adamts2*, *Mmp12*, and *Mmp14* (encoding proteases), *Itga2*, *Itga3*, and *Itgb1* (integrins), *Tsp3* (matricellular protein), and *Cd44* and *Vcam1* (adhesion molecules) (Figure 10), without exerting any effects on WT cells. The findings suggest that Smad7 restrains synthesis of several fibrosis-associated genes, at least in part through an interaction with ErbB1/ErbB2. Because Smad7 loss markedly increases baseline ErbB2 activation (without affecting ErbB1/EGFR baseline activity), we reasoned that the effects of the dual inhibitor are mediated through ErbB2 inhibition. Similar findings were noted in experiments examining the effects of the inhibitor on cells stimulated with the ErbB1/EGFR ligand amphiregulin. In the presence of amphiregulin, the effects of Smad7 loss on *Cd44*, *Itga2*, *Itgb1*, and *Mmp12* synthesis were accentuated. ErbB1/2 inhibition markedly attenuated the effects of Smad7 loss (Figure 10).

Amphiregulin stimulation increased the impact of the antifibrotic interaction between Smad7 and ErbB1/2. In amphiregulin-stimulated cells, ErbB1/2 inhibition abrogated the effects of Smad7 on synthesis of key structural extracellular matrix genes (such as *Col1a1*, *Col3a1*, and *Fn*), nonstructural matrix genes (*Ecm1* and *Col5a1* and the matrix-preserving antiproteases *Timp1* and *Timp2*) (Supplemental Figure 29). In contrast, Smad7-mediated suppression of other fibrosis-associated genes (including *Col6a1*, *Mmp3*, *Postn*, *Spp1* [osteopontin], and *Vcan*) was not affected by ErbB1/2 inhibition, in the presence or absence of amphiregulin (Supplemental Figure 30).

The data suggest that in addition to its effects in restraining the TGF-β cascade, Smad7 also inhibits ErbB2 responses. Considering the role of ErbB2 in mediating sustained effects of ErbB ligands in fibrotic conditions, this TGF-β–independent action of Smad7 may amplify its antifibrotic actions. Thus, Smad7 should be viewed beyond its role as a negative regulator of the TGF-β superfamily.

### Smad7 interacts directly with ErbB2.

**Figure 11:**
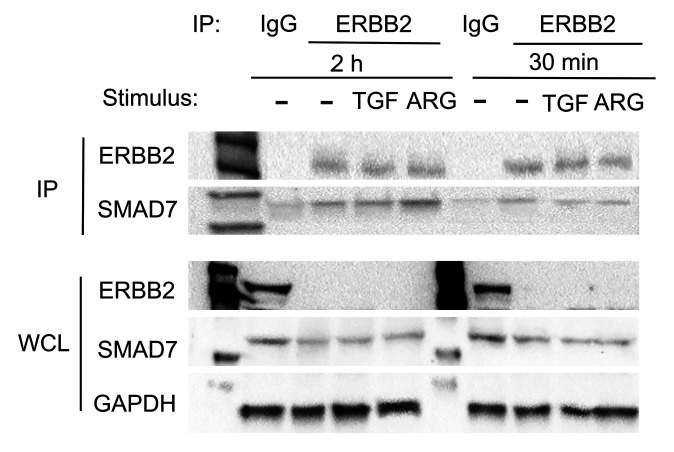
Smad7 binds to ErbB2. Western blot analysis after immunoprecipitation by anti-ErbB2 magnetic beads to demonstrate the interaction between ErbB2 and Smad7 in cardiac fibroblasts treated with TGF-β1 (10 ng/mL) or amphiregulin (ARG, 1 ng/mL). Upper panel: Immunoprecipitated (IP) ErbB2 fractions from cell lysates (upper, first band), show Smad7 expression (upper, second band), demonstrating that Smad7 interacts with ErbB2 in unstimulated and stimulated cardiac fibroblasts. Validation of the ErbB2 IP technique is shown by both the absence of ErbB2 expression in the IgG-immunoprecipitated fraction, as well as the absence of ErbB2 protein in the whole-cell lysate (WCL) of ErbB2 pull-down cells (lower panel, first band). GAPDH levels in the WCL were used as loading controls. Representative blots are shown (*n =* 3).

In order to examine whether Smad7 directly interacts with ErbB2, we performed coimmunoprecipitation experiments in fibroblast lysates in the presence or absence of TGF-β1 or amphiregulin. Using magnetic beads conjugated with ErbB2 as the bait protein, we pulled down all proteins bound to ErbB2. Western blotting on the immunoprecipitated fraction showed that Smad7 was present in all experimental conditions (Figure 11), demonstrating its physical interaction with ErbB2.

## Discussion

We report several potentially novel observations. First, Smad3-mediated Smad7 overexpression in infarct myofibroblasts is an important endogenous protective mechanism that reduces heart failure–related mortality and limits maladaptive ventricular fibrosis. Second, in cardiac fibroblasts, Smad7 restrains TGF-β–induced synthesis of fibrosis-associated genes by inhibiting both Smad-dependent and Smad-independent cascades, acting downstream of the TβRs. Third, Smad7 has important antifibrotic effects unrelated to its TGF-β inhibitory actions. Our findings document what we believe is a novel interaction between Smad7 and ErbB2 that is independent of TGF-β and mediates part of the antifibrotic effects of Smad7.

### TGF-β signaling in cardiac repair, remodeling, and fibrosis.

TGF-β cascades are centrally involved in repair and remodeling of the infarcted heart (31, 32), promoting inflammatory and apoptotic responses of cardiomyocytes (4, 33), stimulating macrophage phagocytic activity and antiinflammatory transition (34), and inducing a reparative matrix-synthesizing phenotype in fibroblasts (4). Considering the potent activating effects of TGF-β/Smad3 on fibroblasts that promote myofibroblast conversion and stimulate synthesis of extracellular matrix proteins, prolonged or unrestrained TGF-β activation would be expected to expand fibrosis, accentuating adverse remodeling and worsening heart failure. Our findings show that myofibroblast-specific Smad7 induction serves as a crucial endogenous inhibitory signal in restraining fibrotic remodeling and dysfunction following MI and reduces heart failure–related mortality.

### Smad7 expression is Smad3 dependent and is associated with myofibroblast conversion.

Smad7 overexpression was noted primarily in cells exhibiting myofibroblast conversion, and not in PDFRα^+^α-SMA^–^ fibroblasts. The selective upregulation of Smad7 in α-SMA^+^ myofibroblasts likely reflects the common TGF-β/Smad3–mediated mechanism of induction. Smad3 mediates myofibroblast conversion in TGF-β–stimulated fibroblasts (13) and is critically involved in Smad7 upregulation in both stimulated fibroblasts and infarct myofibroblasts (Supplemental Figure 1). However, the basis for the heterogeneous responses of infarct fibroblasts to the fibrogenic environment of the infarct, which is characterized by marked upregulation of TGF-βs, is more difficult to explain. Why is myofibroblast conversion limited to only a subset of fibroblasts? Differences in microenvironmental conditions within various areas of the infarct may be responsible. It has been suggested that local activation of TGF-β may require a profibrotic niche, in which cadherin-11 junctions maintain macrophages (a major source of TGF-βs) and fibroblasts in close proximity (35). Alternatively, fibroblast subsets may exhibit differences in their responsiveness to TGF-β ligands, possibly related to distinct patterns of expression of TβRs and co-receptors. Regardless of its molecular basis, myofibroblast-specific Smad7 induction following infarction may serve to enhance the antifibrotic effects of this endogenous mediator, focusing its effects on cell subsets with the highest levels of fibrogenic capacity and matrix-synthesizing activity (36).

### The antifibrotic effects of Smad7.

Antifibrotic effects of exogenous administration or transgenic overexpression of Smad7 have been extensively reported in models that recapitulate fibrotic diseases. In a model of bleomycin-induced lung fibrosis, *Smad7* gene transfer attenuated fibrogenic activity (37). Moreover, in a model of liver fibrosis, hepatocyte-specific Smad7 expression reduced liver fibrogenesis (38). The role of Smad7 as an endogenous negative regulator of fibrosis is less convincingly established. Most of the evidence (39–42) is derived from experiments using a hypomorphic global Smad7-mutant line (Smad7^Δex1^) (43) that exhibits preserved Smad7 functions due to the presence of an intact MH2 domain, the key effector domain involved in Smad7 interactions with TβRs and R-Smads (27). Our study presents the first in vivo evidence to our knowledge suggesting a cell-specific role for Smad7 in deactivating fibroblasts. The antifibrotic effects of Smad7 are associated with attenuated myofibroblast conversion and markedly reduced expression of both structural and matricellular matrix proteins (Figure 5).

### How does Smad7 restrain TGF-β signaling cascades?

Several molecular mechanisms have been proposed to explain the inhibitory effects of Smad7 on TGF-β superfamily signaling (44). First, Smad7 lacks the C-terminal SSXS motif required for phosphorylation of R-Smads by TβRI kinase (45) and may directly associate with TβRs, inhibiting TβRI kinase activity, or may compete with R-Smads for receptor binding (25, 27). Second, Smad7 may form a complex with the transmembrane pseudoreceptor BAMBI (bone morphogenetic protein and activin membrane-bound inhibitor), inhibiting TβR-driven R-Smad activation (46). Third, Smad7 has been suggested to interfere with formation of the R-Smad–Smad4 complex, thus preventing translocation of R-Smads to the nucleus (47). Finally, Smad7 may also promote TβR and R-Smad turnover, interacting with E3 ubiquitin ligases, such as Smurf1 and Smurf2 (48–50). Our experiments suggest that in fibroblasts, Smad7 restrains TGF-β signaling through interactions downstream of the TβRs that results in reduced activation of Smad2 and Smad3 (Figure 7, A–K), without affecting TβRI or TβRII activity. Moreover, Smad7 transiently inhibits activity of non-Smad cascades, such as Erk and Akt (Figure 7, L–O).

### The antifibrotic actions of Smad7 involve TGF-β–independent inhibition of ErbB2.

In addition to its established effect as an inhibitor of TGF-β superfamily ligands, Smad7 has also been suggested to interact with other signaling cascades. For example, in epithelial cells, Smad7 may exert antiinflammatory effects by inhibiting NF-κB signaling through increased IκB expression (51, 52), and may modulate Wnt signaling through physical interactions with β-catenin (53). The in vivo significance of these interactions remains unclear. We used unbiased transcriptomic and proteomic analysis to explore the basis for Smad7-mediated effects on cardiac fibroblasts. Surprisingly, bioinformatic analysis of RNA-Seq data comparing the transcriptomic profile of TGF-β–stimulated *Smad7*-KO versus WT cells identified RTK signaling (and not TGF-β responses) as the top-ranked pathway on the basis of differential gene expression (Table 1). An RTK proteomic array followed by Western blotting showed that Smad7 restrains baseline signaling through ErbB2, a membrane-bound RTK that has no known ligands, but accentuates and prolongs responses to ErbB agonists. Pharmacologic inhibition experiments demonstrated the functional role of the Smad7-ErbB2 interactions, showing that the antifibrotic properties of Smad7 were in part due to inhibition of ErbB1/2 signaling (Figure 10). Disruption of the TGF-β signaling axis did not affect the effects of Smad7 loss on ErbB2 activity, demonstrating that the actions of Smad7 are TGF-β independent, and involve a direct interaction between Smad7 and ErbB2 (Figure 11).

Thus, antifibrotic actions of Smad7 do not reflect only suppression of TGF-β signaling cascades, but are also mediated through a direct TGF-β–independent interaction with ErbB2. We identified several key fibrogenic signals that were inhibited by Smad7 through a TGF-β–independent interaction with ErbB2. First, expression of ADAMTS1 and ADAMTS2, two members of the ADAM family of extracellular proteases, was restrained through Smad7 effects on ErbB2. Both these proteases have profibrotic actions; ADAMTS1 is involved in SPARC-mediated collagen deposition (54), whereas ADAMTS2 promotes fibrosis by enhancing maturation of the collagen network in fibrotic tissues (55). Second, synthesis of integrins with fibroblast-activating properties, such as integrin β1 and α3 (56), was suppressed through Smad7-ErbB2 interactions. Integrin β1 triggers activation of a matrix-synthesizing program in fibroblasts (57), whereas integrin α3β1 is required in lung fibrosis through actions that may involve profibrotic β-catenin signaling (58). Third, Smad7-mediated ErbB2 inhibition attenuates expression of the profibrotic cell surface molecule CD44. CD44 mediates fibrogenic actions of hyaluronan and osteopontin by accentuating TGF-β–induced activation of a matrix-synthesizing program in fibroblasts (59–61). Fourth, the Smad7-ErbB2 interaction attenuates expression of the membrane-bound metalloproteinase MMP14 in cardiac fibroblasts. In fibrotic conditions, surface expression of MMP14 in fibroblasts degrades pericellular collagen and induces an invasive migratory fibroblast phenotype that may contribute to the fibrogenic response (62, 63).

### Conclusions: the biology of Smad7 beyond TGF-β regulation.

**Figure 12:**
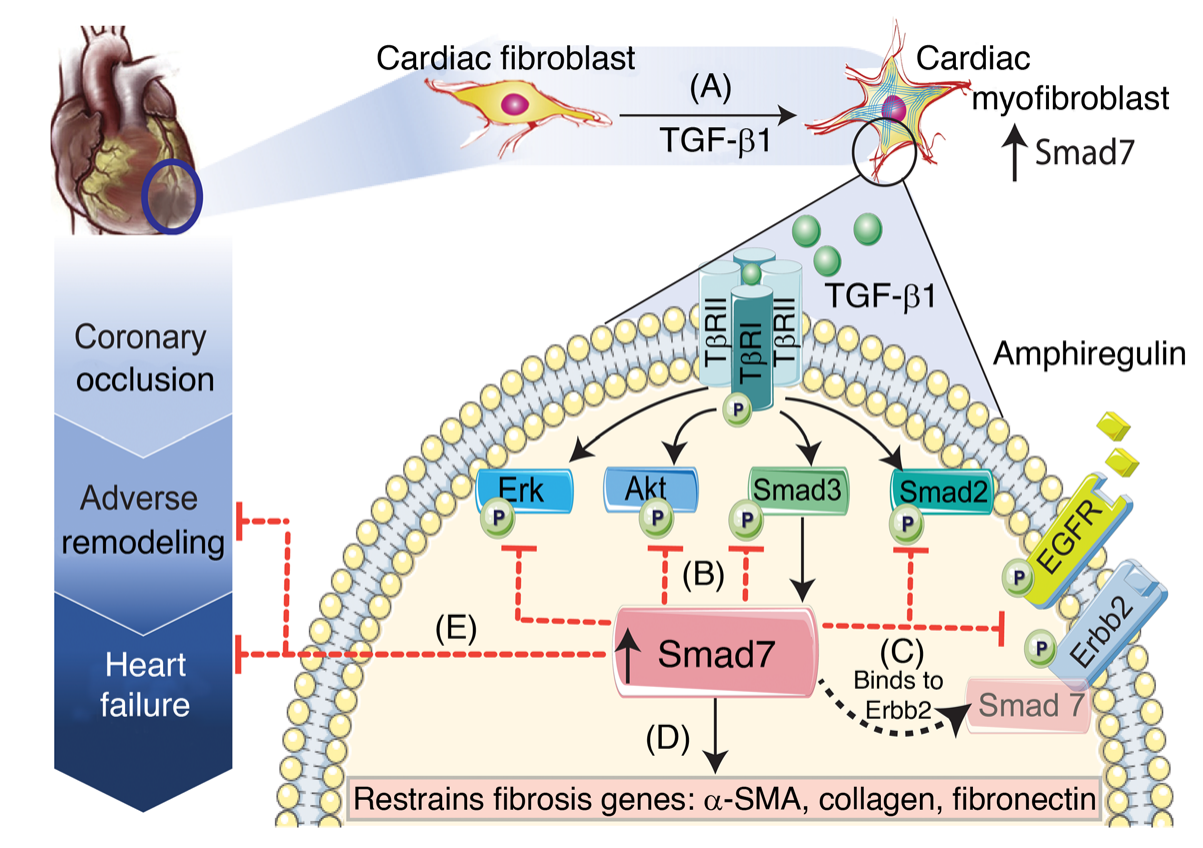
Schematic illustration of the findings of the study. Our study shows that (**A**) in healing infarcts, the inhibitory Smad, Smad7, is overexpressed in activated myofibroblasts through a Smad3-dependent pathway. (**B**) Smad7 induction restrains TGF-β–mediated Smad2/3, Erk, and Akt signaling without affecting TβR activation. (**C**) In addition to its inhibitory effects on TGF-β cascades, Smad7 directly interacts with the receptor tyrosine kinase ErbB2 and restrains EGFR/ErbB2 activation in a TGF-β–independent manner. (**D**) Inhibition of both TGF-β and ErbB1/2 cascades by Smad7 restrains synthesis of structural and matricellular matrix–associated genes and attenuates myofibroblast conversion. (**E**) Myofibroblast-specific Smad7-mediated effects protect the infarcted heart from adverse remodeling and reduce heart failure–related mortality. Considering the role of ErbB2 in mediating sustained actions of other ErbBs in fibrotic conditions, the Smad7-ErbB2 interaction may amplify the antifibrotic effects of Smad7. Our findings suggest that Smad7 should be viewed beyond its role as a negative regulator of the TGF-β superfamily.

Our study reveals a molecular pathway that amplifies the antifibrotic effects of Smad7. In addition to its effects on the TGF-β superfamily, Smad7 also restrains activation of ErbB2, a crucial fibrogenic mediator in many different systems (64–66), Thus, TGF-β activation of the Smad3 cascade in fibroblasts initially transduces a potent fibrogenic program involved in repair, but also triggers Smad7-mediated negative feedback cascades with broad antifibrotic effects on both TGF-β and ErbB pathways (Figure 12). Thus, fibrogenic responses stimulated in response to injury are self-limited due to activation of negative inhibitory signals. Chronic fibrotic conditions may involve defective activation of inhibitory signals, such as Smad7 (48), leading to prolonged activation of TGF-β and ErbB signaling cascades.

## Methods

### Generation of mice with myofibroblast-specific loss of Smad7.

We generated mice with loss of Smad7 in activated myofibroblasts (MFS7KO) by breeding *Smad7^fl/fl^* mice, in which the promoter region and exon 1 are flanked by *loxP* sites (ref. 67 and The Jackson Laboratory, stock no. 017008) with a *Postn*-Cre–transgenic mouse line, in which Cre recombinase is driven by the 3.9-kb mouse *Postn* promoter (68, 69). Periostin, which is encoded by *Postn*, is not expressed in cardiomyocytes, vascular cells, hematopoietic cells, or quiescent cardiac fibroblasts (70, 71), but is upregulated in injury site–activated fibroblasts in infarcted and in pressure-overloaded hearts (72).

### Mouse model of nonreperfused MI.

A mouse model of nonreperfused MI was used, as previously described by our group (18). To assess cardiac function and remodeling following MI, animals underwent echocardiographic analysis at baseline and after 7 and 28 days of permanent coronary occlusion, using the Vevo 2100 system (VisualSonics), as previously described (73). The surgical protocols were performed by an investigator blinded to the genotype of the animals.

### Immunohistochemistry, histology, and quantitative morphometry.

For histopathological analysis, murine hearts were fixed in zinc-formalin (Z-fix; Anatech), and embedded in paraffin. Infarcted hearts were sectioned from base to apex at 250-μm intervals, thus reconstructing the whole heart, as previously described (73). Picrosirius red staining was used to label the collagen-based scar and collagen content was quantitatively assessed in the infarct region, papillary muscle, and remote remodeling myocardium at 7 and 28 days after infarction using ImagePro software (Media Cybernetics). Microvessels were identified in the infarcted and remote remodeling myocardium by using anti-CD31 immunohistochemistry to label endothelial cells. To assess the size of acute infarcts, the TTC staining method was used (74). Morphometric parameters were quantitatively assessed using Zen 2.6 Pro software (Zeiss Microscopy).

In order to systematically characterize the expression of Smad7 in fibroblasts versus myofibroblasts, triple fluorescent staining experiments were carried out in infarcted PDGFRα-GFP fibroblast reporter mice (75), combining GFP, α-SMA, and Smad7 staining. Alternative validation of Smad7 expression in activated myofibroblasts was carried out by Smad7 immunofluorescent staining experiments on infarcted *Postn*-Cre EYFP reporter mice.

Activation of ErbB2 in infarct myofibroblasts was compared between *Smad7^fl/fl^* and MFS7KO infarcts using p-ErbB2 and α-SMA dual fluorescence.

### Cardiac fibroblast isolation.

Fibroblasts were isolated from 12-week-old mouse (C57BL/6J; The Jackson Laboratory, strain 000664) hearts with protocols used in our laboratory (76).

### FACS isolation of cardiac fibroblasts from baseline or infarcted hearts.

FACS isolation of the CD31^–^C45^–^ cell population was performed on *Smad7^fl/fl^* and MFS7KO mice at baseline, or after 21 days of coronary occlusion using protocols established in our laboratory (24).

### Smad7-KO in cardiac fibroblasts by adenovirus-mediated Cre expression.

For Smad7 deletion, mouse cardiac fibroblasts isolated from *Smad7^fl/fl^* mice were infected with adenovirus expressing Cre recombinase, in order to knock out the *loxP*-flanked *Smad7* gene. Empty adenovirus was used as a transfection control.

### Smad7 overexpression.

For Smad7 overexpression experiments, mouse cardiac fibroblasts were transfected for 24 hours with a plasmid containing a Turbo-GFP–tagged mouse *Smad7* cDNA clone (Origene, MG226590).

### Experiments in cardiac fibroblast–populated collagen pads.

In order to study the effects of TGF-β ligands on Smad7 expression, cardiac fibroblasts were cultured in collagen pads as previously described (18). For experiments assessing collagen synthesis/denaturation in cardiac fibroblast–populated collagen pads, we performed dual fluorescence with an anti–collagen type III antibody to assess de novo collagen synthesis, and with a collagen-hybridizing fluorescent peptide 5-FAM conjugate (F-CHP, 3Helix, Inc.) that specifically binds to denatured unfolded collagen (24, 77).

### Smad3 in vitro and in vivo knockdown experiments.

For experiments assessing the role of Smad3 in Smad7 expression in cardiac fibroblasts in vitro, cardiac fibroblasts were transfected with either Smad3 siRNA (MISSION, NM_016769, MilliporeSigma) or nonsilencing control siRNA. In order to evaluate Smad3’s role in Smad7 expression in cardiac fibroblasts in vivo, isolation of cardiac fibroblasts from noninfarcted and infarcted areas of the heart of control and fibroblast-specific *Smad3*-KO mice (*Postn*-Cre;*Smad3^fl/fl^* mice) was performed as previously described by our group (4).

### Evaluation of Smad7 role in RTK activation.

For experiments assessing the role of Smad7 in RTK activation, control and *Smad7*-KO cardiac fibroblasts were cultured in the presence or absence of TGF-β1, amphiregulin, or HB-EGF (10 ng/mL). Cell lysates were used for protein extraction and for Western blots assessing phospho-RTK levels. In order to study the effect of RTKs on cardiac fibroblasts in modulating fibroblast gene expression, control and *Smad7*-KO cardiac fibroblasts were pretreated with the ErbB1/2 dual inhibitor lapatinib (5 μM) for 1 hour, followed by treatment with or without TGF-β1 (10 ng/mL) and amphiregulin (1 ng/mL) for 4 hours. For experiments assessing the TGF-β independence of the effects of Smad7 on ErbB2, passage 2 control and *Smad7*-KO cardiac fibroblasts were incubated for 2 hours with the ALK 4/5/7 inhibitor SB431542 (10 μM; Sigma-Aldrich, S4317) before protein harvest.

### Protein extraction and Western blotting.

Protein from whole hearts or cardiac fibroblasts was used for Western blotting using established protocols. Complete unedited blots and detailed information on the antibodies used for Western blotting are provided in the supplemental material.

### RNA extraction, PCR arrays, and qPCR.

RNA was extracted from cells and mouse hearts using TRIzol reagent (Qiagen, 79306) and was used for qPCR using custom-made primers, or commercially available PCR arrays (RT^2^ Profiler Mouse Extracellular Matrix and Adhesion Molecules PCR Array, Qiagen).

### phospho-RTK array.

The relative level of tyrosine phosphorylation of 39 different RTKs was determined in control and *Smad7*-KO cardiac fibroblasts in the absence or presence of TGF-β1 (2-hour stimulation, 10 ng/mL) using the Proteome Profiler Mouse Phospho-RTK Array Kit (R&D Systems, ARY014).

### ErbB2-Smad7 coimmunoprecipitation assay.

ErbB2-Smad7 coimmunoprecipitation was evaluated using a Dynabeads Co-Immunoprecipitation Kit (Invitrogen, 14321D), according to the protocol provided by the manufacturer. Epoxy M-270 magnetic beads were coupled overnight (4°C) with anti-ErbB2 antibody to form ErbB2-conjugated magnetic beads. Epoxy M-270 magnetic beads conjugated with IgG antibodies were used as the immunoprecipitation control. ErbB2-beads and IgG-beads were incubated with 100 mg of the protein lysate. Following consecutive washes, eluted proteins containing the purified ErbB2-bound or IgG control–bound coimmunoprecipitated proteins were assessed for Smad7 presence by Western blotting. Whole cell lysate/input sample was used as a control of the immunoprecipitation enrichment.

### Library preparation for transcriptome sequencing.

RNAs isolated from unstimulated control fibroblast, TGF-β1–treated control fibroblasts, unstimulated *Smad7*-KO fibroblasts, and TGF-β1–treated *Smad7*-KO fibroblasts, run with 3 replicates each, were sent to Novogene to construct a total of 12 cDNA libraries by using the NEBNext Ultra RNA Library Prep Kit for Illumina (New England Biolabs) and to perform RNA-Seq.

### Gene expression, differential expression, enrichment, and coexpression analysis.

Library preparations were sequenced on an Illumina HiSeq 2000, generating 100-bp paired-end reads. Read count of fragments per kilobase of transcript sequence per millions base pairs sequenced (FPKM) was used to calculate gene expression level. Cluster differential expression analysis for every gene in the 4 different cardiac fibroblast conditions was performed using the DESeq2 R software package (https://bioconductor.org/packages/release/bioc/html/DESeq2.html). Genes with an adjusted *P* value of 0.05 or less were considered to be differentially expressed. Genes were ranked by differential gene expression as log_2_(fold change) between each comparison group. Using the log_2_-transformed fold changes obtained from the differential expression analysis for every gene, gene enrichment analysis (Gene Ontology, GO) and pathway (KEGG and Reactome) analysis were performed using the ClusterProfiler software package (https://bioconductor.org/packages/release/bioc/html/clusterProfiler.html). RNA-Seq data have been deposited in NCBI’s Gene Expression Omnibus database (GEO GSE185767).

### Statistics.

For all analyses, normal distribution was tested using the Shapiro-Wilk normality test. For comparisons of 2 groups, an unpaired, 2-tailed Student’s *t* test with (when appropriate) Welch’s correction for unequal variances was performed. The Mann-Whitney test was used for comparisons between 2 groups that did not show Gaussian distribution. For comparisons of multiple groups, 1-way ANOVA was performed followed by Tukey’s multiple comparison test. The Kruskal-Wallis test followed by Dunn’s multiple comparison post hoc test was used when one or more groups did not show Gaussian distribution. Survival analysis was performed using the Kaplan-Meier method. Mortality was compared using the log-rank test. Data are expressed as mean ± SEM. Statistical significance was set at a *P* value of less than 0.05.

### Study approval.

Animal studies were approved by the Institutional Animal Care and Use Committee at Albert Einstein College of Medicine and conform to the *Guide for the Care and Use of Laboratory Animals* (National Academies Press, 2011).

## Author contributions

CH and NGF designed the study. CH, AVS, AH, LA, SCH, BC, and RL performed the experiments. SJC provided an important genetic tool. CH and NGF wrote the manuscript. CH, AVS, AH, LA, SJC, and NGF provided critical input and edited the manuscript.

## Supplementary Material

Supplemental data

## Figures and Tables

**Table 1:**
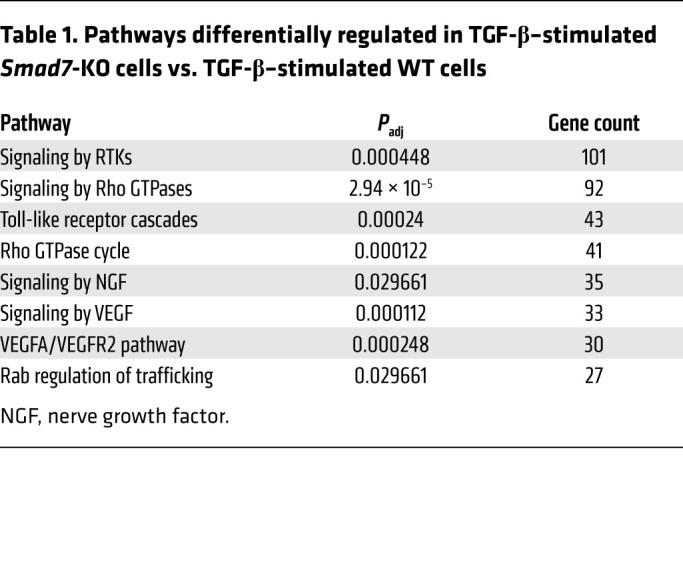
Pathways differentially regulated in TGF-β–stimulated *Smad7*-KO cells vs. TGF-β–stimulated WT cells

## Version 1 12/14/2021

In-Press Preview

## Version 2 02/01/2022

Electronic publication
