# Supplementary material for: Smad7 effects on TGF-β and ErbB2 restrain myofibroblast activation and protect from postinfarction heart failure
Source: J Clin Invest. 2022 Feb 1;132(3):e146926. doi: 10.1172/JCI146926 (PMC8803336; doi:10.1172/JCI146926)

ONLINE SUPPLEMENT

Smad7 effects on TGF-β and Erbb2 restrain myofibroblast activation, and protect from post-infarction heart failure

Claudio Humeres[1], Arti V Shinde[1], Anis Hanna[1], Linda Alex[1], Silvia C Hernández[1], Ruoshui Li[1], Bijun Chen[1], Simon J Conway[2], and Nikolaos G Frangogiannis[1]

SUPPLEMENTAL METHODS

Generation of mice with myofibroblast-specific loss of Smad7: We generated mice with loss of Smad7 in activated myofibroblasts (MFS7KO) by breeding Smad7 fl/fl mice, in which the promoter region and exon 1 are flanked by loxp sites (1) (Jackson labs, stock No: 017008) with a Periostin-Cre transgenic mouse line, in which Cre recombinase is driven by a 3.9-kb mouse Postn promoter (2, 3). Periostin, which is encoded by Postn, is not expressed in cardiomyocytes, vascular cells, hematopoietic cells or quiescent cardiac fibroblasts (4, 5), but is upregulated in injury site-activated fibroblasts in infarcted and in pressure-overloaded hearts (6).

Mouse model of non-reperfused myocardial infarction: Animal studies were approved by the Institutional Animal Care and Use Committee at Albert Einstein College of Medicine and conform to the Guide for the Care and Use of Laboratory Animals published by the National Institutes of Health. A mouse model of non-reperfused myocardial infarction was used, as previously described by our group (7). Female and male mice, 4–6 months of age, were anesthetized using inhaled isoflurane (4% for induction, 2% for maintenance). 27 Smad7 fl/fl mice, and 46 MFS7KO mice underwent in vivo experiments. For analgesia, buprenorphine (0.05–0.2 mg/kg s.c) was administered at the time of surgery and q12h thereafter for 2 days. Additional doses of analgesics were given if the animals appeared to be experiencing pain (based on criteria such as immobility and failure to eat). At the end of the experiment, euthanasia was performed using 2% inhaled isoflurane followed by cervical dislocation. The left anterior descending coronary artery was occluded for 7 days or 28 days. Sham animals were prepared identically without undergoing coronary occlusion. Dead mice hearts following infarction were collected to determine the cause of death by histological analysis.

To assess cardiac function and remodeling following myocardial infarction, animals underwent echocardiographic analysis at baseline and after 7 and 28 days of permanent coronary occlusion. Early euthanasia (2% inhaled isoflurane followed by cervical dislocation) was performed with the following criteria, indicating suffering of the animal: weight loss>20%, vocalization, dehiscent wound, hypothermia, clinical signs of heart failure (cyanosis, dyspnea, tachypnea), lack of movement, hunched back, ruffled coat, lack of food or water ingestion. At the end of the experiment, the animals were sacrificed, and the hearts and lungs were excised and

weighted and fixed in zinc-formalin (Anatech Ltd., Fisher Scientific) and embedded in paraffin for histological studies (control sham group n=8; 7-day permanent occlusion (PO) group: Smad7 fl/fl n= 9, MFS7KO n= 8; 28-day PO Smad7 fl/fl n= 16, MFS7KO n= 20). The surgical protocols were performed by an investigator blinded to the genotype of the animals.

Echocardiography: Echocardiographic studies were performed before instrumentation, 7 and 28 days after coronary occlusion using the Vevo 2100 system (VisualSonics. Toronto ON), as previously described (8). Long-axis M-mode and short-axis M-mode were used for measurement of systolic and diastolic ventricular diameter and wall thickness. The left ventricular end-diastolic diameter (LVEDD), left ventricular end-systolic diameter (LVESD), left ventricular end-diastolic volume (LVEDV), and left ventricular end-systolic volume (LVESV) were measured as indicators of dilative remodeling. Left ventricular mass (LV mass) was measured as an indicator of hypertrophic remodeling. Ejection fraction (LVEF = [LVEDV – LVESV] ×100/LVEDV) were calculated for assessment of systolic function (Smad7 fl/fl, n=29; MFS7KO n=39). Doppler echocardiography and tissue Doppler imaging were acquired from the apical four-chamber view to assess LV filling and diastolic function. Transmitral LV inflow velocities were measured by pulsed-wave Doppler. Peak early E wave (E) and late A wave (A) filling velocities and the E-to-A ratio (E/A) were measured. TDI was obtained by placing a 1.0-mm sample volume at the medial annulus of the mitral valve. Analysis was performed for the early (E′) and late (A’) diastolic velocity. The mitral inflow E velocity-to-tissue Doppler E′ wave velocity ratio (E/E′) and tissue Doppler early e′ velocity-to-tissue Doppler late a′ velocity ratio were calculated to assess diastolic function. All Doppler spectra were recorded for three to five cardiac cycles at a sweep speed of 100 mm/s. The color Doppler preset was at a Nyquist limit of 0.44 m/s. The echocardiographic offline analysis was performed by a sonographer blinded to the study group.

Immunohistochemistry and histology: For histopathological analysis, murine hearts were fixed in zinc-formalin and embedded in paraffin. Infarcted hearts were sectioned from base to apex at 250-μm intervals, thus reconstructing the whole heart, as previously described (8). Ten sections (5 μm thick) were cut at each interval, corresponding to an additional 50-μm segment. To determine the final infarct size, the first section from each

interval was stained with haematoxylin (Sigma-Aldrich, #MHS128-4L) and eosin (Sigma-Aldrich, #HT1102128) (H&E). Picrosirius red (Abcam, #ab246832) staining was used to label the collagen-based scar and collagen content was quantitatively assessed in the infarct region, papillary muscle and remote remodeling myocardium at 7- and 28-day post infarction using ImagePro software. Microvessels were identified in the infarcted and remote remodeling myocardium by using CD31 (Abcam, #ab28364) immunohistochemistry to label endothelial cells. Quantitative analysis was performed by counting the number of vessels. At least 10 different fields from 3 non-adjacent stained sections per mouse at 3 different levels (basal, mid-myocardial, and apical levels) were scanned and analyzed per heart sample (7 days PO: Smad7 fl/fl n= 10; MFS7KO n=9. 28 days PO: Smad7 fl/fl n= 18; MFS7KO n=21).

To determine whether cardiac rupture was the cause of death in mice undergoing myocardial infarction, a subpopulation of 40 mice undergoing 28-day permanent coronary occlusion protocols (19 Smad7 fl/fl, 21 MFS7KO) was followed. Systematic histological analysis of the dead mice hearts following infarction (Smad7 fl/fl n=2; MFS7KO n=9) was performed, examining myocardial sections from base to apex at 250-µm intervals for histological evidence of rupture sites, as previously described (9). To assess the size of acute infarcts the triphenyltetrazolium chloride (TTC) (Sigma-Aldrich, #T8877) staining method was used (10). Smad7 fl/fl (n=8) and MFS7KO (n=8) underwent permanent coronary occlusion protocols, and the heart was harvested 48 hours after infarction. Heart was sectioned from based to apex into 1mm sections. The sections were stained with 1.5% TTC. After TTC staining the viable myocardium is brick red, whereas the infarct appears pale white. All sections were imaged. The area of infarction was assessed planimetrically using Image Pro software. The total volume of the infarct area was calculated and expressed as a percentage of the total volume of the ventricle. Infarct size was compared between Smad7 fl/fl and MFS7KO mice.

To assess Smad7 expression in the infarcted heart, histological sections were stained immunohistochemically with a rabbit anti-mouse Smad7 antibody (Thermofisher, dilution 1:50 #42-0400). Staining was performed with the use of a peroxidase-based technique with the Vectastain ELITE rabbit kit (Vector Laboratories Inc., #PK-6100). Sections were pretreated with a solution of 3% hydrogen peroxide (Sigma-Aldrich, #H1009) to inhibit

endogenous peroxidase activity and incubated with 2% bovine serum albumin (Sigma-Aldrich, #A8531) to block nonspecific protein binding. Peroxidase activity was detected with diaminobenzidine with nickel. Negative controls were performed with the use of normal rabbit IgG (Abcam, #ab37415). Tissues from lung and spleen were used as positive controls for Smad7 immunohistochemistry. Slides were counterstained with hematoxylin. 10 different fields from three non-adjacent stained sections per mouse at in the infarct region and remote remodeling myocardium were scanned and analyzed per heart sample (Sham Control Mice n=6; 7 days PO n=9; 28 days PO n= 14).

Quantitative Morphometry: Morphometric parameters were quantitatively assessed using Zen 2.6 Pro software (Zeiss Microscopy, White Plains, NY). As mentioned before, the entire heart was cross-sectioned from base to apex at 250-µm intervals, with ten serial 5-µm sections obtained at each interval. Volume of the infarct was calculated as the surface area at each interval multiplied by the interval height (300-µm partition). Similarly, noninfarcted volume was calculated by multiplying the surface of the noninfarcted area at each interval by its height (300-µm partition). The total volume of the infarcted and noninfarcted myocardium was calculated as the sum of the volume of each interval. Scar size was measured by dividing the volume of the infarct to the total volume of the left ventricle (left ventricular volume = infarct volume + volume of noninfarcted myocardium) in each interval and was expressed as a percentage (7 days PO Smad7 fl/fl, n= 9; MFS7KO n= 8; 28 days PO Smad7 fl/fl, n=16; MFS7KO n= 11).

Immunofluorescence:

In order to systematically characterize the time course of Smad7 expression in infarct myofibroblasts, Smad7 (Thermofisher, #42-0400 dilution 1:50) and α-SMA staining (Sigma-Aldrich, #F3777 dilution 1:100) was performed on infarcted WT hearts at baseline, and after 7 and 28 days post-infarctions (Baseline n= 8; 7 days PO n= 9; Smad7 fl/fl, n=14). Sections were deparaffinized, and heat-mediated antigen retrieval was performed for 30 min during incubation of the slides in citrate buffer, pH 6.0 (Sigma-Aldrich, #C9999). Sections were then blocked with 10% serum for 1 h and then incubated overnight at 4°C with Smad7 and α-SMA primary antibodies. Sections were then washed and incubated with fluorescently labeled secondary antibodies for 1 h at room temperature.

Autofluorescence quenching was performed using TrueBlack Lipofuscin Autofluorescence Quencher (Biotium, #23007). Slides were mounted using Fluoro-Gel II with DAPI (EMS, #17985-50). Sections were scanned using Zen 2.6 Pro software and the Zeiss Imager M2 microscope Quantitative analysis was performed by counting the number of α-SMA positive cells (total number of myofibroblasts) and dual positive α-SMA+/Smad7+ (number of myofibroblasts expressing Smad7) in 10 random fields from 2 non-adjacent stained sections per mouse at 4 different levels (remote remodeling, basal, mid-myocardial and apical levels). Cell density was expressed as cells/mm^2.

In order to systematically characterize the expression of Smad7 in fibroblasts vs myofibroblasts, triple fluorescent staining experiments were carried out in infarcted PDGFRα-GFP fibroblast reporter mice (11) (Jackson labs, stock No: 007669), combining GFP staining (Abcam, #ab5450 diltuon 1:100, to identify all PDGFRα+ fibroblasts), α-SMA staining (to label myofibroblasts) and Smad7 staining (12, 13). Infarcted PDGFRα-GFP reporter mouse hearts were studied at baseline, and 1, 3, 7, 14 and 28 days after myocardial infarction (Baseline n= 6; 1 days PO n= 5; 3 days PO n=6; 7 days PO n= 6; 14 days PO n=6; 28 days PO n= 6). After secondary antibody incubations, slides were washed and mounted using Fluoro-Gel without DAPI. 10 random fields from 2 non-adjacent stained sections per mouse were scanned at each timepoint. Quantification of Smad7-expressing myofibroblasts was performed by counting the number of nuclei expressing PDGFRα that marked cells positive for Smad7 and α-SMA. Matrifibrocytes were identified using immunofluorescence with the anti-COMP monoclonal antibody (Abcam, #ab231977 dilution 1:50).

Alternative validation of Smad7 expression in activated fibroblasts was carried out by Smad7 immunofluorescent staining experiments on infarcted Periostin-Cre EYFP reporter mice. These mice were generated by breeding Periostin-Cre transgenic mice with ROSA26$^{EYFP-/-}$ (Jackson labs, stock No: 006148) reporter mice. The Postn-Cre;ROSA26^{EYFP} mice will effectively have EYFP labeling only in periostin-expressing cells and their progeny. Infarcted Periostin-EYFP reporter mice hearts at baseline, 7 and 28 days timepoints (Baseline n= 6; 7 days PO n=6; 28 days PO n= 4) were stained with Smad7, and GFP (which labels Periostin-expressing cells). After

secondary antibody incubations, slides were washed and mounted using Fluoro-Gel with DAPI. Quantification was done by counting the number of Periostin-GFP cells that also expressed Smad7.

The Smad7 immunofluorescence protocol was used to validate the myofibroblast-specific loss of Smad7 in MFS7KO. Dual immunofluorescence staining of Smad7 and α-SMA was performed on Smad7 fl/fl and MFS7KO hearts at 7 days post infarction (timepoint of peak expression of both proteins). Representative images showing the loss of Smad7 exclusively in myofibroblasts (α-SMA+/Smad7- cells) in the MFS7KO infarcted hearts, when compared to control Smad7 fl/fl hearts; were obtained by scanning 10 random fields from 2 non-adjacent stained sections per mouse at both remote remodeling and infarcted areas. Unaltered Smad7 expression in non-myofibroblasts (α-SMA-/Smad7+ cells) was also shown in both Smad7 fl/fl and MFS7KO mice as representation of the specificity of the myofibroblast-specific knockout strategy.

For experiments assessing myofibroblast turnover, α-SMA was combined with anti-Ki67 (Thermo Fisher, #14-5698-80 dilution 1:200) and DAPI or TUNEL (Sigma-Aldrich, # 12156792910 dilution 1:200) and DAPI staining to identify proliferating or apoptotic α-SMA myofibroblasts respectively. Quantitative analysis was performed by counting the number of DAPI nuclei that were positive for α-SMA and Ki67 (number of proliferating myofibroblasts) and the number of α-SMA+/TUNEL+ cells (number of apoptotic myofibroblasts) in 10 random fields from 2 non-adjacent stained sections per mouse at infarct and border areas. (Smad7 fl/fl n= 9; MFS7KO n=8).

Activation of Erbb2 in infarct myofibroblasts was compared between Smad7 fl/fl and MFS7KO infarcts using p-Erbb2 and a-SMA dual fluorescence. An anti p-Erbb2 antibody (Abcam Alexa Fluor 488 Anti-ErbB2 / HER2 phospho Y877 #ab237060 dilution 1:100) was used. The number of α-SMA+/pErbb2+ cells was counted in 10 random fields from 2 non-adjacent stained sections per mouse in the infarct zone (Smad7 fl/fl n= 9; MFS7KO n=8).

Cardiac Fibroblast Isolation: Fibroblasts were isolated from 12-week-old mouse (C57/BL6J) hearts with protocols used in our laboratory (14). Briefly, hearts were dissected free of vessels and atria and quickly minced and digested by shaking the tissue fragments (1 hour at 37°C water bath) in 100 U/mL collagenase CLS2

(Worthington Biochemical Lakewood, NJ) and DNAse (40 ug/mL, Stemcell Technologies) prepared in KHB-buffer (Thermo Fisher, #501907929). Digestion with collagenase buffer continued until no visible tissue fragments were left. Digested tissue was filtered through a 70 μm cell strainer followed by low-speed centrifugation (50 g, 2 min) to remove cardiomyocytes, cell clumps and undigested tissue. Next, the supernatants were passed through a 40 μm cell strainer, centrifuged at 450 g for 4 min and washed with PBS (Thermo Fisher, #10010-023). The isolated cell suspensions from each round were pelleted and washed, then resuspended in Dulbecco's modified Eagles medium (DMEM/F12, Thermo Fisher, #11-330-032) containing 10% fetal bovine serum (FBS), and antibiotics (PenG 100U/ml, Streptomycin sulfate 100 ug/ml, Amphotericin B 0.25 ug/ml, GIBCO, Grand Island, NY). Cells were plated on a T75 tissue-culture flask (Corning Corp, Acton, Mass) and incubated overnight at 37°C, 5% CO_2. After overnight incubation, nonadherent cells were removed, and adherent cells were cultivated. On reaching confluence, cells were detached with trypsin/EDTA, split in a 1:2 or 1:4 ratio, and recultured (passage 1). Characteristic fibroblast morphology was determined visually under a light microscope. Fibroblasts were used up to passage 2 and serum-starved overnight for stimulation experiments.

Fluorescence-activated cell sorting (FACS) of cardiac fibroblasts from baseline or infarcted hearts: FACS sorting of CD31-/C45- cell population was performed on Smad7 fl/fl and MFS7KO mice at baseline, or after 21 days of coronary occlusion. Briefly, myocardial tissue was finely minced and suspended in digestion buffer cocktail of collagenase IV (2 mg/mL, Worthington Biochem) and dispase II (1.2 U/mL, Stemcell Technologies) in DPBS. Tissue fragments were then incubated at 37 °C for 15 min with gentle rocking. After incubation, tissue digestion buffer with tissue clusters was triturated by pipetting 10 times using a 10-mL serological pipette. Tissue fragments were again incubated at 37 °C and triturated twice more (45 min of total digestion time). The final trituration was conducted by pipetting 30 × with a p1000 pipette. Cell suspension was filtered through 40 μm cell strainer into 50 mL tubes containing 40 mL DPBS and centrifuged for 20 min at 200 g with centrifuge brakes deactivated to remove cell debris. Cells were then resuspended in 5 mL of RBC lysis buffer (Thermo Fisher, #00-4333-57) and incubate for 5 min at RT. Cell suspension was then centrifuged at 500 g for 5 min at RT and resuspended in Hanks' Balanced Salt Solution (HBSS, Thermo Fisher, #24020-117) containing 2% fetal bovine

serum (FBS). Cells in single cell suspensions were blocked with anti-mouse CD16/CD32 (Thermo Fisher, #14-061-82 dilution 1:40) for 30 min at 4 °C. In order to identify hematopoietic and endothelial cells, the cell suspension was incubated for 1 h at 4 °C with anti-CD45-BV605 (Biolegend, #103139, dilution 1:100) and anti-CD31-APC single bond Cy7 (BioLegend, #102049 dilution 1:40). Cell suspension was washed and labelled with 7-aminoactinomycin D (Invitrogen, #A1310 dilution 1:500) to identify cells with compromised cell membranes. Non-hematopoietic/non-endothelial cells (CD45-/CD31-), which are predominantly fibroblasts (13), (15) were sorted with the FACSAria (BD Biosciences) and processed for RNA isolation. FlowJo software was used for data analyses.

Smad7 knockout in cardiac fibroblasts by Adenovirus-mediated Cre Expression: For Smad7 deletion, mouse cardiac fibroblasts isolated from Smad7 fl/fl mice, were infected with adenovirus expressing Cre-recombinase (Ad-Cre-GFP, Vector Biolabs #1700), in order to knockout LoxP flanked Smad7 gene. Empty adenovirus (Ad-GFP, Vector Biolabs #1060) was used as a transfection control. Ad-Cre-GFP and Ad-GFP were propagated in HEK293 cells (ATCC, #CRL-1573) and titrated using a plaque quantification assay kit (AdEasy Viral Kit, Agilent Technologies #972500) according to manufacturer instructions. Viral Stock titrations were: Ad-Cre-GFP, 1.42 x 10^{12} IFU/mL; Ad-GFP, 8.54 x 10^{11} IFU/mL.

In a pilot experiment we tested the effectiveness of 4 different Multiplicity of Infection (MOI) of the adenovirus: 2, 20, 100 and 200 MOI in reducing Smad7 RNA and protein levels in unstimulated cardiac fibroblasts isolated from Smad7 fl/fl mice. Because of the high efficiency and low toxicity, we used 100 MOI transfection for 24 hours for all following in vitro Smad7 knockout experiments. Smad7 loss was confirmed at both mRNA and protein levels by qPCR and Western blots experiments comparing Smad7 levels in control fibroblasts (transfected with Ad-GFP) versus Smad7 KO (S7KO) fibroblasts (transfected with Ad-Cre-GFP). After transfection, cells were serum deprived overnight and stimulated according to the different experiments.

Smad7 Overexpression: For Smad7 overexpression experiments, mouse cardiac fibroblasts at passage 2 were transfected for 24 hours with 10 ug of a plasmid containing Mouse tagged Smad7 cDNA clone (Smad7 Myc-DDK-tagged, Origene Technologies #MR226590) or transfected with a control entry vector (pCMV6-Entry Myc-

DDK Tag, Origene Technologies #PS100001) using Lipofectamine®3000 Reagent (ThermoFisher Scientific, #L3000-015). After transfection, cells were serum deprived overnight and stimulated according to the different experiments. Efficiency of Smad7 overexpression was confirmed at both mRNA and protein levels by qPCR and Western blots experiments comparing Smad7 levels in control fibroblasts (transfected with control vector) versus Smad7 overexpression (S7OE) fibroblasts (transfection with Smad7 cDNA plasmid).

Experiments in cardiac fibroblasts-populated collagen pads: In order to study the effects of TGF-βs ligands on Smad7 expression, cardiac fibroblasts were cultured in collaged pads as previously described (7). Collagen matrix was prepared by diluting a stock solution of 3.0 mg/ml rat collagen I (Thermo Fisher, #A1048301) with 2x DMEM and distilled water for a final concentration of 1 mg/ml collagen. Cell suspensions were mixed with collagen solution to achieve the final 3 x 10^5 cells/ml concentration. Subsequently, 500 µl of this suspension was aliquoted to a 24-well culture plate (BD Falcon) and allowed to polymerize at 37ºC for 30 min. Following polymerization, the pads were released from the wells, transferred to 6-well culture plate (BD Falcon, San Jose, CA) and cultured in 0% FBS DMEM/F12 or in presence of TGF-β1 (10ng/ml, #7666-MB-005/CF), TGF-β2 (10ng/ml, #7346-B2-005/CF), TGF–β3 (10ng/ml, #5034-R3-050), activin A (50ng/ml, #338-AC-010/CF), activin B (50ng/ml, #8260-AB-010/CF), myostatin (100ng/ml, #788-G8-010/CF), or GDF-11 (10ng/ml, #1958-GD-010/CF) (all from R&D systems) for 30 min. Pads were used for RNA isolation, or fixed in zinc-formalin (Anatech Ltd., Fisher Scientific) and horizontally embedded in paraffin along the thin side of the pad. Pads were sectioned (5 µm thick) and used for immunofluorescence experiments. For experiments assessing collagen synthesis/denaturation in cardiac fibroblasts-populated collagen pads, we performed dual fluorescence with an anti-collagen type III antibody (Abcam #ab7778 dilution 1:100) to assess de novo collagen synthesis, and with a Collagen Hybridizing fluorescent Peptide 5-FAM conjugate (3Helix, Inc #F-CHP) that specifically binds to denatured unfolded collagen (16),(17). Sections from infarcted and control hearts were deparaffinized and heat-mediated antigen retrieval was avoided to avoid denaturation of the native collagen in the tissue. Sections were then blocked with 5% BSA for 1 h. 20 µM dilution of F-CHP was heated for 5 min at 80 °C, then quenched on ice for 1 min. Sections were incubated with F-CHP at 4 °C and Anti collagen III primary antibody overnight.

Sections were then washed and incubated with fluorescent labeled secondary antibody for 1h at room temperature. Autofluorescence quenching was performed using TrueBlack Lipofuscin Autofluorescence Quencher (Biotium, #23007). Slides were mounted using Fluoro-Gel II with DAPI and scanned using Zen 2.6 Pro-software.

Smad3 in vitro and in vivo knockdown experiments: For experiments assessing the role of Smad3 in Smad7 expression in cardiac fibroblasts in vitro, cardiac fibroblasts at passage 2 were seeded at 80% confluence in complete medium and were transfected with either Smad3 siRNA (Mission Sigma, #NM_016769, Millipore-Sigma) or non-silencing control siRNA (Thermo Fisher, #AM4611) using Lipofectamine® 3000 Reagent for 48 hours. Smad3 knockout fibroblasts (S3KO) and control cardiac fibroblasts were harvested using TrypLE™ (Thermo Fisher, #12-604-021), populated in collagen pads and treated with/without TGF-β1for 30 min. RNA was extracted and used for qPCR assessing Smad7 expression (n=5 for each condition). In order to evaluate Smad3 role in Smad7 expression on cardiac fibroblasts in vivo, isolation of cardiac fibroblasts from non-infarcted and infarcted areas of the heart of control and fibroblast-specific Smad3 knockout mice (Postn-Cre;Smad3 fl/fl mice) was performed as previously described by our group (18). RNA was extracted and evaluated for Smad7 expression by qPCR (n=5 for each condition).

Evaluation of Smad7 role in Receptor Tyrosine kinase Activation: For experiments assessing the role of Smad7 on Receptor Tyrosine Kinase (RTK) activation, control and S7KO cardiac fibroblasts, (passage 2) were cultured in plates for 30 or 120 min the presence or absence of TGF-β1 (10ng/ml), Amphiregulin (1 ng/mL, #989-AR-100/CF) or HB-EGF (10 ng/ml, #MAB8239-100) (All from R&D Systems). Cell lysates were used for protein extraction and used for western blot assessing phospho-RTK levels. In order to study the effect of RTK on cardiac fibroblasts on modulating fibroblast gene expression, control and S7KO cardiac fibroblasts were pretreated with the Erbb1/Erbb2 dual inhibitor Lapatinib (Sigma-Aldrich, #231277-92-2) (5 µM) for 1 hour, followed by treatment with or without TGF-β1 (10ng/ml), Amphiregulin (1 ng/mL) for 4 hours. For experiments assessing the TGF-β independence of the effects of Smad7 on Erbb2, passage 2 control and S7KO cardiac fibroblasts were incubated for 2 hours with the ALK 4/5/7 inhibitor; SB431542 (10 µM, Sigma-Aldrich #S4317) before protein harvest.

Protein extraction and Western blotting: Protein from whole hearts or cardiac fibroblasts were extracted in T-PER Tissue Protein Extraction Reagent (Thermofisher, #78510) or RIPA lysis buffer (Thermo Fisher, #89900), respectively; including 0.5M EDTA, 1X Protease and Phosphatase Inhibitors (Thermo Fisher, #78440). Equal amount of protein from each experimental group was fractionated by 10% Mini-PROTEAN® TGX™ Gel (Bio-Rad Laboratories, #456-1036) and transferred onto a PVDF membrane (Bio-Rad Laboratories, #1620177). Membranes were incubated overnight with primary antibodies at 4C followed by the application of appropriate horse radish peroxidase (HRP)-conjugated secondary antibodies for 2 hours. Proteins were detected using Pierce™ ECL Western blot detection reagents (Thermofisher, #32106) and imaged by the ChemiDoc™ MP System (Bio-Rad Laboratories). Bands were densitometrically quantified by Image Lab 3.0 software (Bio-Rad Laboratories) and normalized to appropriate loading controls.

The following primary antibodies from Cell Signaling were used: p-Smad3 (1:1000, #9520), p-Smad2 (1:1000, #3108), p-Akt (1:1000, #9271), p-Erk (1:1000, #4376), p-EGFR (1:500, #3777S), p-Erbb2 (1:1000, #2243), p-Erbb4 (1:500, #4757), Smad3 (1:1000, #9513), TGFBR1 (1:1000 #3712), Smad2 (1:1000, #5339), Akt (1:1000, #9272), Erk (1:1000, #4695), EGFR (1:1000 #4267), Erbb2 (1:1000 #2165) and Erbb4 (1:1000 #4757). pTGFBR1 (1:500 #PA540298) was obtained from Invitrogen. pTGFBR2 (1:500, ab111564) and TGFBR2 (1:500, ab269279) were obtained from Abcam. Smad7 (1:250, #sc-101152) and GAPDH (1:1000 #sc-25778) were obtained from Santa Cruz.

RNA extraction, PCR arrays and qPCR: Total RNA was extracted from cells and mouse hearts using TRIzol reagent (Qiagen, #79306). A total of 500 ng of RNA was transcribed into cDNA using the RT2 first-strand kit (Qiagen, #330404). Quantitative PCR was then performed using the RT^2 Profiler mouse Extracellular matrix and adhesion molecules PCR array (#PAMM-013ZE-4) from Qiagen according to the manufacturer's protocol. The same thermal profile conditions were used for all primers sets: 95°C for 10 minutes, 40 cycles at 95°C for 15 seconds and 60°C for 1 minute. The data obtained were exported to the SABiosciences PCR array web-based template where it was analyzed using the ΔΔCt method. The housekeeping gene GAPDH was used as internal control. The

qPCR procedure was repeated three times in independent runs; gene expression levels were calculated using the $2^{-\Delta CT}$ method. The sequences of the primers used in this work are shown in Supplemental Table 1.

Phospho-receptor tyrosine kinase Array: The relative level of tyrosine phosphorylation of 39 different receptor tyrosine kinases was determined in control and S7KO cardiac fibroblasts in absence or presence of TGF-β1 (2 h stimulation, 10 ng/mL) using the Proteome Profiler Mouse Phospho-RTK Array Kit (R&D Systems, #ARY014) according to manufacturer's instructions. Briefly, 300 ug of cell lysis were incubated overnight with a nitrocellulose membrane containing capture and control phospho-RTK antibodies. Following incubation, excess protein is washed and pan anti-phospho-tyrosine antibody conjugated to horseradish peroxidase (HRP) is then used to detect phosphorylated tyrosines on activated receptors by chemiluminescence. Relative levels of phosphorylation were quantified by measuring the pixel intensity of the signals developed by incubating samples with the array membrane in Pierce™ ECL Western blot detection reagents (Thermo Fisher, #32106) and imaged by the ChemiDoc™ MP System (Bio-Rad Laboratories). Specific Western blots were performed to confirm the results obtained in the Phospho-RTK array.

Erbb2-Smad7 coimmunoprecipitation Assay: Erbb2-Smad7 coimmunoprecipitation was evaluated using Dynabeads Co-Immunoprecipitation Kit (Invitrogen, #14321D) according to the protocol provided by the manufacturer. Briefly, cardiac fibroblasts treated in presence or absence of TGF-β1 (10 ng/mL) or Amphiregulin (1 ng/mL) were harvested and lysed in extraction buffer containing 0.5% Triton X-100 (Millipore Sigma #112298) and protease/phosphatase inhibitor cocktail. 20 mg of Epoxy M-270 magnetic beads prepared in 2 mL of buffer were coupled overnight (4°C) with Erbb2 antibody (Cell Signaling, #2165 dilution 1:100) to form Erbb2-magnetic conjugated beads. Coupling of Epoxy M-270 magnetic beads to Rabbit IgG antibody was used as the immunoprecipitation control.

Erbb2-beads and IgG-beads were incubated with 100 mg of the protein lysate previously prepared for 45 minutes with rotation at 4°C, to allow the formation of Erbb2 beads-protein complex (and control IgG beads-protein complex). The complexes were washed 4 times with extraction buffer while on a magnetic bar to remove unbound protein and further eluted in SDS sample loading buffer. Eluted proteins containing the purified Erbb2-bound or

IgG control-bound coimmunoprecipitated proteins were assessed for Smad7 presence by western blot. Erbb2 western blots were also performed to confirm the positive presence of the capture Erbb2 protein in the coimmunoprecipitated complexes and its absence in the IgG CoIP complex. Whole cell lysate/input sample was used as a control of the immunoprecipitation enrichment.

Library preparation for transcriptome sequencing: RNA isolated from unstimulated control fibroblast (CWT), TGF-β1 treated control fibroblasts (TWT), Unstimulated Smad7 KO fibroblasts (CS7) and TGF-β1 treated Smad7 KO fibroblasts (TS7), run in three replicates each, were sent to Novogene (Sacramento, California) to construct a total of twelve cDNA libraries by using NEBNext® Ultra™ RNA Library Prep Kit for Illumina® (NEB, Ipswich, MA, USA).

In brief, the process of library construction consisted of i) mRNA purification and enrichment from total RNA using oligo(dt)-attached magnetic beads, ii) fragmentation of purified mRNA using divalent cations exposed to elevated temperatures in NEBNext First Strand Synthesis Reaction Buffer, iii) double stranded cDNA synthesis, using Rnase H- reverse transcriptase (first strand) and DNA polymerase I, dNTP and RNase H (second strand); iv) terminal cDNA ends reparation by exonucleases/polymerases; and poly adenylation of the 3' ends of the DNA fragments, v) Sequencing adaptors ligation and finally vi) cDNA fragments size selection (150-200 bp in length) which underwent PCR. PCR was performed using Phusion High-Fidelity DNA Polymerase, universal PCR primers and Index (X) Primer. PCR products were purified (AMPure XP system), and the library concentration (>2 nM) and quality were assessed using a Bioanalyzer 2100 system (Agilent, Santa Clara, CA, USA).

Quality analysis, mapping and assembly: The library preparations were sequenced on Illumina Hiseq 2000 devices, generating 100 bp paired end reads. Adapter, poly-N and low-quality reads from the raw data were excluded to purify the data analysis. Quality Phred Scores, Q20%, Q30% and GC contents of the clean data were calculated, showing high accuracy of reads (>99%, with 0.02 error rate). Filtered reads were aligned to the C57BL/6J reference genome (December 2011 release of the mouse musculus reference genome (mm10; GRCm38.p6) from Ensembl) using TopHat2 algorithm alignment program v.2.0.9 (19). Mapped reads were assembled using both Scripture (beta2) and Cufflinks v.2.1.1 algorithm (20).

Gene expression, differential expression, enrichment and co-expression analysis: HTSeq software v.0.6.1 (21) was used to count the number of reads mapped to each gene. Read count of fragments per kilobase of transcript sequence per millions base pairs sequenced (FPKM), was used to calculate gene expression level, which considered the effects of both sequencing depth and gene length (20). Readcount obtained from the gene expression analysis was used for differential expression analysis. Cluster differential expression analysis for every gene in the four different cardiac fibroblast conditions was performed using the DESeq2 R software package (v.1.10.1) (22). Genes with an adjusted P-value ≤ 0.05 were considered to be differentially expressed. List of genes were ranked by differential gene expression as log2 (fold change) between each comparison group. Positive values were upregulated genes, whereas negative values were downregulated genes.

Using the log2 fold changes obtained from the differential expression analysis for every gene, gene enrichment analysis (Gene ontology, GO) and pathway (KEGG and Reactome) analysis were implemented using the ClusterProfiler software package (23). Reactome pathways analysis was further utilized given its strict classification associated with databases of reactions and disease-related pathways. Reactome pathways with a corrected P-value ≤ 0.05 were considered to be significantly enriched. Differentially regulated Reactome pathways were ranked by their -log10(adjusted p value) between each comparison group.

RNA-seq data were deposited in GEO database (GSE185767).

Statistics: For all analyses, normal distribution was tested using the Shapiro–Wilk normality test. For comparisons of two groups unpaired two-tailed Student's t test using (when appropriate) Welch's correction for unequal variances was performed. The Mann–Whitney test was used for comparisons between two groups that did not show Gaussian distribution. For comparisons of multiple groups, one-way ANOVA was performed followed by Tukey's multiple comparison test. The Kruskal–Wallis test, followed by Dunn's multiple comparison posttest was used when one or more groups did not show Gaussian distribution. Survival analysis was performed using the Kaplan–Meier method. Mortality was compared using the log rank test. Data are expressed as means ± SEM. Statistical significance was set at $P < 0.05$.

SUPPLEMENTAL TABLES

Supplemental Table 1: Sequence of primers used

Primer	Forward Sequence	Reverse Sequence
Chad	5’- CAA CTC GTT TCG GAC CAT GC -3’	5’- GAT GTC GTT GTG GGA CAG GT -3’
Clip2	5’- TGG AGG CTC GTG TCA CTT TC -3’	5’- CAG GTC CAC TGC AAA CAT GC -3’
Col1a1	5’- CCT CAG GGT ATT GCT GGA CAA C -3’	5’- CAG AAG GAC CTT GTT TGC CAG -3’
Col3a1	5’- GAC CAA AAG GTG ATG CTG GAC AG -3’	5’- CAA GAC CTC GRG CTC CAG TTA G -3’
Col5a1	5’- TGG GAT AGA GAC AGG GCT TTG -3’	5’- AGT CAT TTG GTT CCC TCC CAG -3’
Comp	5’- AGC TCA AGG CTG TCA AGT CC -3’	5’- GGG AGA AGC AGA CAC CC -3’
Fibronectin	5’- CCC TAT CTC TGA TAC CGT TGT CC -3’	5’- TGC CGC AAC TAC TGT GAT TCG G -3’
GAPDH	5'- AAC GAC CCC TTC ATT GAC CT -3’	5’- CAC CAG TAG ACT CCA CGA CA -3’
LOX	5’- TAA CTG CAA ACT GCC ACG TC -3’	5’- GAG GCT CAG CAG ACT GAA CA -3’
MMP1a	5’- CAC AAC AAT CCT CGT TGG AC -3’	5’- TGG TGT CAC ATC ACT CCA GA -3’
MMP12	5’- AAG GGA ACT TGC AGT CGG AG -3’	5’- TGC TGG GAA CCT TCA GCA AA -3’
MMP3	5’- CAT CCC CTG ATG TCC TCG TG-3’	5’- CTTCTTCACGGTTGCAGGGA -3’
PDGFRa	5’- TCA GAG AGA ATC GGC CCC A -3’	5’- AGG ACG AAT TCA GCT GCA CA -3’
Postn	5’- AAA CGG AAG GAC CTG CAA TG -3’	5’- TTG CAG GTG TGT CTT TTT GC -3’
Smad7	5’- CTG CAA CCC CCA TCA CCT TA -3’	5’- CAG CCT GCA GTT GGT TTG AG -3’
TIMP1	5’- GCC TGA ACA CTG TCT ACT T -3’	5’- TTG CTG CTG TCT GAT AGT T -3’
TIMP3	5’- TCC AAA CAC TAC GCC TGC AT -3’	5’- CTG CTT GCT GCC TTT GAC TG -3’
TSP1	5’- GGT AGC TGG AAA TGT GGT GCG T -3’	5’- GCA CCG ATG TTC TCC GTT GTG A -3’
TSP2	5’- TCC TTC TCA TCG GTC ACA CCG -3’	5’- CTG TGT CAA CAC AGC CTG GC -3’
Vimentin	5’- TGA GAT CGC CAC CTA CAG GA -3’	5’- TTG CGC TCC TGA AAA ACT GC -3’

SUPPLEMENTAL FIGURES:

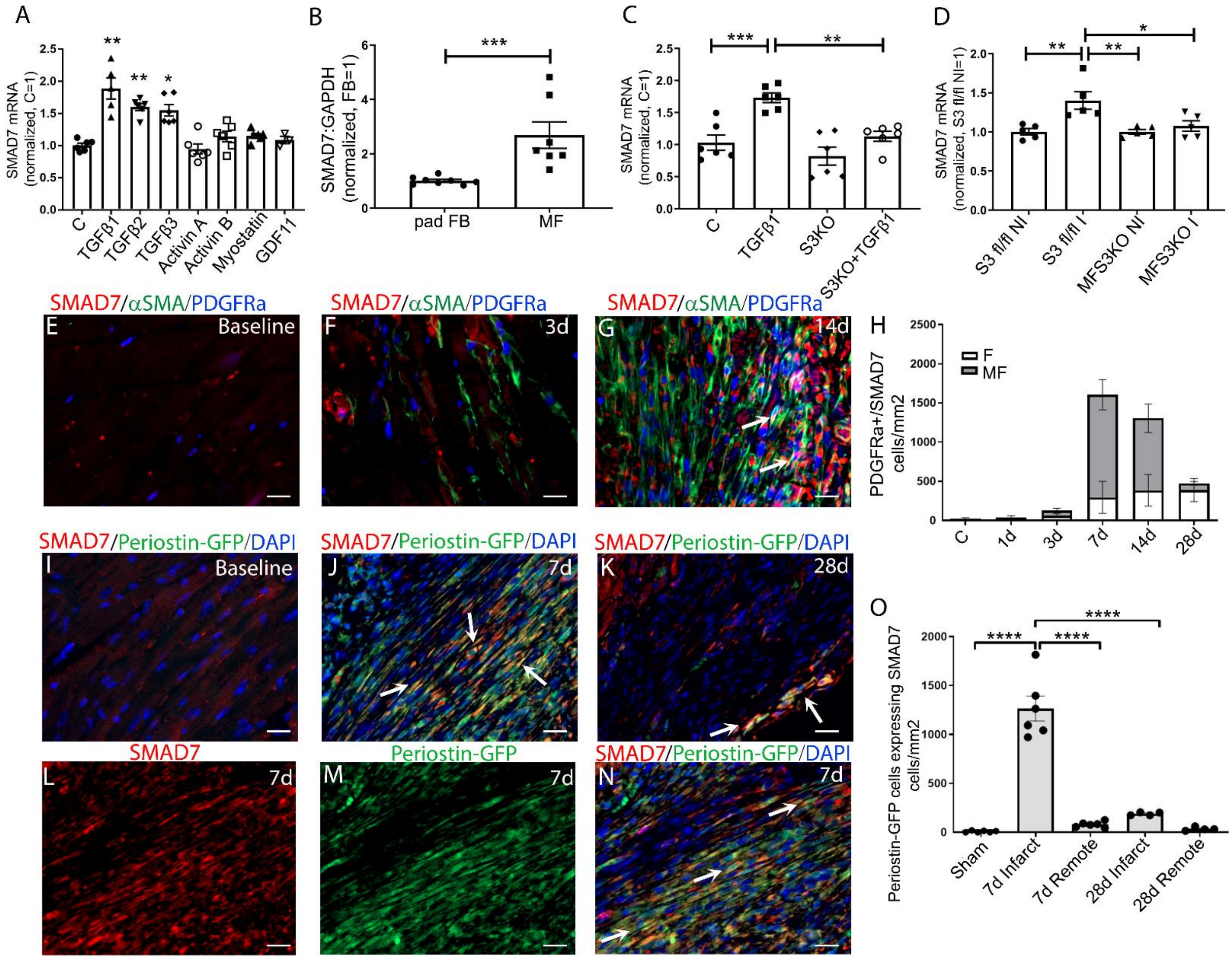

Supplemental Figure 1. Smad7 expression is markedly upregulated in infarct fibroblasts, is associated to myofibroblast conversion, and is dependent on Smad3 signaling. (A) In cardiac fibroblasts populating collagen lattices, all three TGF isoforms (β1, β2 and β3), but not activin-A, myostatin and GDF-11, induce an increase in *Smad7* levels (n=3). **(B)** *Smad7* expression is much higher in cardiac fibroblasts cultured in plates (which exhibit myofibroblast conversion, MF), in comparison to fibroblasts cultured in collagen pads (pad FB; which have low levels of α-SMA) (n=7). **(C-D)** Smad3 mediates Smad7 upregulation. **(C)** Cardiac fibroblasts harvested from Smad3 KO mice (S3KO) have markedly attenuated *Smad7* expression upon stimulation with TGF-β1. **(D)** Infarct fibroblasts harvested from myofibroblast-specific Smad3 KO infarcts (MFS3KO I) have significantly lower *Smad7* levels in comparison to fibroblasts harvested from Smad3 fl/fl infarcts (S3 fl/fl I). n=5; *p<0.05, **p<0.01, ***p<0.001. **(E-G)** Triple immunofluorescence staining for Smad7, α-SMA and PDGFRα-GFP, in PDGFR-GFP fibroblast reporter mice at baseline, and 3d and 14d after infarction. **(H)** The relative contribution of fibroblasts (F) and myofibroblasts (MF) in SMAD7 expression at different timepoints after infarction. **(I-O)** In periostin-Cre;ROSA26^{EYFP} mice, triple immunofluorescence staining combining Smad7, α-SMA and Periostin-GFP was used to identify activated myofibroblasts that express SMAD7 in the infarcted myocardium. Individual **(L-N)** and merged **(I-K)** channels for Smad7, α-SMA and Periostin-GFP fluorescence

shows that Smad7 is mostly expressed by Periostin+ myofibroblasts in the infarct zone (arrows) at 7 days **(O)**. For comparisons between multiple groups (A, C, D and O) one-way ANOVA followed by Tukey's multiple comparison test was performed. For comparisons between two groups (B) unpaired two-tailed Student's t test was performed. (A-D): n = n=5-7; *p<0.05, **p<0.01, ***p<0.001. (H and O): n = 6/group; ****p<0.0001. Scale bar = 20 µm.

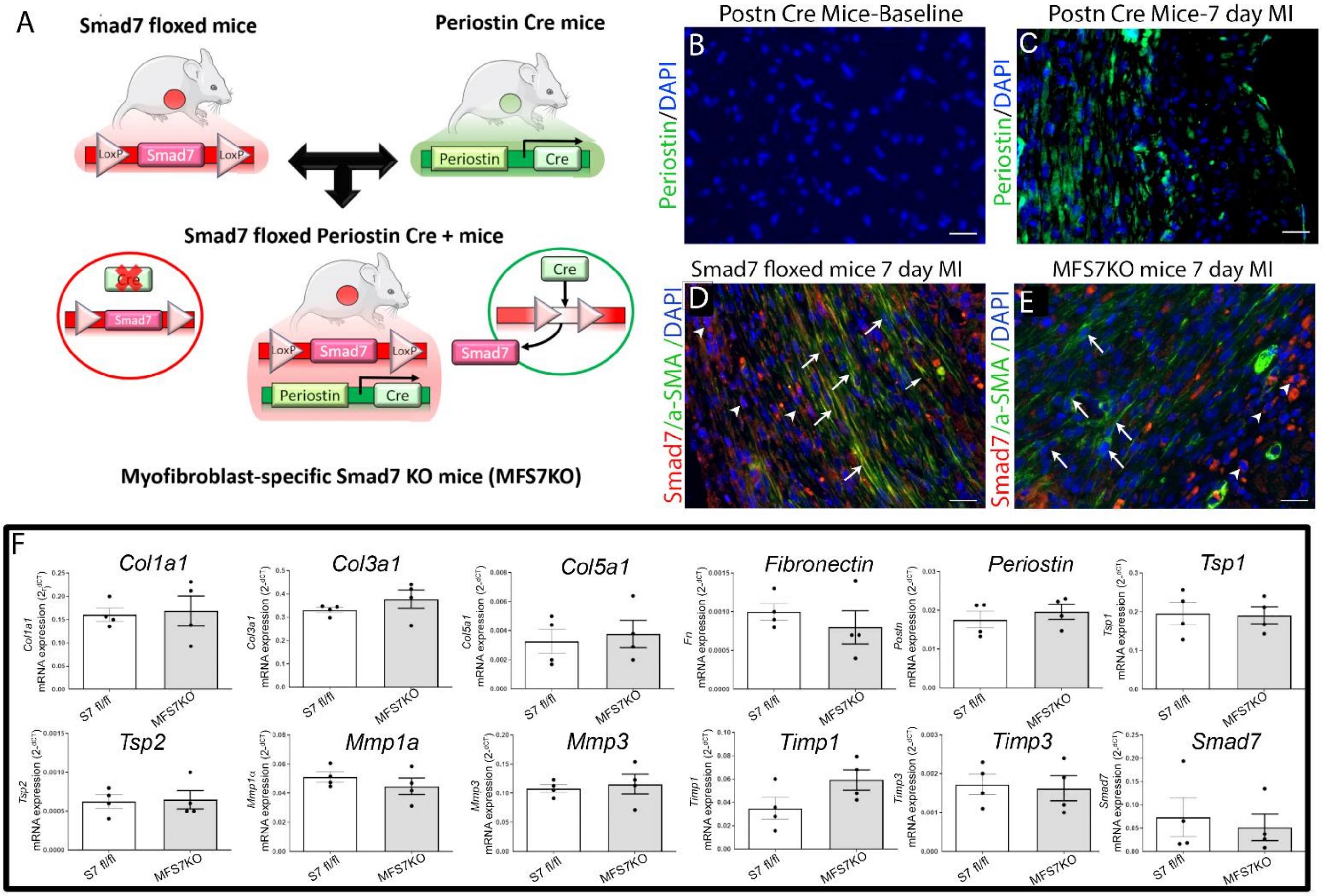

Supplemental Figure 2. Development of myofibroblast-specific Smad7 KO (MFS7KO) mice using the Cre-lox system. (A) Schematic representation of the breeding strategy utilized to generate MFS7KO by Cre-mediated excision of Smad7 in periostin-expressing myofibroblasts. **(B-C)** Representative images show that in Postn-Cre;ROSA26^{EYFP} mice, resident fibroblasts in normal hearts are not labeled. In contrast, infarct myofibroblasts are intensely stained (7-day timepoint). **(D-E)** Representative images validating Smad7-myofibroblast specific knockout. **(D)** Dual Smad7/α-SMA immunofluorescence after 7 days of permanent occlusion shows infarct myofibroblasts (white arrows) co-expressing Smad7 in Smad7 fl/fl animals. Smad7 expression is absent in α-SMA+ infarct myofibroblasts in MFS7KO hearts **(E).** Importantly, Smad7 expression in non-myofibroblasts (α-SMA- cells) is similar in both control Smad7 fl/fl and MFS7KO hearts, validating the specificity of the myofibroblast specific knockout strategy. **(F)** qPCR comparison of profibrotic genes in cardiac fibroblasts from S7fl/fl and MFS7KO control hearts show that there is no difference in fibrosis associated genes such as Collagens, fibronectin and *Mmps*, or in *Smad7* mRNA levels. Comparisons between two groups (F) was performed by unpaired two-tailed Student's t test using Welch's correction for unequal variances. n= 4. Scale bar = 20 µm.

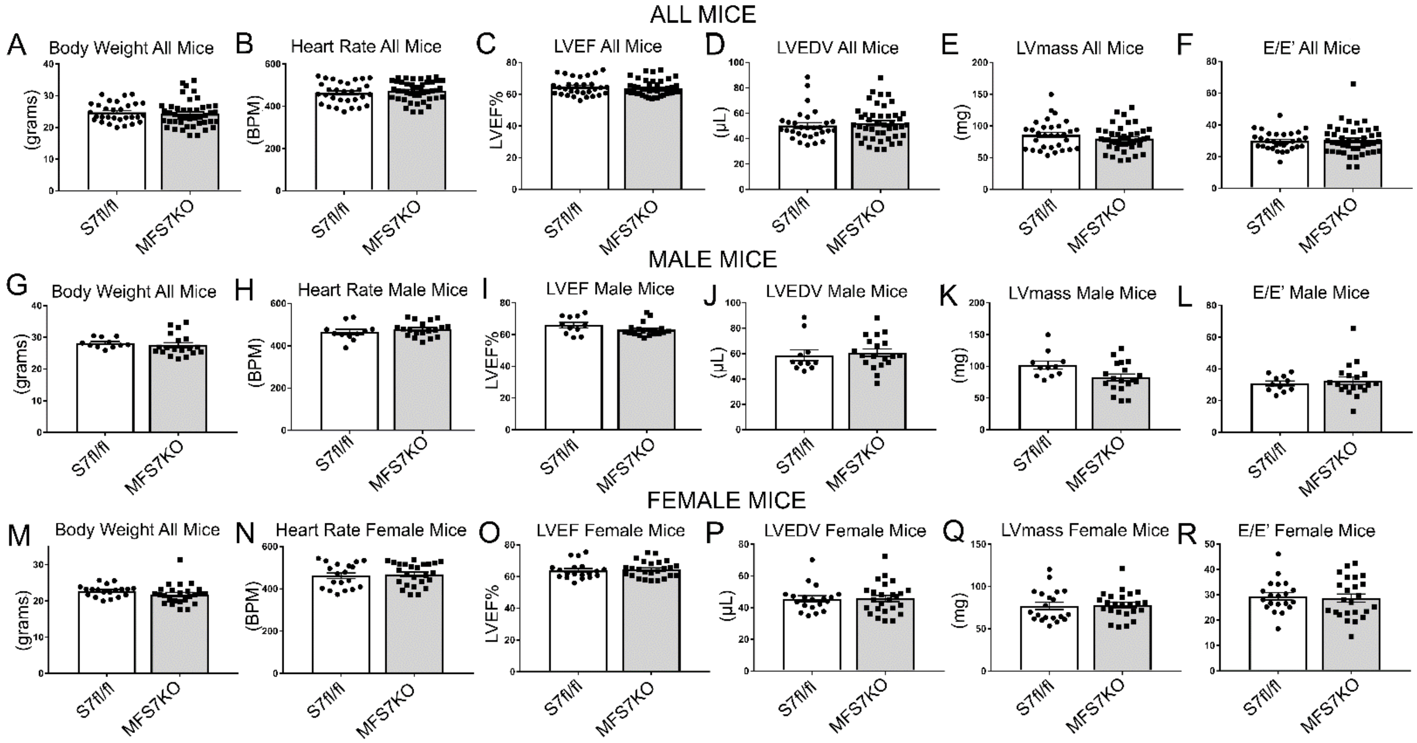

Supplemental Figure 3. Myofibroblast-specific Smad7 knockout mice have no baseline cardiac defects. Body Weight and echocardiographic comparison of Control Smad 7 fl/fl hearts (S7fl/fl) and myofibroblast specific Smad7 KO mice (MFS7KO), showing that there are no baseline differences in weight **(A)**, heart rate **(B)**, left ventricular ejection fraction **(**LVEF**; C)**, left ventricular end-diastolic volume **(**LVEDV**; D)**, left ventricle mass **(E)** and E/E' ratio **(F)**. Male **(G-L)** and female **(M-R)** mice show similar findings. Comparisons between two groups (A-R) was performed by unpaired two-tailed Student's t test. S7fl/fl n=30 (male n= 11, female n= 19), MFS7KO n=44 (male n= 19, female n= 25).

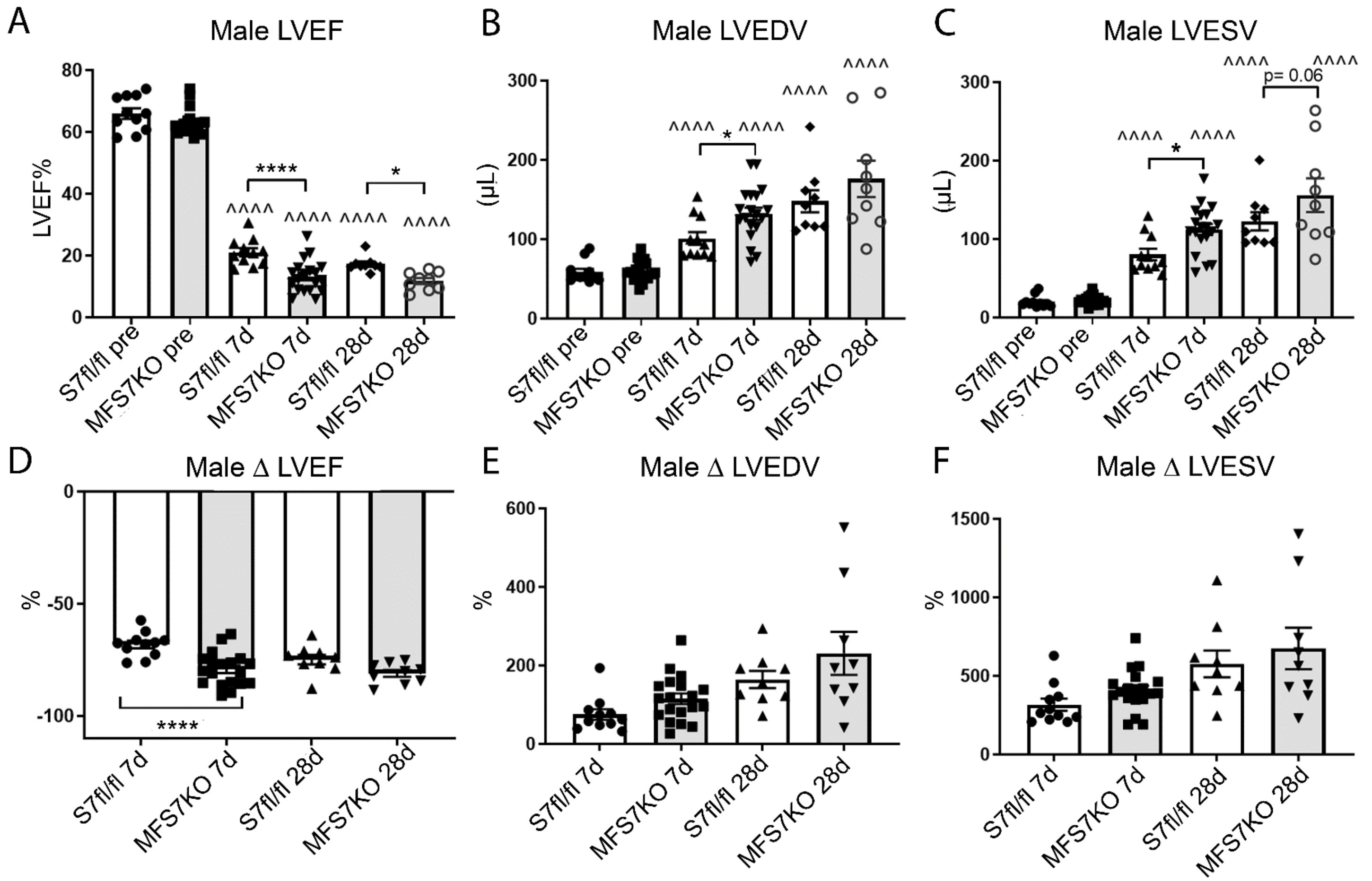

Supplemental Figure 4. Myofibroblast-specific Smad7 loss increases left ventricular dysfunction and accentuates adverse post-infarction remodeling in male mice. Echocardiographic assessment of adverse remodeling after 7-28 days following infarction shows that male MFS7KO mice have **(A, D)** worse systolic dysfunction, evidenced by a lower ejection fraction at both timepoints. **(B-C, E-F)** MFS7KO mice have accentuated dilative ventricular remodeling evidenced by increased left ventricular end-diastolic volume (LVEDV) and left ventricular end-systolic volume (LVESV) at the 7-day timepoint. Statistical comparison (A-F) was performed using one-way ANOVA followed by Tukey's multiple comparison test. Male Smad7 fl/fl n= 11, male MFS7KO n=19; *p<0.05, ****p<0.0001, ^^^^p<0.0001 vs. baseline pre-infarction (pre) condition.

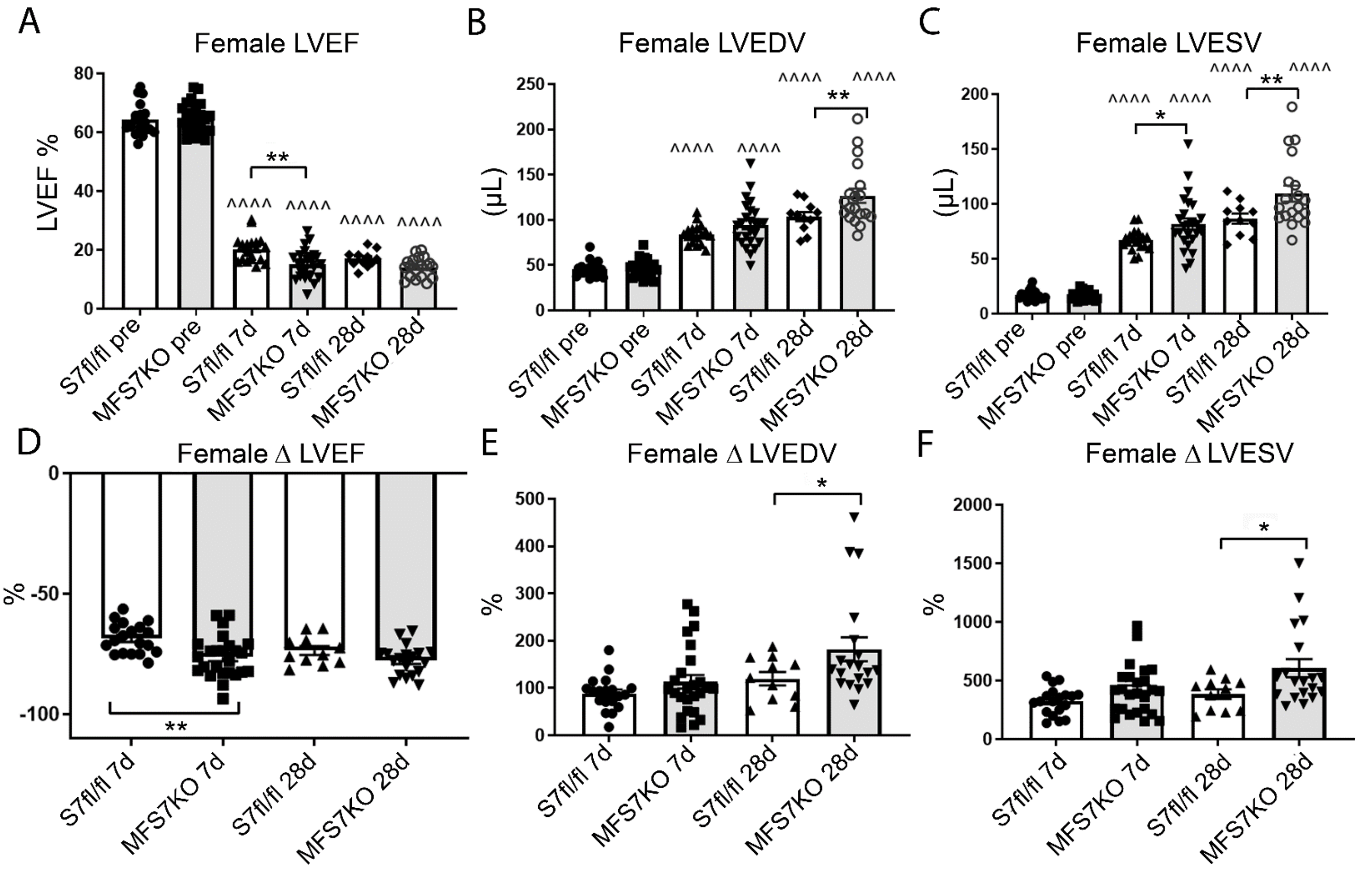

Supplemental Figure 5. Myofibroblast-specific Smad7 loss worsens left ventricular dysfunction and accentuates adverse post-infarction remodeling in female mice. Echocardiographic assessment of adverse remodeling after 7-28 days following infarction shows that female MFS7KO mice have **(A, D)** worse systolic dysfunction demonstrated by a lower ejection fraction at the 7-day timepoint. MFS7KO mice have accentuated dilative ventricular remodeling evidenced by increased left ventricular end-diastolic volume (LVEDV) and left ventricular end-systolic volume (LVESV) at the 28-day timepoint **(B-C, E-F)**. Statistical comparison (A-F) was performed using one-way ANOVA followed by Tukey's multiple comparison test. Female Smad7 fl/fl n= 19, female MFS7KO n=25; *p<0.05, **p<0.01; ^^^^p<0.0001 vs. baseline pre-infarction (pre).

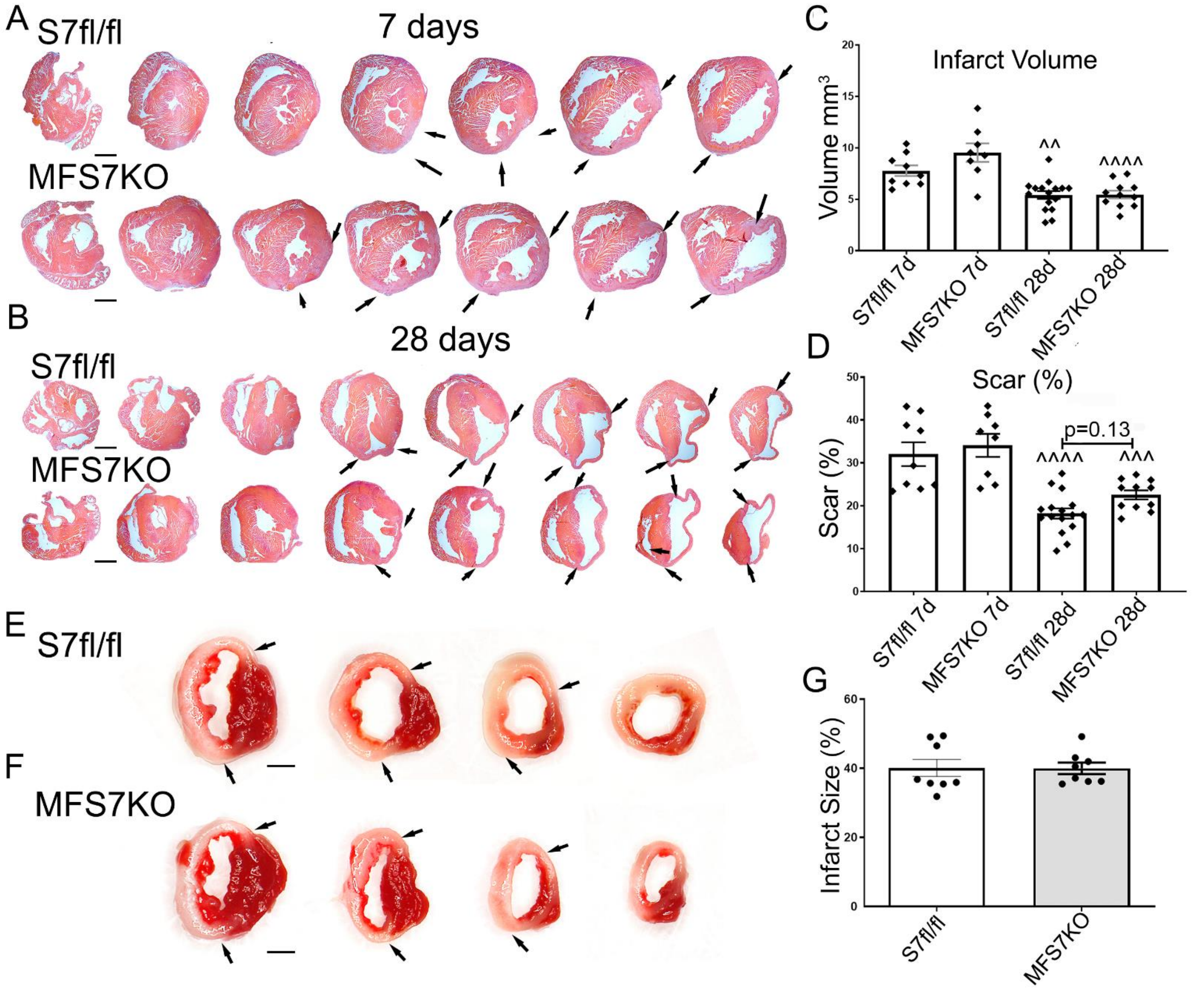

Supplemental Figure 6. Myofibroblast specific-Smad7 loss does not affect acute infarct size and scar size. Systematic morphometric histological analysis was performed by sectioning the entire infarcted heart (7 and 28-day coronary occlusion timepoints) from base to apex at 300-mm partitions and by staining the first section of each partition for hematoxylin-eosin. Representative stained sections from a Smad7 fl/fl mouse and a myofibroblast-specific Smad7 knockout (MFS7KO) animal after 7 days of permanent occlusion **(A)** and 28 days of permanent occlusion **(B)**, show the strategy (magnification 10X). Quantitative analysis shows that myofibroblast-specific Smad7 loss does not affect infarct volume **(C)** and scar size **(D)** at both timepoints. Infarct volume and percent scar size is decreased after 28 days (in comparison to the 7-day timepoint), reflecting scar contraction and progressive hypertrophic remodeling of the non-infarcted myocardium. **(E-G)** TTC staining shows that MFS7KO mice and S7 fl/fl animals have comparable acute infarct size, 48 h after coronary occlusion. For comparisons between multiple groups (C and D), one-way ANOVA was performed followed by Tukey's multiple comparison test. For comparisons between two groups (G) unpaired two-tailed Student's t test was performed. ^^p<0.01, ^^^p<0.001, ^^^^p<0.0001 vs. corresponding 7 day-timepoint (n=8-16/group). Arrows show the borders of the infarct. Scale bar = 1 mm.

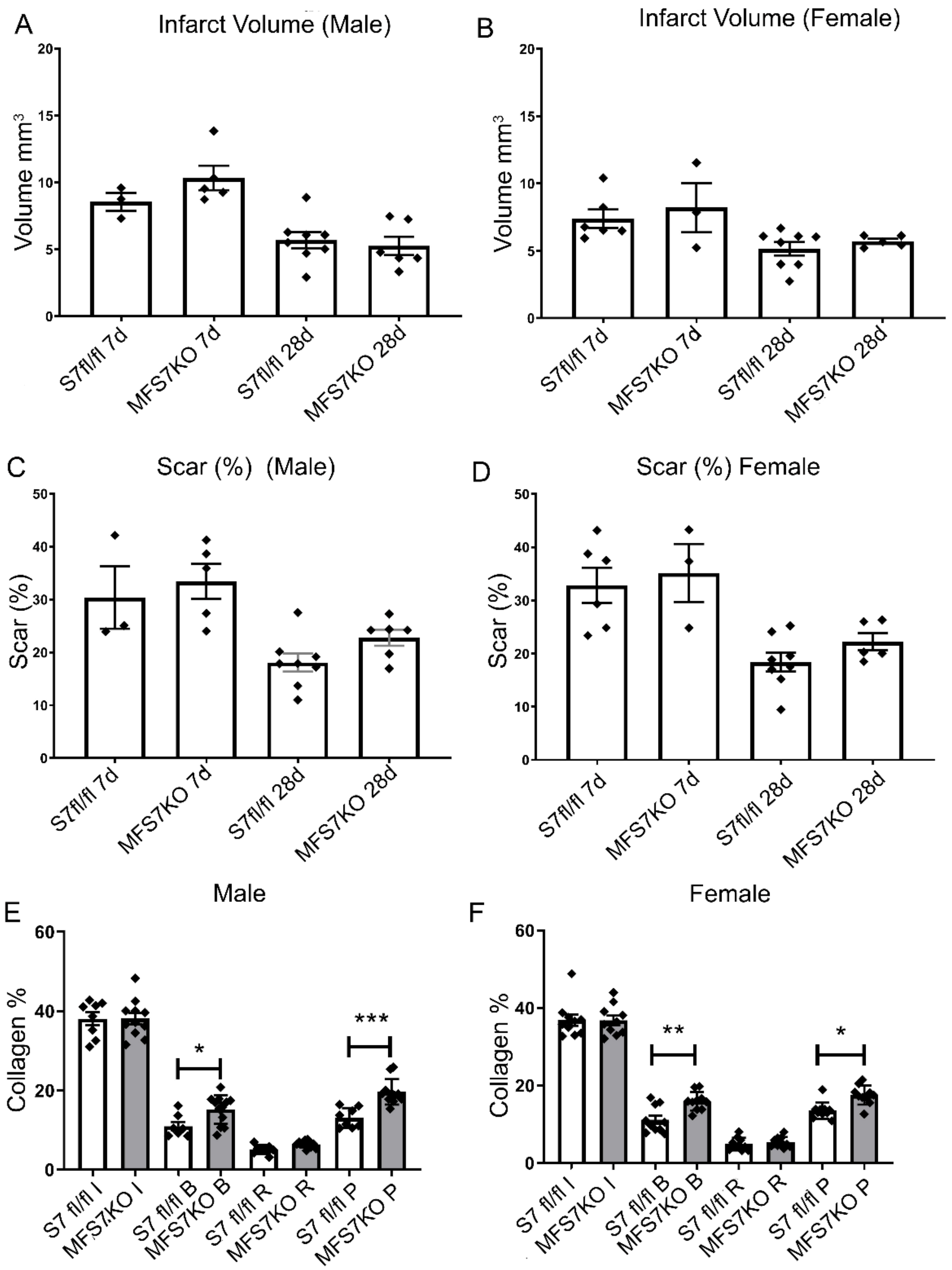

Supplemental Figure 7: The anti-fibrotic effects of myofibroblast Smad7 are noted in both male and female mice. Sex-specific analysis of scar morphometry and collagen deposition are shown. Myofibroblast-specific Smad7 loss does not affect infarct volume **(A-B)** and scar size **(C-D)**, 7 or 28 days after infarction, in both male and female mice (n=3-8/group). (**E-F**) Myofibroblast-specific Smad7 KO mice exhibit accentuated collagen deposition in the border zone (B) and in the papillary muscle (P) 28 days after infarction in both male and female groups (n= 7-12/group). Statistical comparison (A-F) was performed using one-way ANOVA followed by Tukey's multiple comparison test. *$p<0.05$, **$p<0.01$, ***$p<0.001$

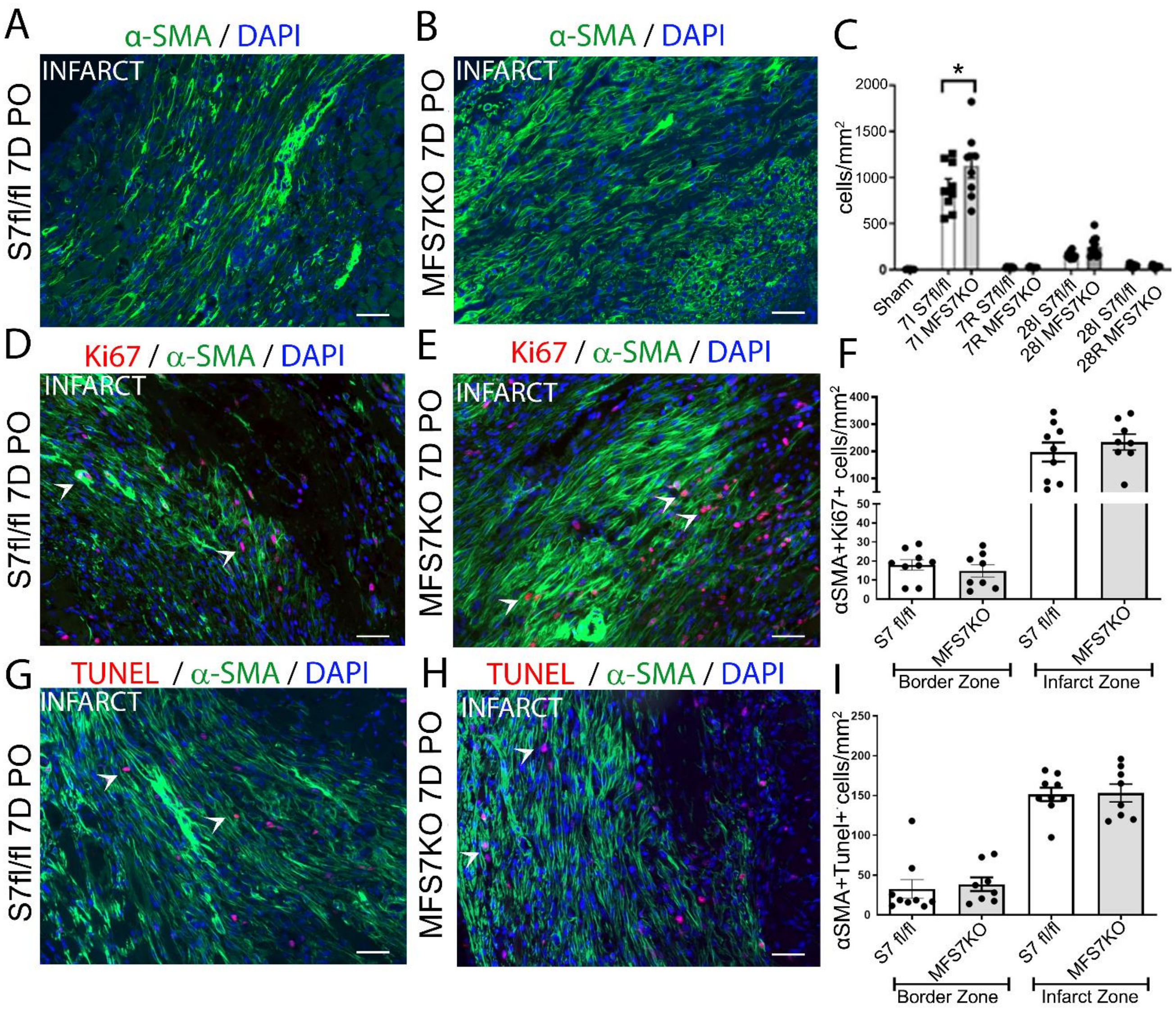

Supplementary Figure 8. Myofibroblast-specific Smad7 KO mice have increased myofibroblast infiltration 7 days after infarction, but exhibit no differences in proliferation, or apoptosis. Representative immunofluorescence α-SMA staining of Smad7 floxed (S7fl/fl) **(A)** and MFS7KO **(B)** hearts after 7 days of permanent occlusion. **(C)** Quantitative analysis of α-SMA+ non-mural cells (activated myofibroblasts) in the infarcted (I) and remote (R) areas of S7fl/fl and MFS7KO infarcted hearts at 7 and 28-days post infarction (magnification 200X), shows an increased myofibroblast density in MFS7KO infarcts, when compared to S7fl/fl infarcts**.** Representative dual immunofluorescence combining α-SMA with Ki-67 **(D-E)** and TUNEL **(G-H)** staining to assess myofibroblast proliferation and apoptosis, respectively. Quantification shows no significant differences in myofibroblast proliferation **(F)** or apoptosis **(I)** between MFS7KO and S7fl/fl mice after 7 days of infarction. Statistical comparison (C, F and I) was performed using one-way ANOVA followed by Tukey's multiple comparison test. Sham n= 3; 7 days PO S7fl/fl n=9, MFS7KO n=8; 28 days PO S7 fl/fl n=16, MFS7KO n= 14 *p<0.05. Scale bar = 50 µm.

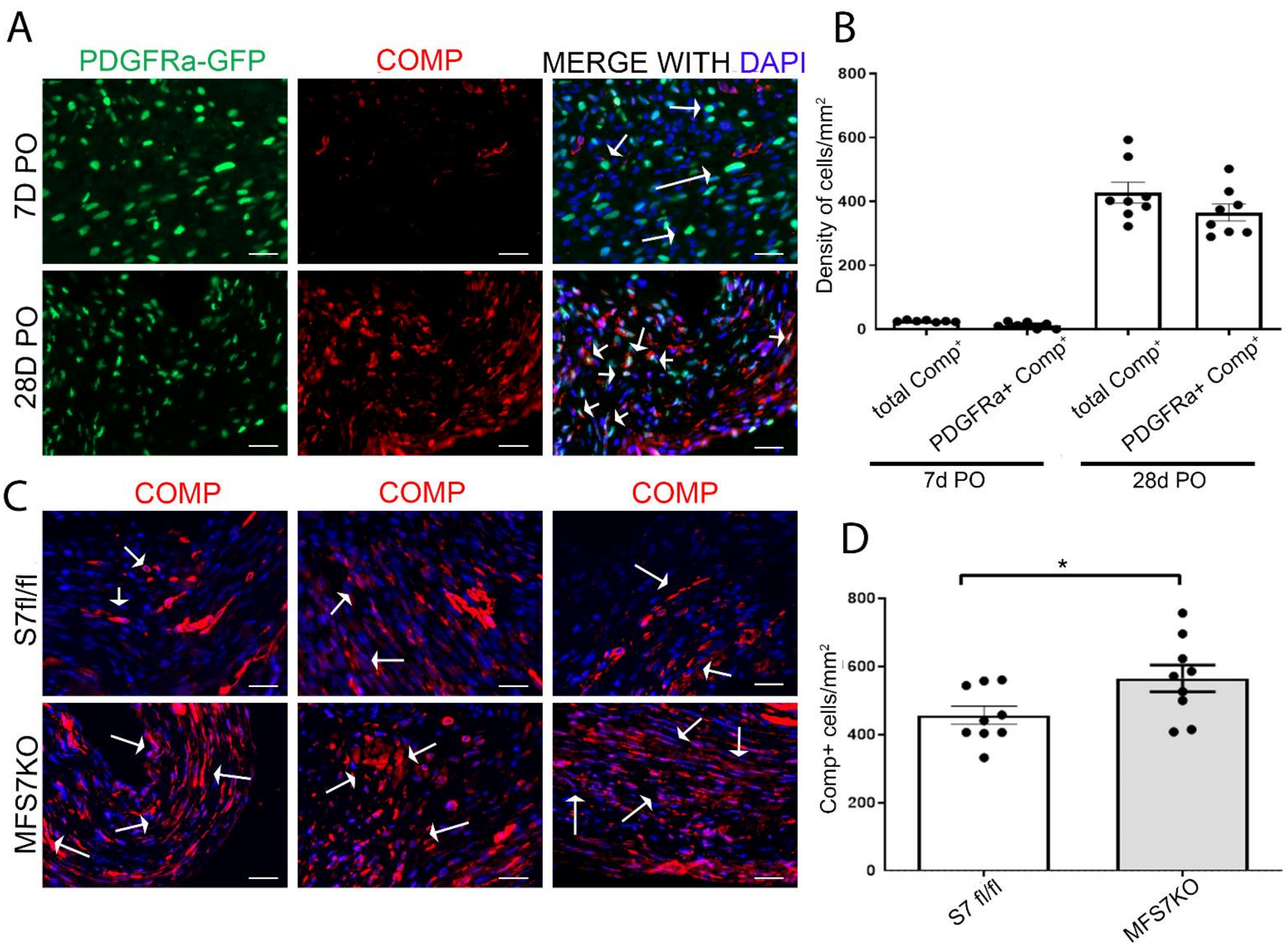

Supplemental Figure 9: Myofibroblast-specific Smad7 loss increases matrifibrocyte density in the infarcted myocardium. (A) In PDGFRα-GFP fibroblast reporter mice, dual immunofluorescence for GFP and COMP was used to identify matrifibrocytes as COMP+/PDGFRα+ cells (arrows) at 7 and 28 days after infarction. **(B)** Quantification shows negligible COMP expression at 7 days post infarction. After 28 days, abundant COMP-expressing cells are present in the mature scar, of which the vast majority are PDGFRα+ fibroblasts. **(C)** Representative images showing COMP+ matrifibrocytes (arrows) in Smad7 fl/fl and MFS7KO infarcts after 28 days of coronary occlusion. **(D)** Quantification shows that MFS7KO mice have significantly increased matrifibrocyte density in the mature scars, when compared with Smad7 fl/fl controls. For comparisons between multiple groups (B), one-way ANOVA was performed followed by Tukey's multiple comparison test. For comparisons between two groups (D) unpaired two-tailed Student's t test was performed. n=8/group; *$p<0.05$. Scale bar = 20 µm.

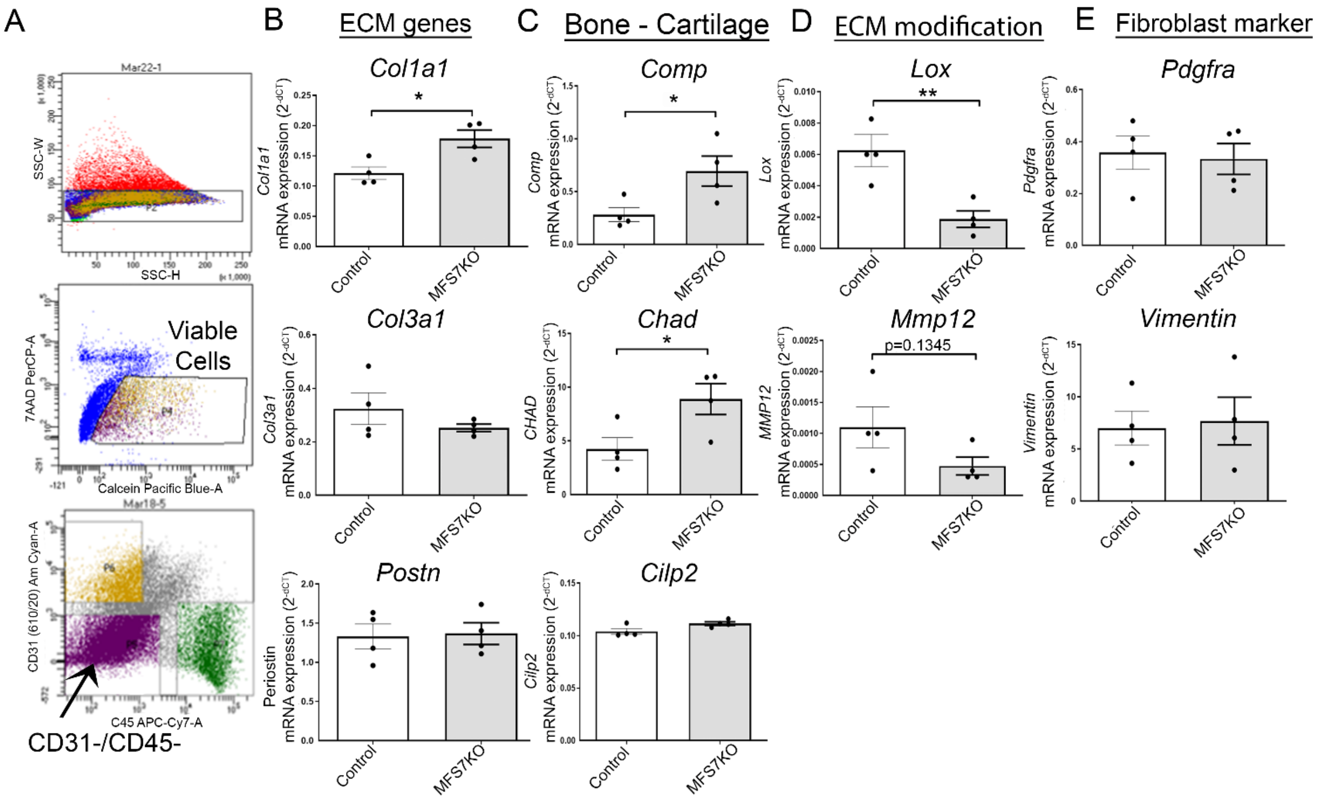

Supplemental Figure 10. Matrifibrocytes from MFS7KO infarcted hearts have increased expression of genes associated with matrix synthesis and matrifibrocytes activation. (A) Flow cytometry sorting was used to harvest viable CD31-/CD45- (interstitial cells, predominantly fibroblasts) from MFS7KO and Smad7 fl/fl infarcts (21 days after infarction). **(B-E)** qPCR comparison of genes associated with matrix modification, myofibroblast conversion and matrifibrocyte transition between CD31-/CD45- fibroblasts from S7fl/fl (control) and MFS7KO infarcts. Quantification shows that cells lacking Smad7 have increased expression of *Col1a1* and accentuated expression of the matrifibrocytes genes *Comp* and *Chad*, but comparable expression of the myofibroblast marker *Postn*. Comparisons between two groups (B-E) was performed by Mann Whitney test. n= 4/group. *p<0.05, **p<0.01.

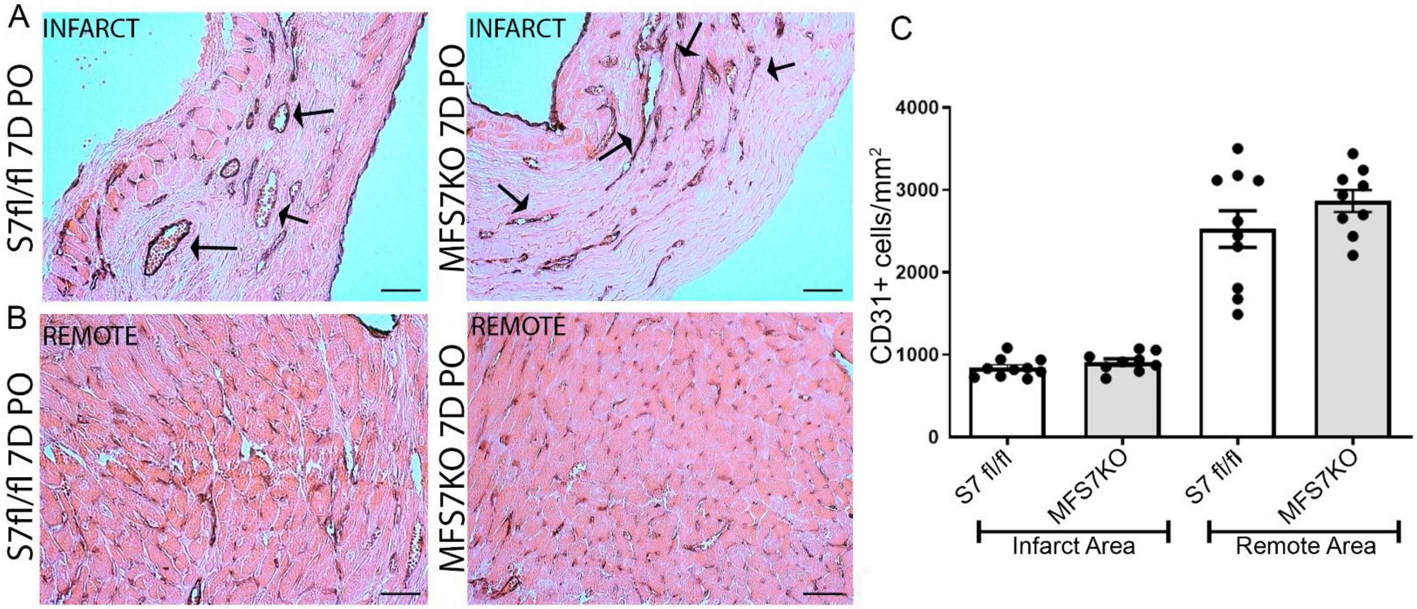

Supplemental Figure 11. Myofibroblast Smad7 does not affect infarct angiogenesis. (A-B) Representative immunohistochemical images show CD31+ microvessels (arrows) from infarcted and remote areas of Smad7 fl/fl and MFS7KO mice after 28 days of coronary occlusion. **(C)** Quantification shows that MFS7KO and Smad7 fl/fl have no significant differences in microvascular density in the infarcted and remote remodeling myocardium. Statistical comparison (C) was performed using one-way ANOVA followed by Tukey's multiple comparison test. S7 fl/fl n=10, MFS7KO n= 9. Scale bar = 50 µm.

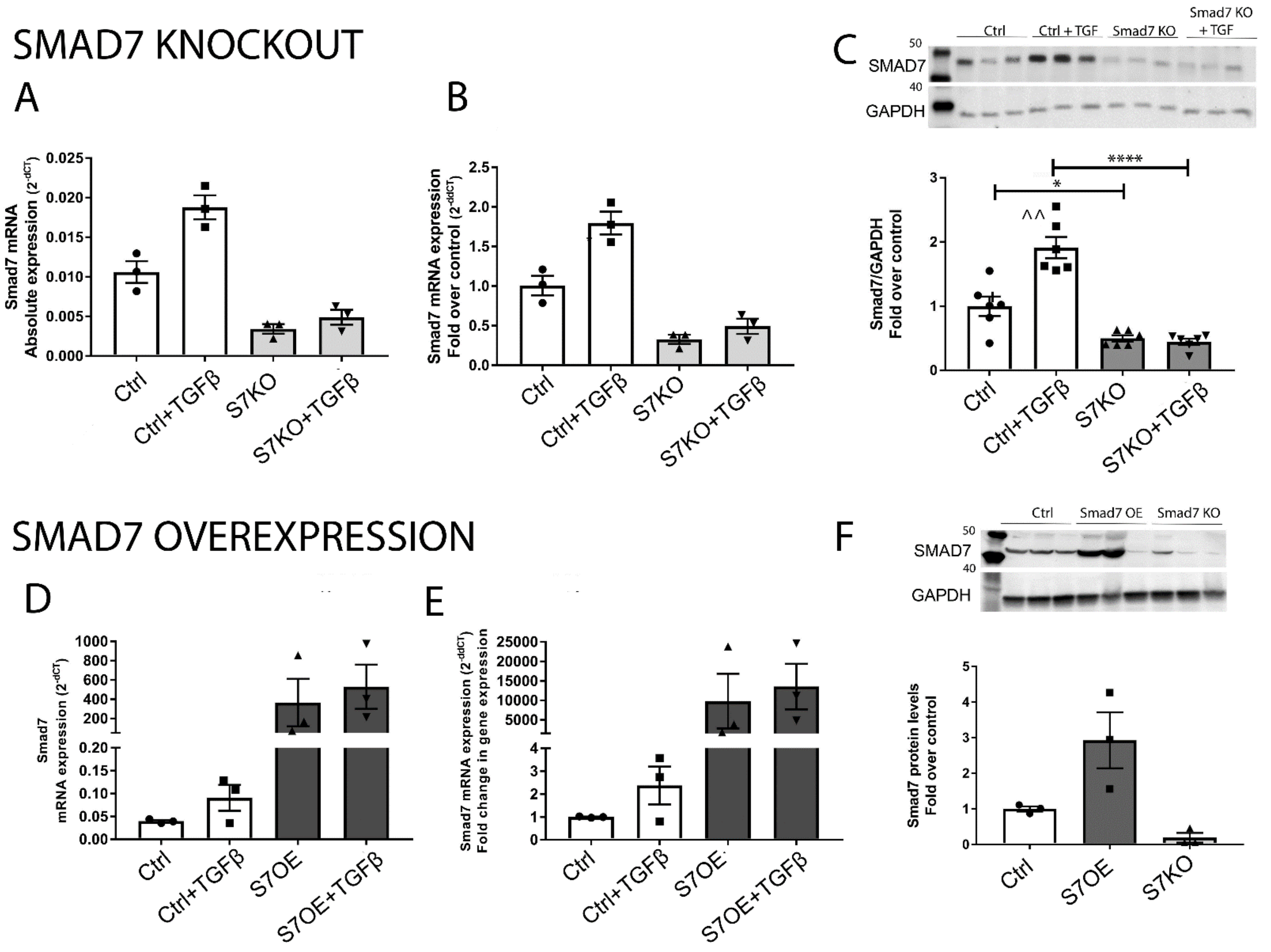

Supplemental figure 12. Documentation of Smad7 loss and overexpression in cardiac fibroblasts. Comparison of Smad7 mRNA expression **(A-B)** and protein levels **(C)** in cardiac fibroblasts harvested from Smad7 fl/fl mice and transfected with adenovirus expressing Cre-recombinase (Smad7 Knockout fibroblasts; S7KO) or transfected with Empty adenovirus (Ctrl), in the presence or absence of TGF-β1 stimulation. **(D-E)** Comparison of *Smad7* mRNA expression in cardiac fibroblasts transfected with a Smad7 cDNA plasmid (Smad 7 overexpression fibroblasts, S7OE), or with a control entry vector (Ctrl), in the presence or absence of TGF-β1 stimulation. **(F)** Smad7 protein levels of Control, Smad7 OE and Smad7 KO fibroblasts with the above-mentioned in vitro strategies. mRNA expression is shown in absolute and relative to control values (Control=1). Protein levels were normalized to GAPDH and expressed as fold over control. Statistical comparison (C) was performed using one-way ANOVA followed by Tukey's multiple comparison test. (A-B) n= 3 for each condition. (C) n= 6 for each condition; *p<0.05, ***p<0.001. (D-F) n= 3 for each condition.

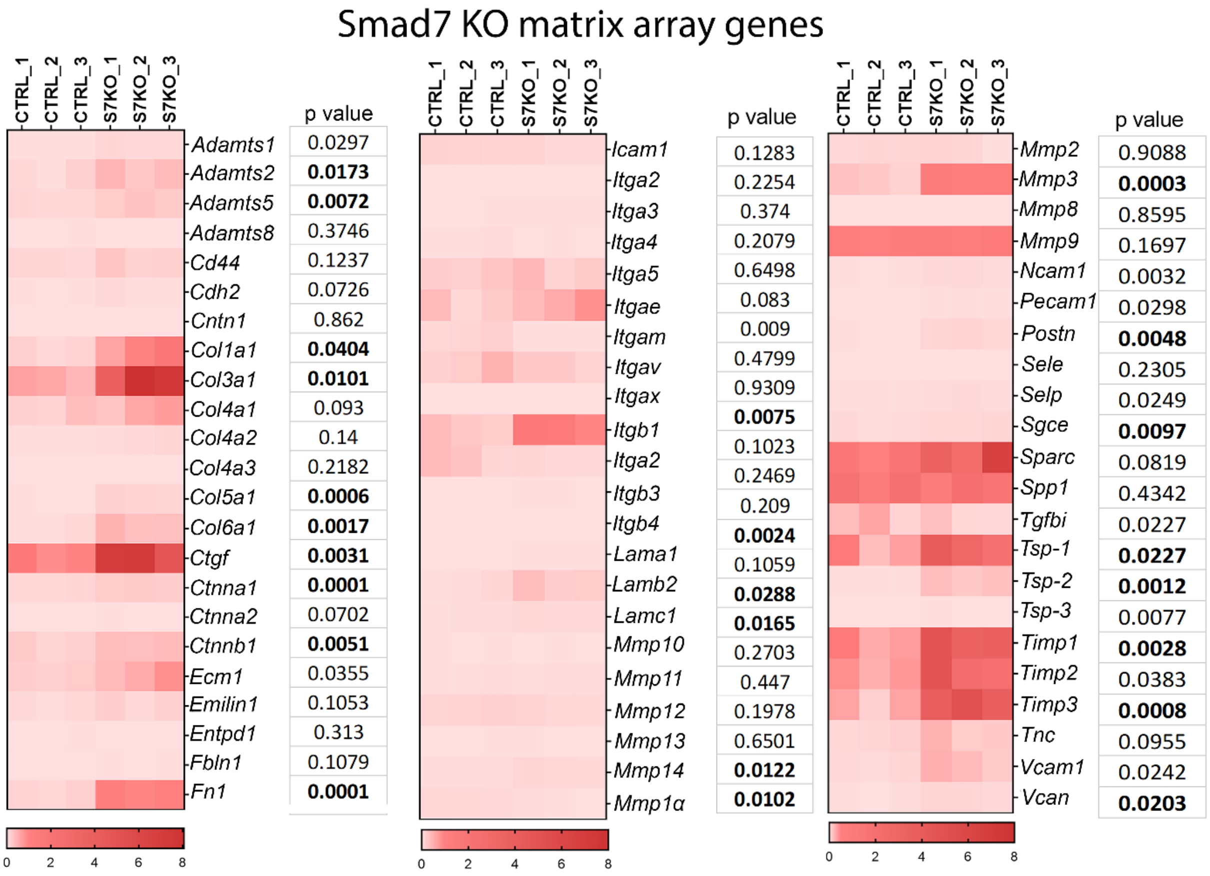

Supplemental Figure 13. Heatmap showing the effects of Smad7 loss on cardiac fibroblast expression of structural and matricellular matrix genes. Heatmap summarizing PCR array data, shows relative expression of extracellular matrix and adhesion genes of in vitro cardiac fibroblasts harvested from Smad7 fl/fl mice and transfected with adenovirus expressing Cre-recombinase (Smad7 Knockout fibroblasts; S7KO) or transfected with Empty adenovirus (CTRL). Heatmap shows that Smad7 loss accentuates expression of structural and matrix cellular genes such as Collagens and Fibronectin as well as matrix preserving TIMPs. Highlighted p-values show genes with statistically significant effects of Smad7 loss. Comparisons between two groups (CTRL and S7KO) was performed by unpaired two-tailed Student's t test. n=3/group, $p<0.05$.

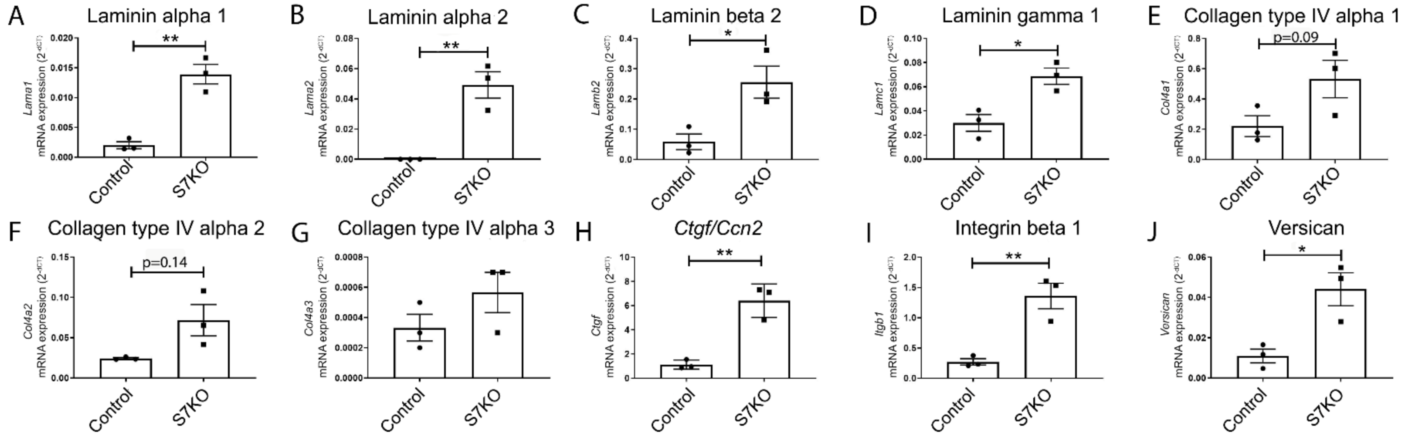

Supplemental Figure 14. S7KO cardiac fibroblasts have increased expression of TGF-β inducible matrix genes, such as Collagen IV chains, laminin chains, versican and integrin β1. Quantitative comparison of mRNA expression of matrix and cellular adhesion genes of control and S7KO cardiac fibroblasts show that Smad7 loss in cardiac fibroblasts is associated with markedly accentuated synthesis of basement membrane genes, such as Laminin chains α1, α2, β2 and γ1 **(A-D)**, Collagen IV chains alpha -1, -2, -3 **(E-G)** and TGF-β–inducible genes associated with fibroblast activation such as *Ctgf* **(H)** and Integrin beta 1 **(I)**. Comparisons between two groups (A-J) was performed by unpaired two-tailed Student's t test. n= 3 for each condition; *p<0.05, **p<0.01.

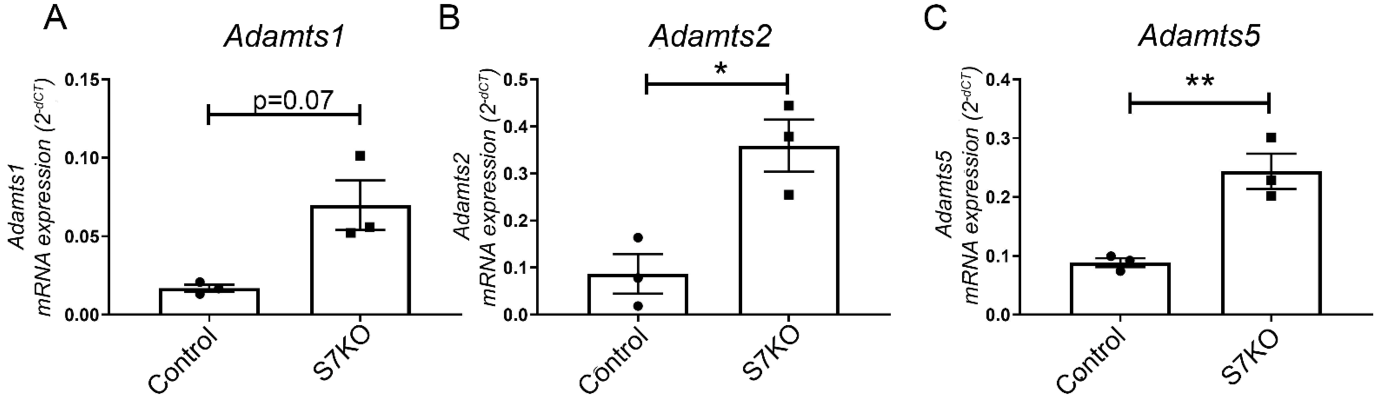

Supplemental Figure 15. Smad7 loss in cardiac fibroblasts is associated with increased expression of ADAMTS protease family members. (A-C) Quantitative comparison of mRNA expression of proteases of the family of disintegrin-like and metalloprotease with thrombospondin (ADAMTS) of control and S7KO cardiac fibroblasts, show that Smad7 loss in cardiac fibroblasts is associated with increased expression of *Adamts1*, *Adamts2* and *Adamts5*. Comparisons between two groups (A-C) was performed by unpaired two-tailed Student's t test. n= 3 for each condition. *p<0.05, **p<0.01.

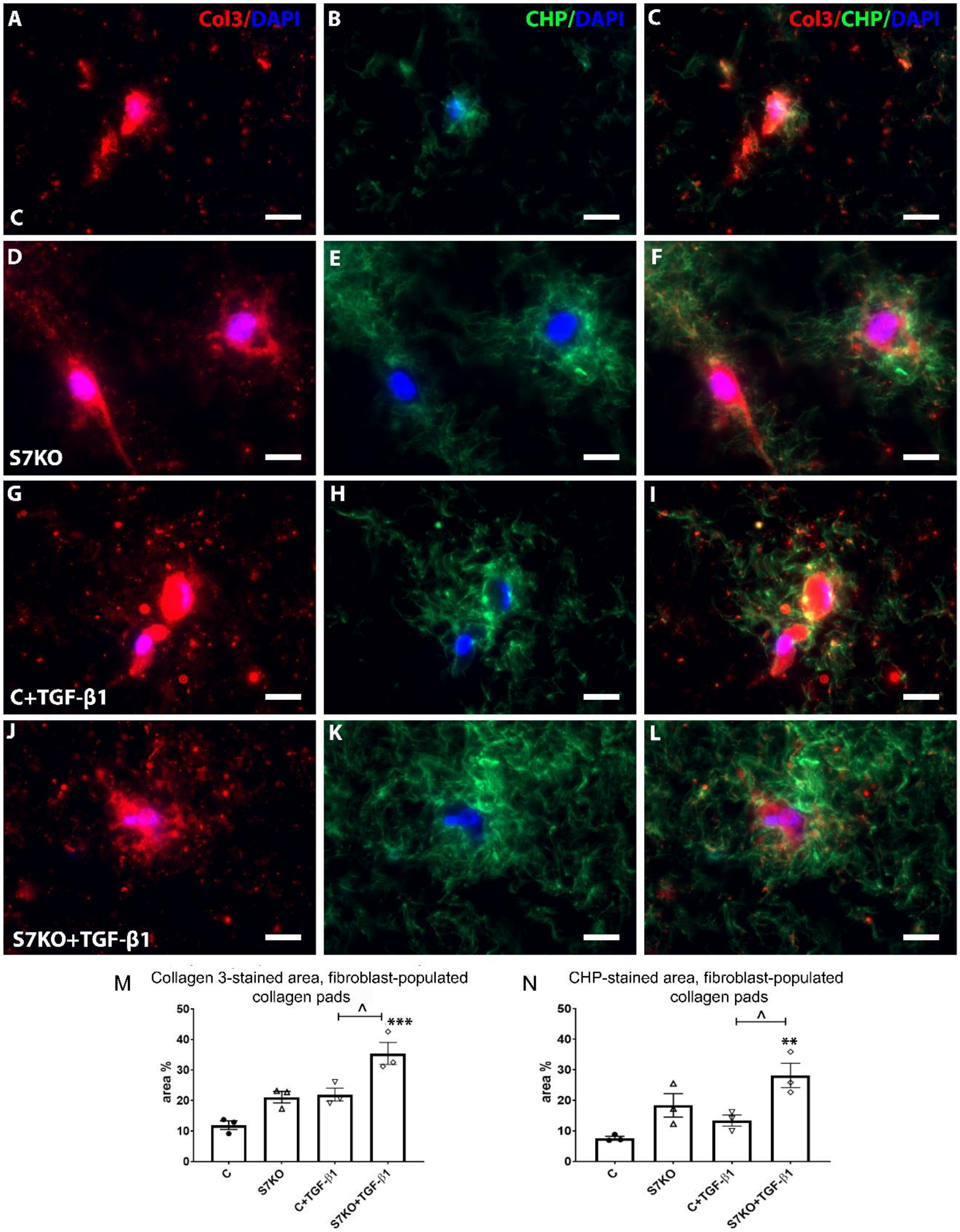

Supplemental Figure 16. Smad7 loss increases both de novo collagen synthesis and its denaturation. (A-L) Representative images of dual staining for Collagen 3 and CHP (which labels denatured collagen) staining in type I collagen pads populated with control (C) or Smad7KO fibroblasts, in the presence or absence of TGF-β1 (10 ng/mL). **(M-N)** Quantification shows that when exposed to TGF- β, S7KO fibroblasts have increased collagen III staining, reflecting increased de novo collagen synthesis and deposition, but also enhanced CHP-stained area, indicating a higher collagen denaturation than TGF-β treated control fibroblasts. Statistical comparison (M-N) was performed using one-way ANOVA followed by Tukey's multiple comparison test. n= 3/group. ^p<0.05; **p<0.01, ***p<0.001 vs. corresponding condition without TGF-β treatment. Scale bar = 20 μm.

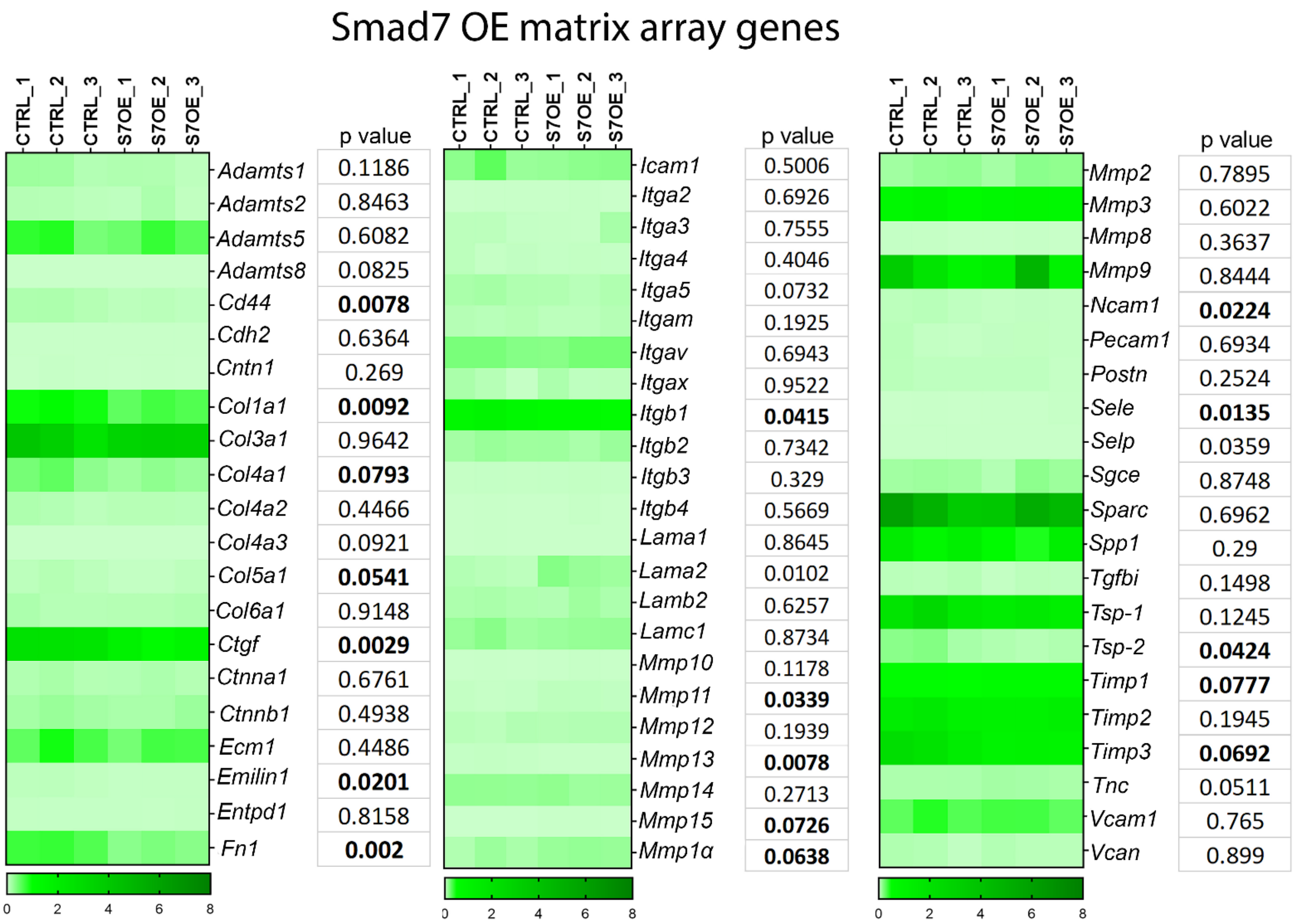

Supplemental Figure 17. Heatmap representation of the effects of Smad7 overexpression effects on cardiac fibroblast expression of structural and matricellular matrix genes. Heatmap summarizing PCR array data, shows relative expression of extracellular matrix and adhesion genes of in vitro cardiac fibroblasts transfected with a Smad7 cDNA plasmid (Smad 7 overexpression fibroblasts, S7OE) or with a control entry vector (CTRL). Heatmap shows that Smad7 overexpression decreases expression of structural and matricellular matrix genes such as Collagen, Fibronectin and reduces synthesis of *Ccn2* and integrin β1. Highlighted p-values show genes that had statistically significant differences in expression. Comparisons between two groups was performed by unpaired two-tailed Student's t test. n=3 per group, $p<0.05$.

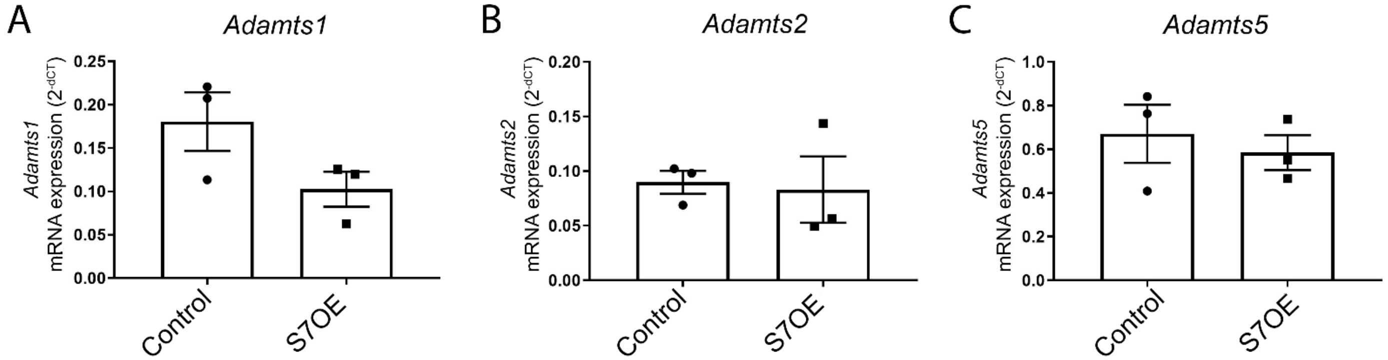

Supplemental Figure 18. Effect of Smad7 overexpression on cardiac fibroblast expression of ADAMTS protease family members. (A-C) Quantitative comparison of mRNA expression of proteases of the family of disintegrin-like and metalloprotease with thrombospondin (ADAMTS) of control and Smad7 overexpressing (S7OE) cardiac fibroblasts, show that Smad7 overexpression had no effects on *Adamts1*, *Adamts2* and *Adamts5* expression, when compared to control. Comparisons between two groups (A-C) was performed by unpaired two-tailed Student's t test. n=3 for each condition.

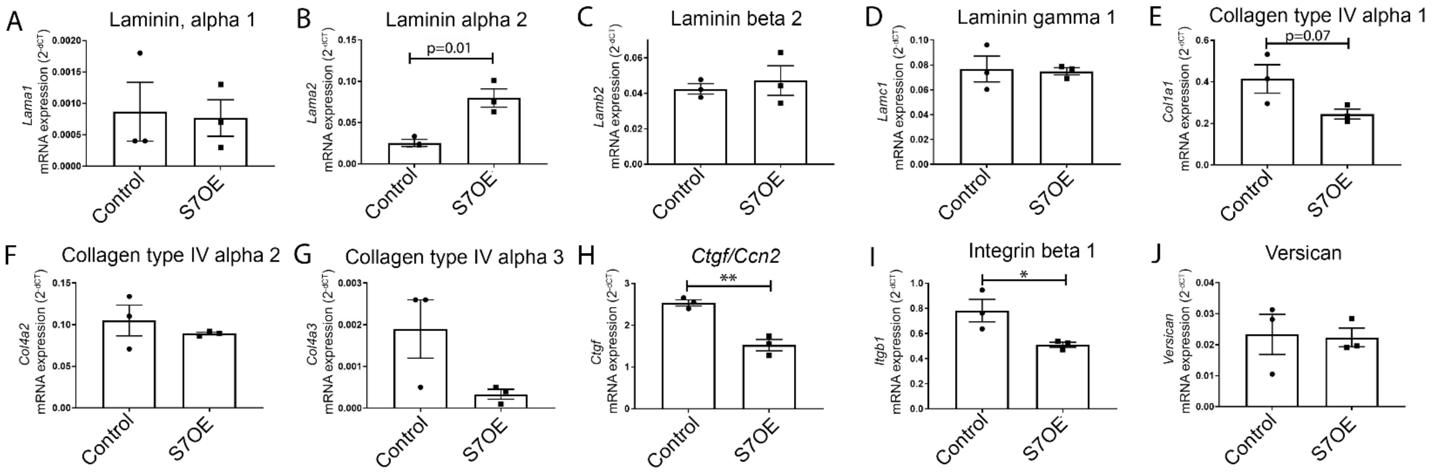

Supplemental Figure 19. Smad7 overexpression increases laminin α2 synthesis and reduces expression of CCN2 and integrin β1. Quantitative comparison of mRNA expression of matrix and cellular adhesion genes of control and S7KO cardiac fibroblasts show that Smad7 overexpression in cardiac fibroblasts had modest effects on Collagen IV and Laminin chains **(A-G)**, only increasing Laminin α2 expression **(B)**. Expression of other TGF-β inducible genes such as *Ctgf/Ccn2* **(H)** and Integrin β1 **(I)** was restrained by Smad7 overexpression. Comparisons between two groups (A-J) was performed by unpaired two-tailed Student's t test using Welch's correction for unequal variances. n= 3 for each condition; *p<0.05, **p<0.01.

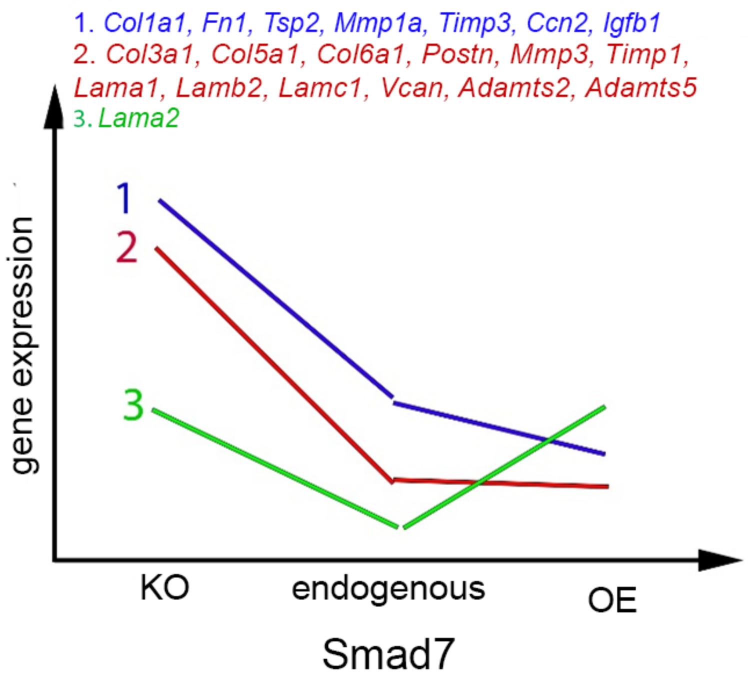

Supplemental Figure 20. Schematic illustration of the effects of endogenous Smad7 on matrix gene expression, as suggested by the loss and gain-of-function experiments Schematic depicting the different effects of in vitro Smad7 modulation on cardiac fibroblasts structural and matricellular gene expression. Genes restrained by Smad7 are grouped into three categories based on their expression pattern in relation to Smad7 levels. Pattern 2 (red) shows genes in which endogenous expression of Smad7 in fibroblasts is sufficient to maximally restrain gene expression. Pattern 1 (blue) indicates genes in which forced overexpression of Smad7 further reduces gene expression Only one gene (*Lama2*, pattern 3, green) showed a pattern consistent with inhibitory effects of endogenous Smad7, accompanied by induction upon forced overexpression.

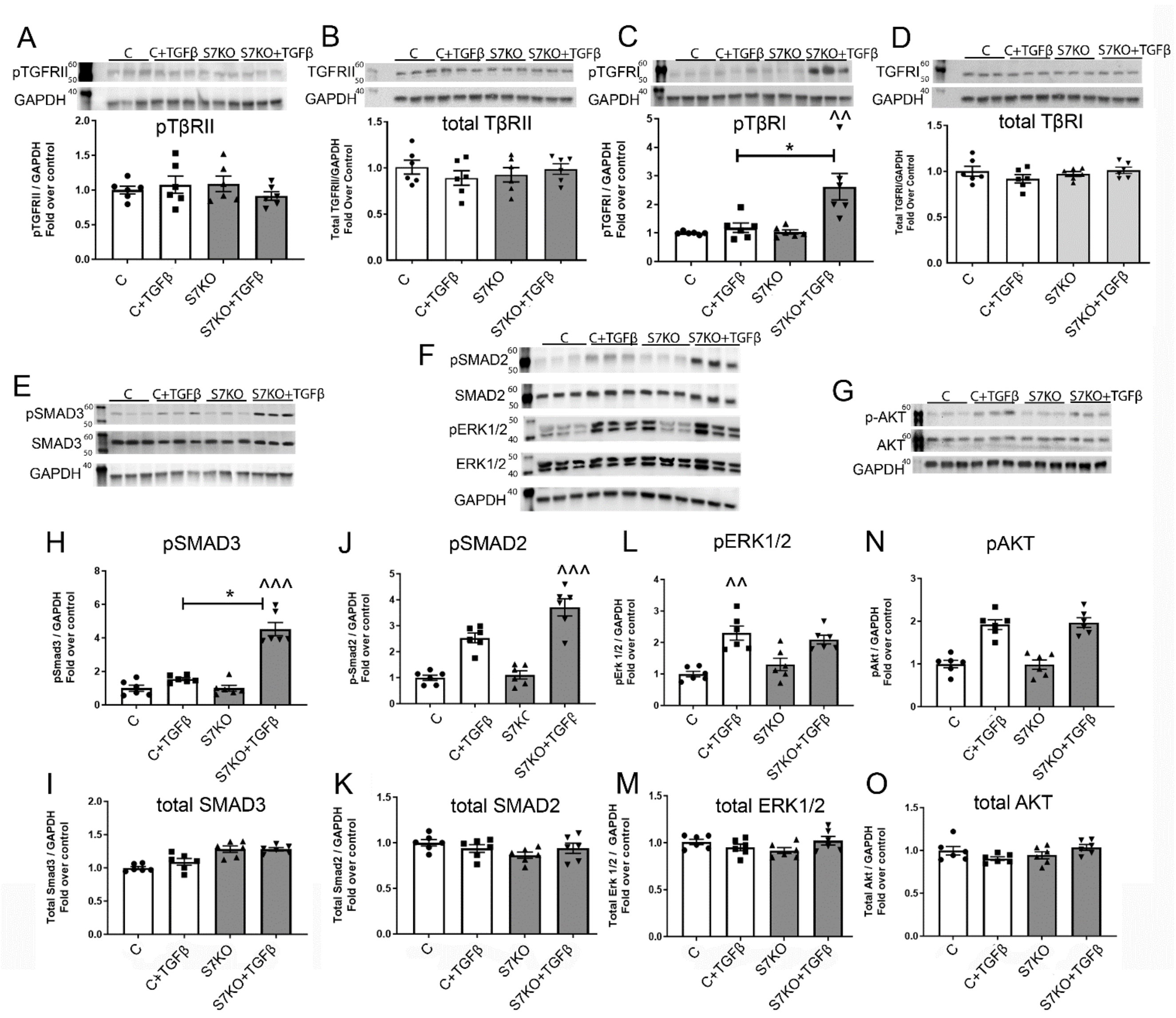

Supplemental Figure 21. Smad7 restrains the long-term effects of TGF-β (24 hours stimulation) on Smad2, Smad3 and TGF-β1 receptor activation, without affecting non-canonical ERK and AKT activity. (A) Smad7 loss does not affect constitutive TβRII activity, or total TβRII levels **(B)**; however, TβRI activation is accentuated in S7KO fibroblasts after 24 h stimulation with TGF-β **(C)** without affecting total TβRI levels **(D)**. In order to examine the effects of Smad7 loss on Smad-dependent and non-Smad pathways, we performed western blotting for p-Smad3, Smad3, p-Smad2, Smad2, p-ERK1/2, ERK1/2, pAKT and AKT **(E-G)**. pSmad3 **(H)** and pSmad2 **(J)** phosphorylation induced by 24 hours treatment of TGF-β are accentuated in S7KO fibroblasts, without affecting total Smad3 **(I)** or Smad2 levels **(K)**. Smad-independent ERK1/2 **(L)** and AKT **(N)** activation induced by TGF-β, as well as their total protein levels **(M, O)**; are not affected by Smad7 loss. Statistical comparison (A-D, H-O) was performed using one-way ANOVA, followed by Tukey's multiple comparison test. n=6 per group, *p<0.05, ***p<0.001; ^^p<0.01, ^^^p<0.001 vs. unstimulated condition.

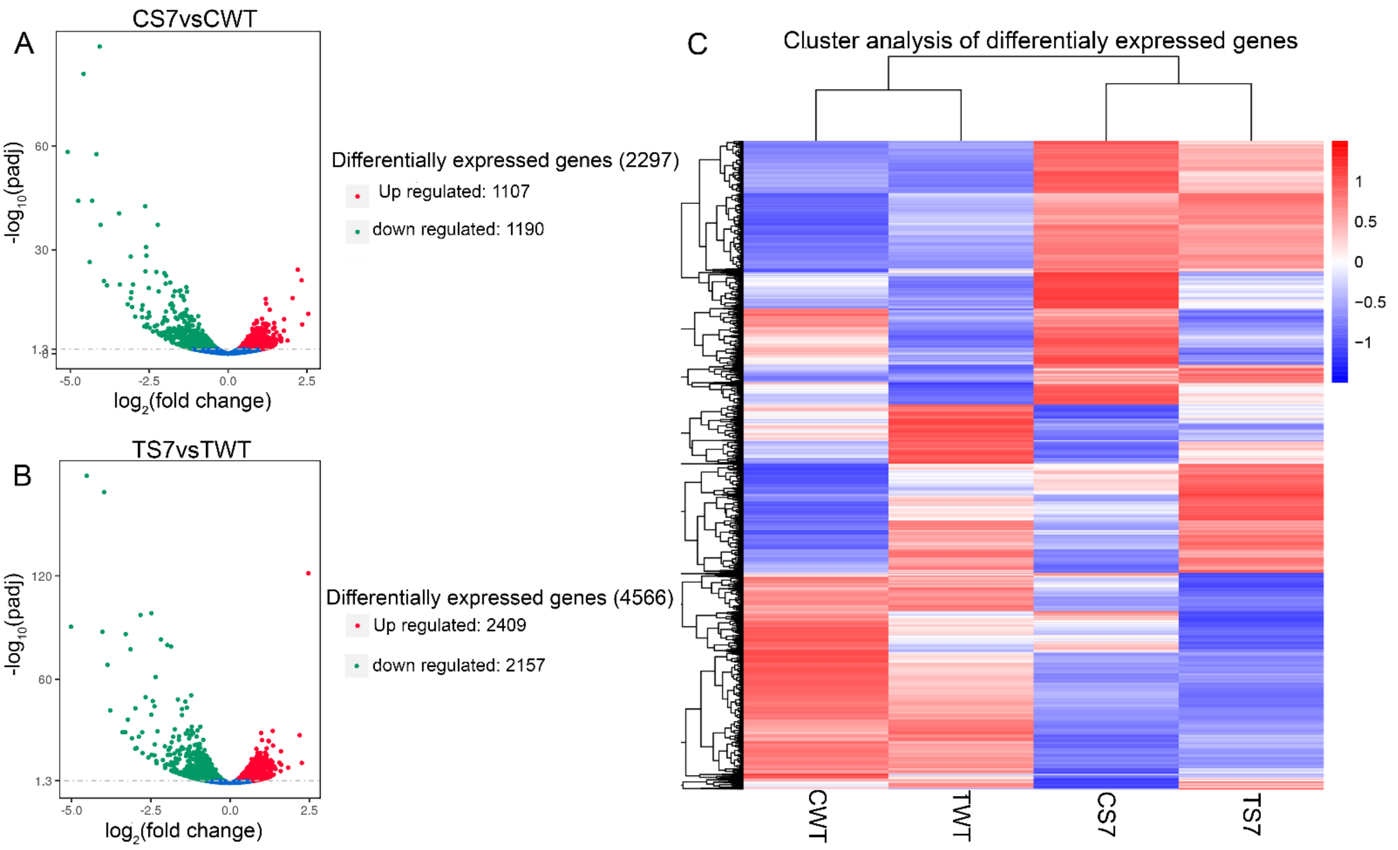

Supplemental Figure 22. Effects of Smad7 loss on the transcriptome of cardiac fibroblasts. (A-B) Volcano plot summarizing the differentially up and downregulated gene expression in the RNA-seq transcriptomic comparisons between nontreated Smad7 knockout cardiac fibroblast (CS7), TGF-β treated Smad7 knockout fibroblasts (TS7); and their corresponding controls wild type nontreated fibroblasts (CWT) and wild type TGF-β treated fibroblasts (TWT). **(C)** Heatmap representation showing color-coded expression of differentially expressed genes between all conditions. Genes were clustered using Deseq2 R software package and ranked by differential gene expression as log2 (fold change) between each comparison group. Genes with an adjusted P-value ≤ 0.05 were considered to be differentially expressed. n=3 for each condition.

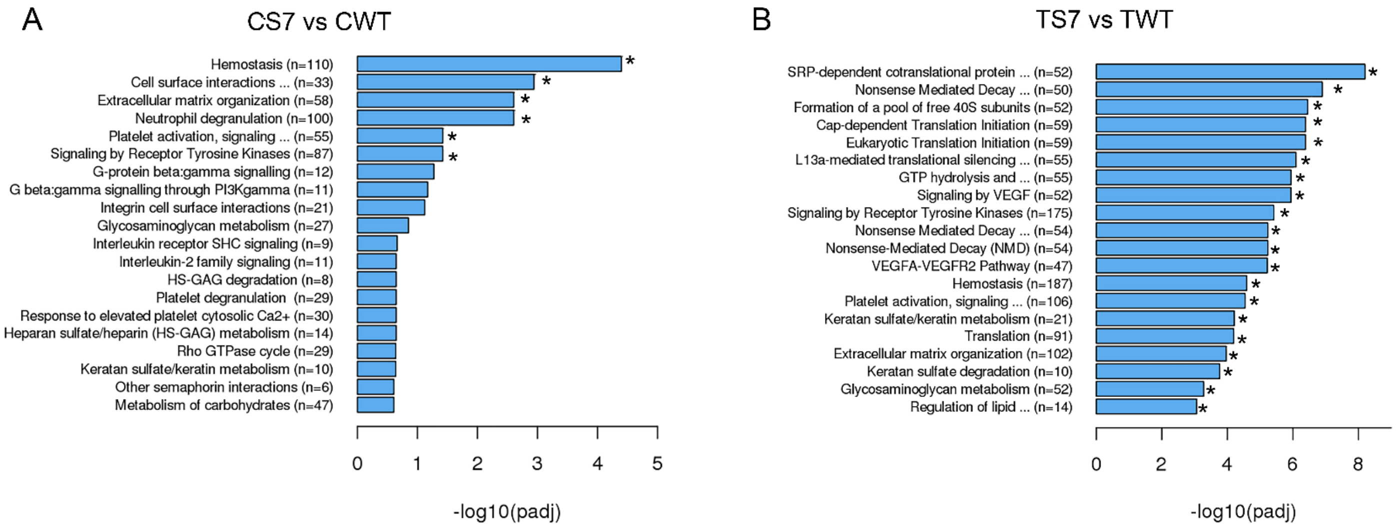

Supplemental Figure 23. Differentially expressed categories of genes regulated by Smad7 in cardiac fibroblasts transcriptome (A) Bioinformatic analysis comparison of the transcriptome of unstimulated wild type (CWT) vs Smad7 KO fibroblasts (CS7) using the Reactome Pathway Database identified 6 differentially expressed categories. Of these, only “Signaling by receptor tyrosine kinases (RTK)”, was related to intracellular signaling pathways (padj=0.037). **(B)** Comparison of the transcriptome of TGF-β-stimulated wild type (TWT) vs TGF-β treated Smad7 KO fibroblasts (TS7) using Reactome identified 53 categories. Of these, 8 categories were related to intracellular signaling cascades and were represented by >25 differentially regulated genes (Table 1). Differentially regulated categories are marked with * in the graphs, padj<0.05; n indicates the number of genes grouped in each category.

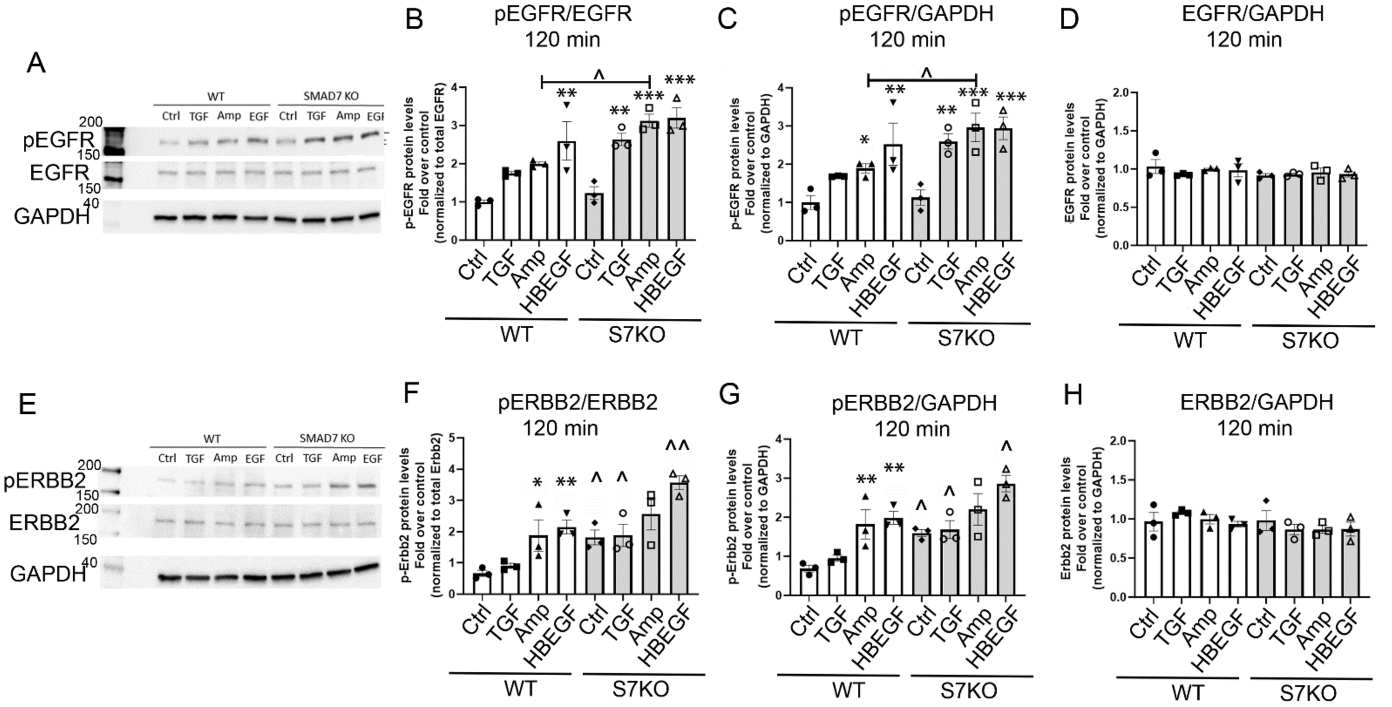

Supplemental Figure 24. Smad7 restrains Erbb2 activation in a ligand-independent manner and limits prolonged amphiregulin-mediated EGFR activation. Representative blots and quantitative analysis of the effects of Smad7 on EGFR/Erbb1 and Erbb2 activation in the presence or absence of TGF-β1 and the Erbb activators amphiregulin and HB-EGF (120 min stimulation); on fibroblasts harvested from Smad7 fl/fl mice and transfected with adenovirus expressing Cre-recombinase (Smad7 Knockout fibroblasts; S7KO) or transfected with Empty adenovirus (WT). **(A-D)** Smad7 loss did not affect EGFR activity at baseline or after stimulation with TGF-β1, or HB-EGF. However, Smad7 loss accentuated amphiregulin-mediated EGFR activation. **(E-H)** Markedly increased ERBB2 activation was observed in S7KO fibroblasts both at baseline and upon stimulation with TGF-β1 and HB-EGF. Total ERBB1 or ERBB2 levels were not affected. Statistical comparison (B-D, F-H) was performed using one-way ANOVA, followed by Tukey’s multiple comparison test. n=3, * p<0.05, **p<0.01, ***p<0.001 versus corresponding nontreated condition; ^p<0.05; ^^p<0.01; versus corresponding WT condition.

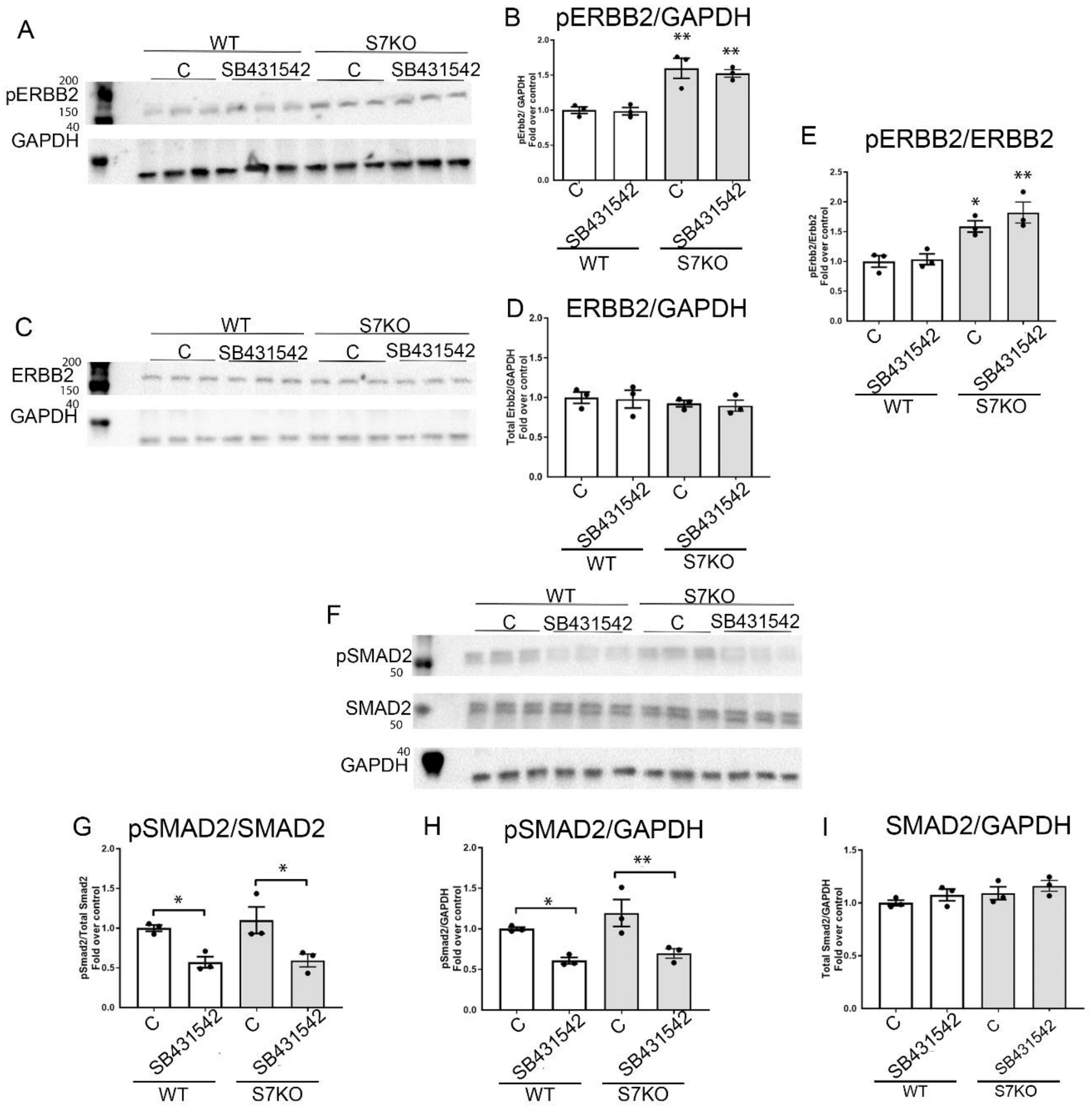

Supplemental Figure 25. Smad7 effects on Erbb2 activity are independent of TGF-β. (A-E) Representative blots and quantitative analysis of the effects of Smad7 on ERBB2 activation in Smad7 KO and WT cells in the presence or absence of the TGF-β receptor inhibitor SB431542, show increased ERBB2 phosphorylation in S7KO cells is unaffected by TGF-β receptor inhibition, suggesting TGF-β independent effects of Smad7 on ERBB2 activation. **(F-I)** Representative blots and quantitative analysis of Smad2 activation in WT and S7KO fibroblasts, in the presence or absence of TGF-β receptor inhibitor, shows decreased pSmad2 levels when TGF-β signaling is disrupted with SB431542 in both WT and S7KO fibroblasts. Statistical comparison (B-E, G-I) was performed using one-way ANOVA, followed by Tukey's multiple comparison test. n=3, * $p<0.05$, ** $p<0.01$, vs. WT.

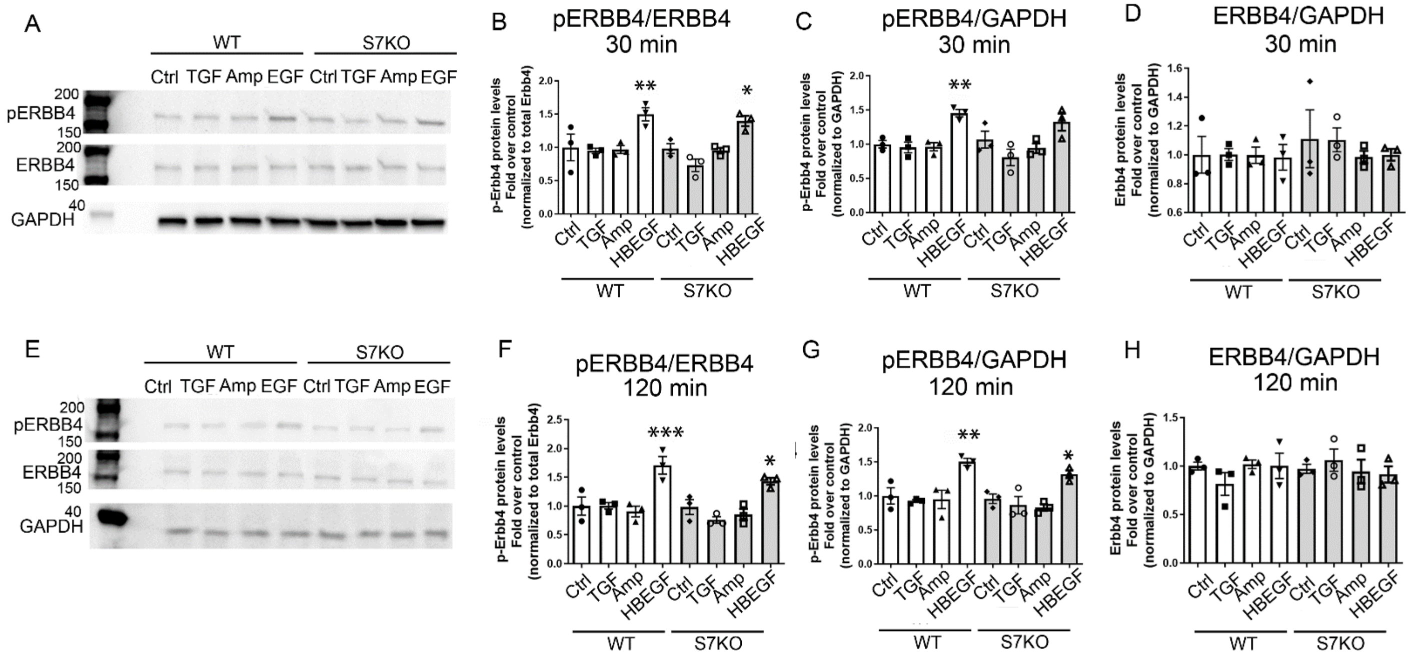

Supplemental Figure 26. Smad7 does not affect Erbb4 activation in the presence or absence of TGF-β1, amphiregulin, or HB-EGF stimulation. (A-H) Representative blots and quantitative analysis of the effects of Smad7 on ERBB4 activation in the presence or absence of TGF-β1 and the Erbb activators amphiregulin and HB-EGF 30 **(A-D)** and 120 **(E-H)** min stimulation; on fibroblasts harvested from Smad7 fl/fl mice and transfected with adenovirus expressing Cre-recombinase (Smad7 Knockout fibroblasts; S7KO) or transfected with Empty adenovirus (WT). ERBB4 activity was increased by after 30- and 120-min stimulation with HB-EGF, but not with TGF-β1 or Amphiregulin. Smad7 loss had no effect on either baseline or any of the stimuli conditions. ERBB4 total levels were not affected by Smad7 loss. Statistical comparison (B-D, F-H) was performed using one-way ANOVA, followed by Tukey's multiple comparison test. n=3, * $p<0.05$, ** $p<0.01$, *** $p<0.001$ versus corresponding nontreated condition).

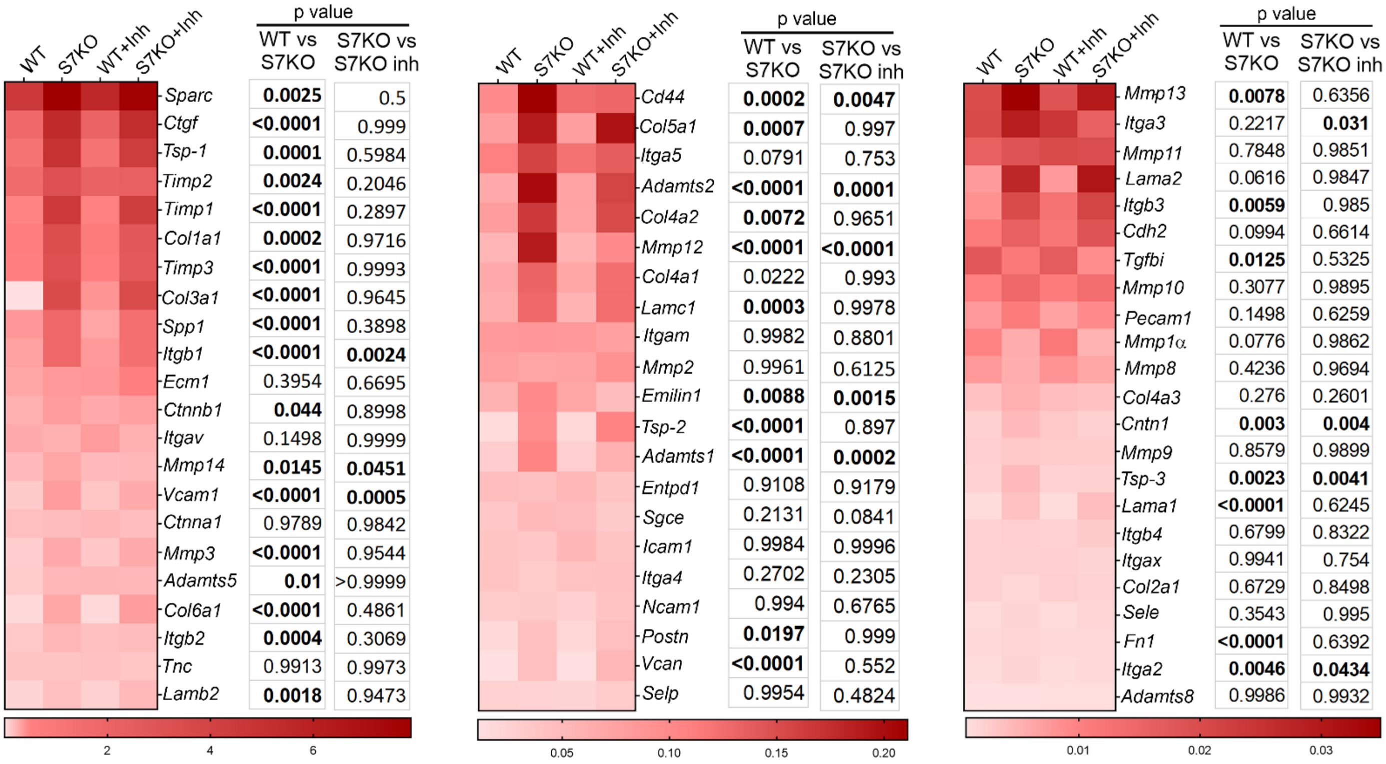

Supplemental Figure 27. Heatmap representation of the effects of Erbb1/2 inhibition on Smad7-mediated modulation of cardiac fibroblast fibrosis-associated gene expression. Heatmap summarizing PCR array data, shows relative expression of extracellular matrix and adhesion genes of in vitro amphiregulin-treated Smad7 knockout cardiac fibroblasts (S7KO) compared to control fibroblasts (WT) in presence or absence of Erbb1/2 dual inhibitor (Inh), Lapatinib. Heatmap shows that Erbb1/2 inhibition in cardiac fibroblasts attenuated the effects of Smad7 loss on expression of several profibrotic genes such as ADAMTS proteases and Collagens. Highlighted p-values show genes that had statistically significant differences between WT vs S7KO or S7KO vs S7KO treated with dual Erbb1/2 inhibitor conditions. For clarification purposes, genes were sorted according to their relative gene expression levels. Value shown in each condition corresponds to the average of n=3 per group. Statistical comparison was performed using one-way ANOVA, followed by Tukey’s multiple comparison test, $p<0.05$.

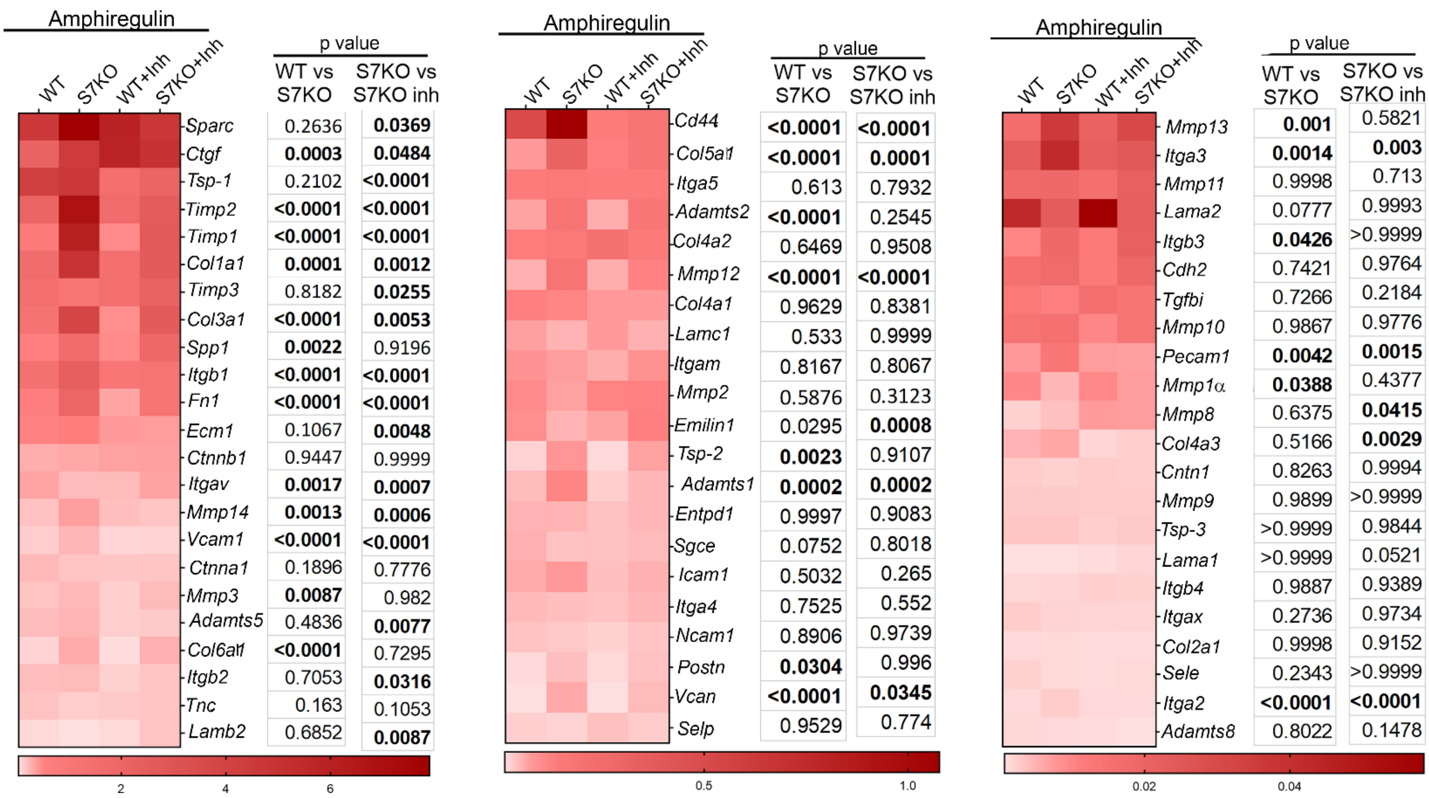

Supplemental Figure 28. Heatmap representation of the effects of Erbb1/2 inhibition on Smad7-mediated modulation of Amphiregulin-treated cardiac fibroblast fibrosis-associated gene expression. Heatmap summarizing PCR array data, shows relative expression of extracellular matrix and adhesion genes of in vitro Amphiregulin treated-Smad7 knockout cardiac fibroblasts (S7KO) compared to Amphiregulin treated control fibroblasts (WT) in presence or absence of Erbb1/2 dual inhibitor (Inh), Lapatinib. Highlighted p-values show genes that had statistically significant differences between Amphiregulin-treated: WT vs S7KO or S7KO vs S7KO treated with dual Erbb1/2 inhibitor. For clarification purposes, genes were sorted according to their relative gene expression levels. Value shown in each condition corresponds to the average of n=3 per group. Statistical comparison was performed using one-way ANOVA, followed by Tukey's multiple comparison test. $p<0.05$.

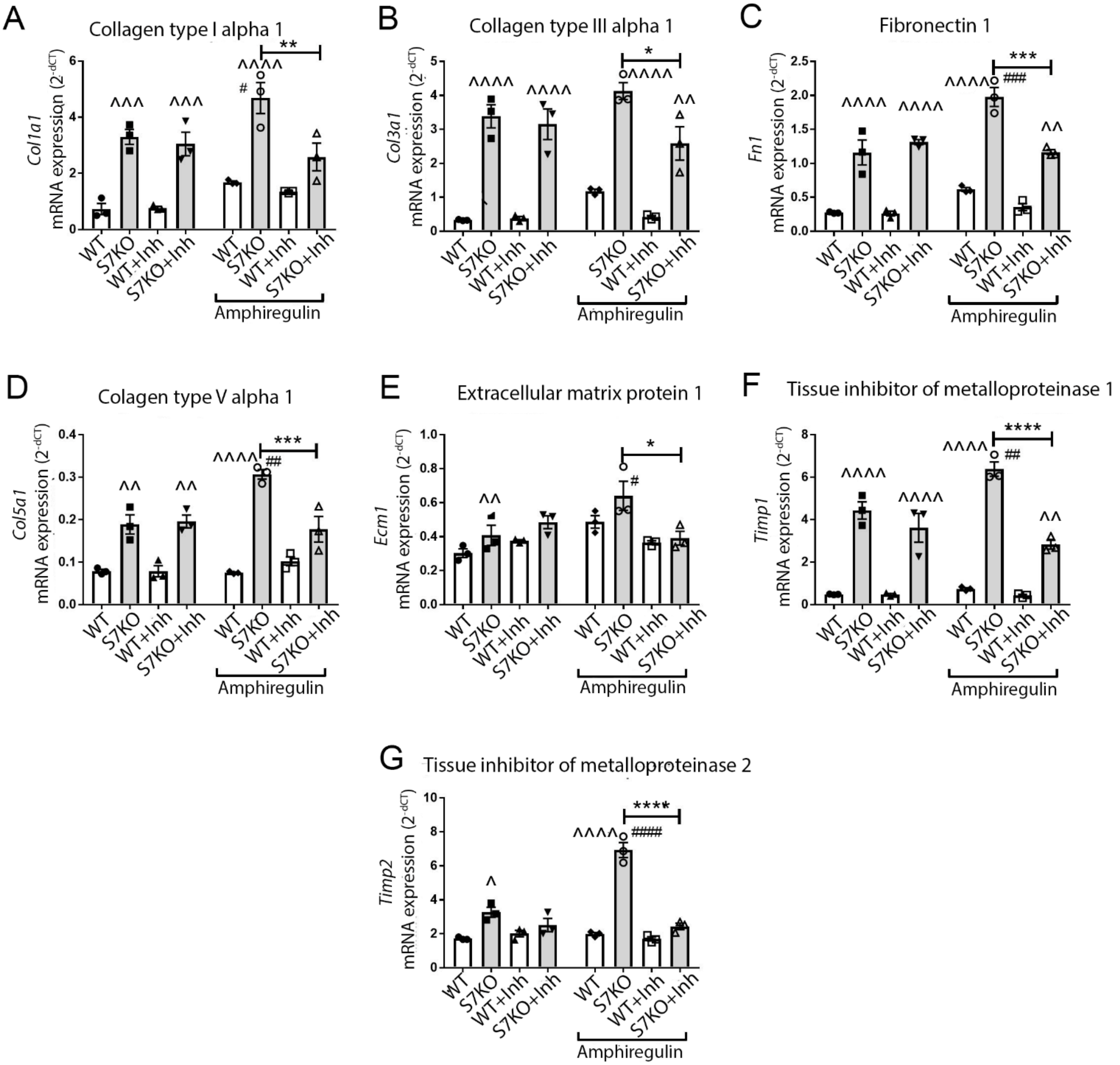

Supplemental Figure 29. Inhibition of Erbb1/2 attenuates the increased Collagen I/III/V, Fibronectin, Extracellular matrix protein 1 (*Ecm1*) and *Timp1/2* expression observed in Smad7 KO fibroblasts, only in the presence of amphiregulin stimulation. Comparison of the effects of the Erbb1/2 inhibitor lapatinib on extracellular matrix genes of Smad7 Knockout fibroblasts (S7KO) and control fibroblasts (WT), in the presence or absence of amphiregulin. **(A-C)** Increased expression of key structural extracellular matrix genes, (*Col1a1*, *Col3a1* and Fibronectin), **(D-E)** non-structural matrix genes (*Ecm1* and *Col5*) and **(F-G)** matrix-preserving anti-proteases *Timp1* and *Timp2* are not affected by Erbb1/2 inhibition in unstimulated conditions. However, the increase in gene expression observed in Smad7KO fibroblasts when Erbb receptor is activated with its ligand, amphiregulin; is attenuated after EGFR/Erbb2 inhibition. Statistical comparison (A-G) was performed using one-way ANOVA, followed by Tukey's multiple comparison test n=3; *p<0.05, **p<0.01, ***p<0.001, ****p<0.0001; ^^p<0.01, ^^^p<0.001, ^^^^p<0.0001 vs. corresponding WT; #p<0.05, ##p<0.01, ###p<0.001; ####p<0.0001 vs. same condition in absence of Amphiregulin stimulation.

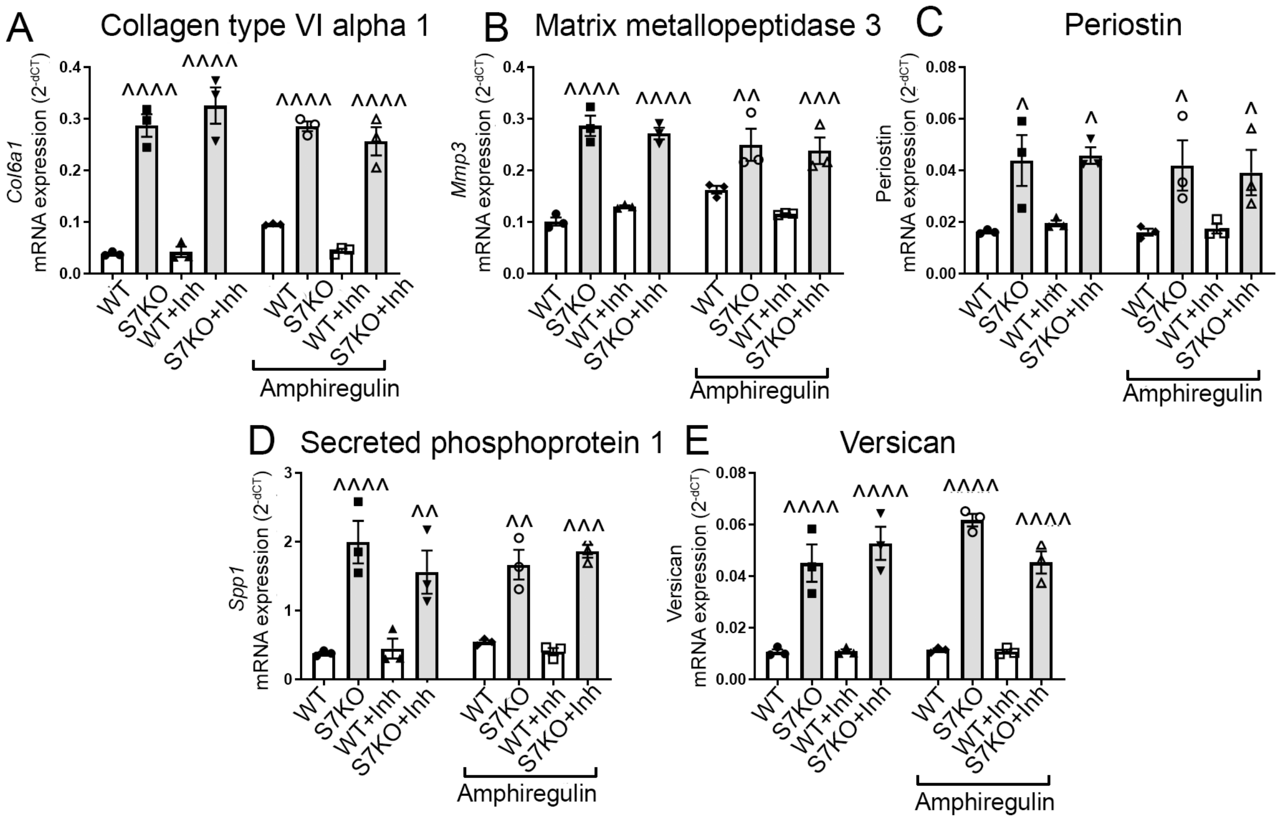

Supplemental Figure 30. Smad7-mediated inhibition of *Col6a1*, *Mmp3*, Periostin, *Spp1*/Osteopontin and Versican is not affected by Erbb1/Erbb2 inhibition, in the presence or absence or absence or amphiregulin. Comparison of the effects of the Erbb1/2 inhibitor (lapatinib) on extracellular matrix genes of Smad7 Knockout fibroblasts (S7KO) and control fibroblasts (WT) in presence or absence of amphiregulin. **(A-E)** Increased expression of *Col6a1*, *Mmp3*, Periostin, Osteopontin/Secreted phosphoprotein 1 and Versican observed in Smad7 knockout fibroblasts is not attenuated by Erbb1/2 inhibition, regardless of Amphiregulin stimulation. Statistical comparison (A-E) was performed using one-way ANOVA, followed by Tukey's multiple comparison test. n=3; ^p<0.05, ^^p<0.01, ^^^p<0.001, ^^^^p<0.0001 vs. WT.

REFERENCES:

1. Kleiter I, Song J, Lukas D, Hasan M, Neumann B, Croxford AL, Pedre X, Hovelmeyer N, Yogev N, Mildner A, et al. Smad7 in T cells drives T helper 1 responses in multiple sclerosis and experimental autoimmune encephalomyelitis. *Brain.* 2010;133(Pt 4):1067-81.
2. Lindsley A, Snider P, Zhou H, Rogers R, Wang J, Olaopa M, Kruzynska-Frejtag A, Koushik SV, Lilly B, Burch JB, et al. Identification and characterization of a novel Schwann and outflow tract endocardial cushion lineage-restricted periostin enhancer. *Dev Biol.* 2007;307(2):340-55.
3. Takeda N, Manabe I, Uchino Y, Eguchi K, Matsumoto S, Nishimura S, Shindo T, Sano M, Otsu K, Snider P, et al. Cardiac fibroblasts are essential for the adaptive response of the murine heart to pressure overload. *J Clin Invest.* 2010;120(1):254-65.
4. Conway SJ, and Molkentin JD. Periostin as a heterofunctional regulator of cardiac development and disease. *Curr Genomics.* 2008;9(8):548-55.
5. Kong P, Christia P, Saxena A, Su Y, and Frangogiannis NG. Lack of specificity of fibroblast-specific protein 1 in cardiac remodeling and fibrosis. *Am J Physiol Heart Circ Physiol.* 2013;305(9):H1363-72.
6. Oka T, Xu J, Kaiser RA, Melendez J, Hambleton M, Sargent MA, Lorts A, Brunskill EW, Dorn GW, Conway SJ, et al. Genetic manipulation of periostin expression reveals a role in cardiac hypertrophy and ventricular remodeling. *Circ Res.* 2007;101(3):313-21.
7. Shinde AV, Humeres C, and Frangogiannis NG. The role of α-smooth muscle actin in fibroblast-mediated matrix contraction and remodeling. *Biochim Biophys Acta Mol Basis Dis.* 2017;1863(1):298-309.
8. Christia P, Bujak M, Gonzalez-Quesada C, Chen W, Dobaczewski M, Reddy A, and Frangogiannis NG. Systematic characterization of myocardial inflammation, repair, and remodeling in a mouse model of reperfused myocardial infarction. *J Histochem Cytochem.* 2013;61(8):555-70.
9. Hanna A, Shinde AV, and Frangogiannis NG. Validation of diagnostic criteria and histopathological characterization of cardiac rupture in the mouse model of nonreperfused myocardial infarction. *Am J Physiol Heart Circ Physiol.* 2020;319(5):H948-H64.
10. Lindsey ML, Bolli R, Canty JM, Jr., Du XJ, Frangogiannis NG, Frantz S, Gourdie RG, Holmes JW, Jones SP, Kloner RA, et al. Guidelines for experimental models of myocardial ischemia and infarction. *Am J Physiol Heart Circ Physiol.* 2018;314(4):H812-H38.
11. Hamilton TG, Klinghoffer RA, Corrin PD, and Soriano P. Evolutionary divergence of platelet-derived growth factor alpha receptor signaling mechanisms. *Mol Cell Biol.* 2003;23(11):4013-25.
12. Asli NS, Xaymardan M, Forte E, Waardenberg AJ, Cornwell J, Janbandhu V, Kesteven S, Chandrakanthan V, Malinowska H, Reinhard H, et al. PDGFRα signaling in cardiac fibroblasts modulates quiescence, metabolism and self-renewal, and promotes anatomical and functional repair. *bioRxiv.* 2018:225979.
13. Pinto AR, Ilinykh A, Ivey MJ, Kuwabara JT, D'Antoni ML, Debuque R, Chandran A, Wang L, Arora K, Rosenthal NA, et al. Revisiting Cardiac Cellular Composition. *Circ Res.* 2016;118(3):400-9.
14. Frangogiannis NG, Dewald O, Xia Y, Ren G, Haudek S, Leucker T, Kraemer D, Taffet G, Rollins BJ, and Entman ML. Critical role of monocyte chemoattractant protein-1/CC chemokine ligand 2 in the pathogenesis of ischemic cardiomyopathy. *Circulation.* 2007;115(5):584-92.
15. Saxena A, Chen W, Su Y, Rai V, Uche OU, Li N, and Frangogiannis NG. IL-1 Induces Proinflammatory Leukocyte Infiltration and Regulates Fibroblast Phenotype in the Infarcted Myocardium. *J Immunol.* 2013;191(9):4838-48.
16. Hanna A, Shinde AV, Li R, Alex L, Humeres C, Balasubramanian P, and Frangogiannis NG. Collagen denaturation in the infarcted myocardium involves temporally distinct effects of MT1-MMP-dependent proteolysis and mechanical tension. *Matrix Biol.* 2021;99(18-42.
17. Zitnay JL, Li Y, Qin Z, San BH, Depalle B, Reese SP, Buehler MJ, Yu SM, and Weiss JA. Molecular level detection and localization of mechanical damage in collagen enabled by collagen hybridizing peptides. *Nature communications.* 2017;8(14913.
18. Kong P, Shinde AV, Su Y, Russo I, Chen B, Saxena A, Conway SJ, Graff JM, and Frangogiannis NG. Opposing Actions of Fibroblast and Cardiomyocyte Smad3 Signaling in the Infarcted Myocardium. *Circulation.* 2018;137(7):707-24.

19. Trapnell C, Pachter L, and Salzberg SL. TopHat: discovering splice junctions with RNA-Seq. *Bioinformatics.* 2009;25(9):1105-11.
20. Trapnell C, Williams BA, Pertea G, Mortazavi A, Kwan G, van Baren MJ, Salzberg SL, Wold BJ, and Pachter L. Transcript assembly and quantification by RNA-Seq reveals unannotated transcripts and isoform switching during cell differentiation. *Nat Biotechnol.* 2010;28(5):511-5.
21. Anders S, Pyl PT, and Huber W. HTSeq--a Python framework to work with high-throughput sequencing data. *Bioinformatics.* 2015;31(2):166-9.
22. Anders S, and Huber W. Differential expression analysis for sequence count data. *Genome Biol.* 2010;11(10):R106.
23. Yu G, Wang LG, Han Y, and He QY. clusterProfiler: an R package for comparing biological themes among gene clusters. *OMICS.* 2012;16(5):284-7.

Smad7 effects on TGF-β and Erbb2 restrain myofibroblast activation, and protect from post-infarction heart failure

Claudio Humeres, Arti V Shinde, Anis Hanna, Linda Alex, Silvia C Hernández, Ruoshui Li, Bijun Chen, Simon J Conway and Nikolaos G Frangogiannis.

Full Unedited/Uncropped blots JCI R2
Blots for main figures

Full unedited gel for FIGURE 6A.

smad7

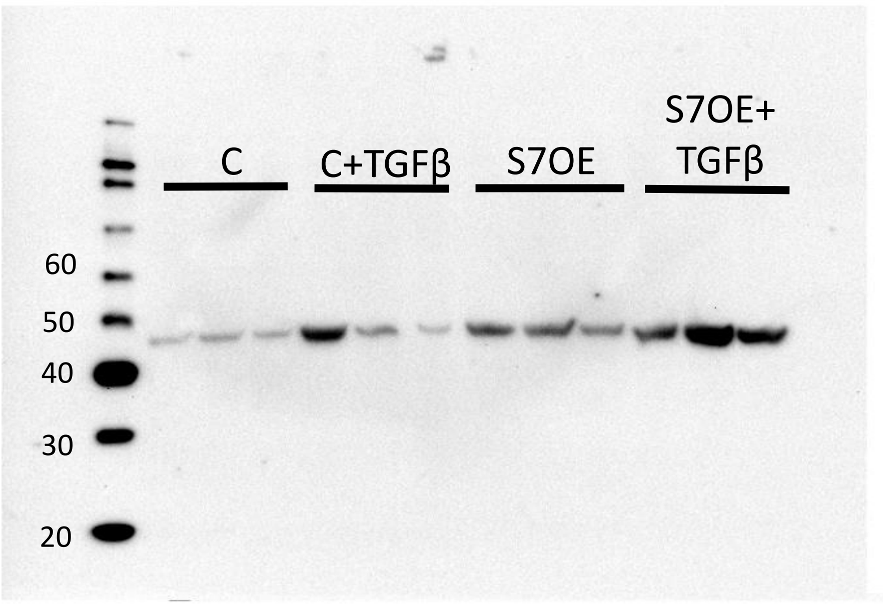

Gapdh for smad7 mb

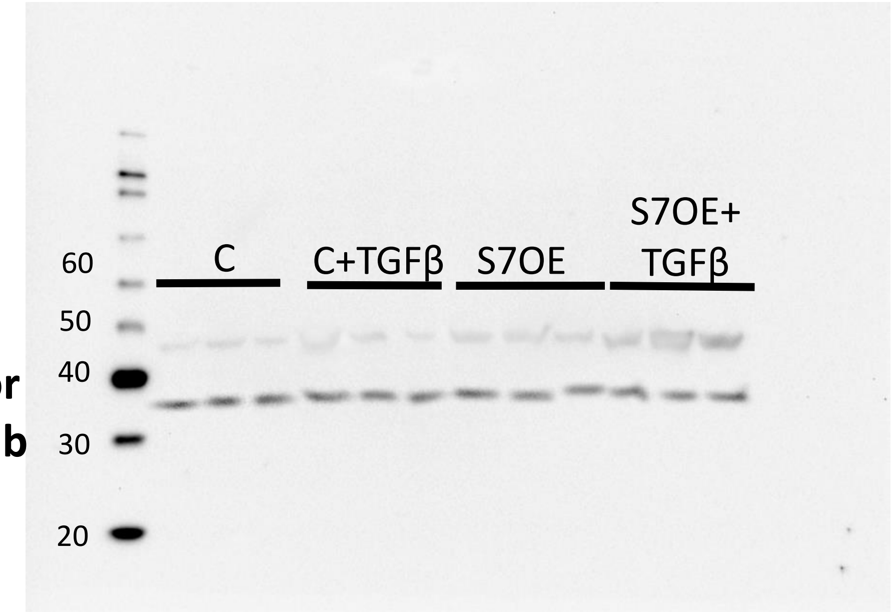

Full unedited gel for FIGURE 6B.

aSMA

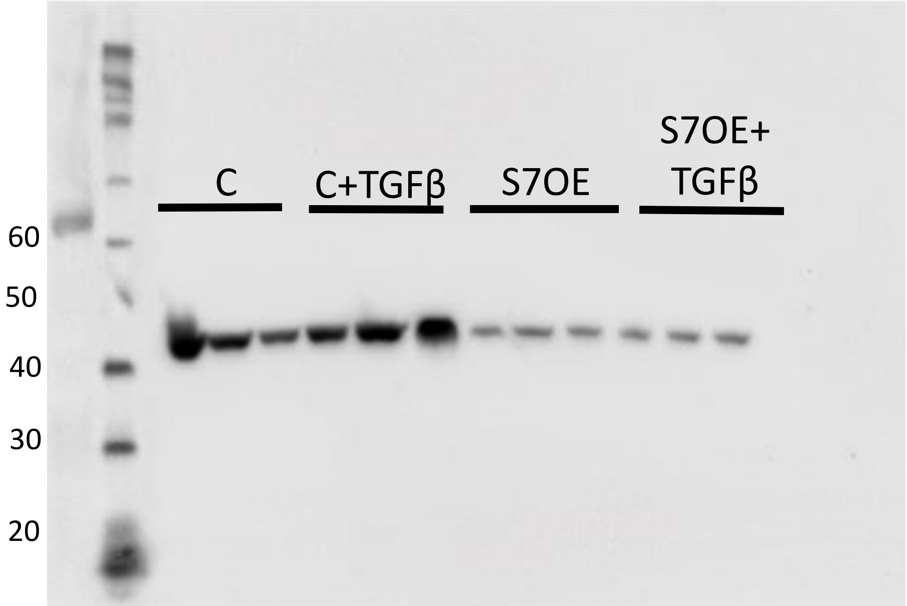

Gapdh for aSMA mb

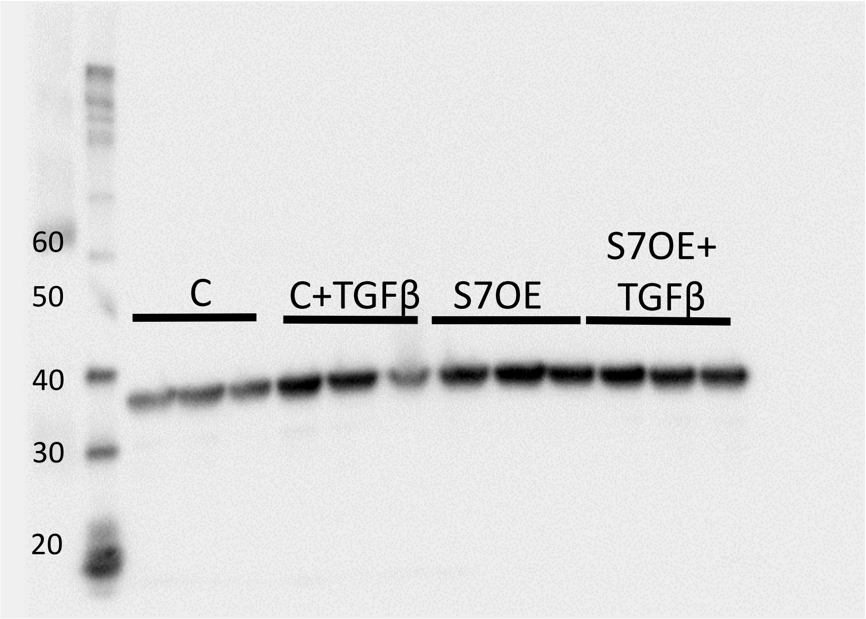

Full unedited gel for FIGURE 7A

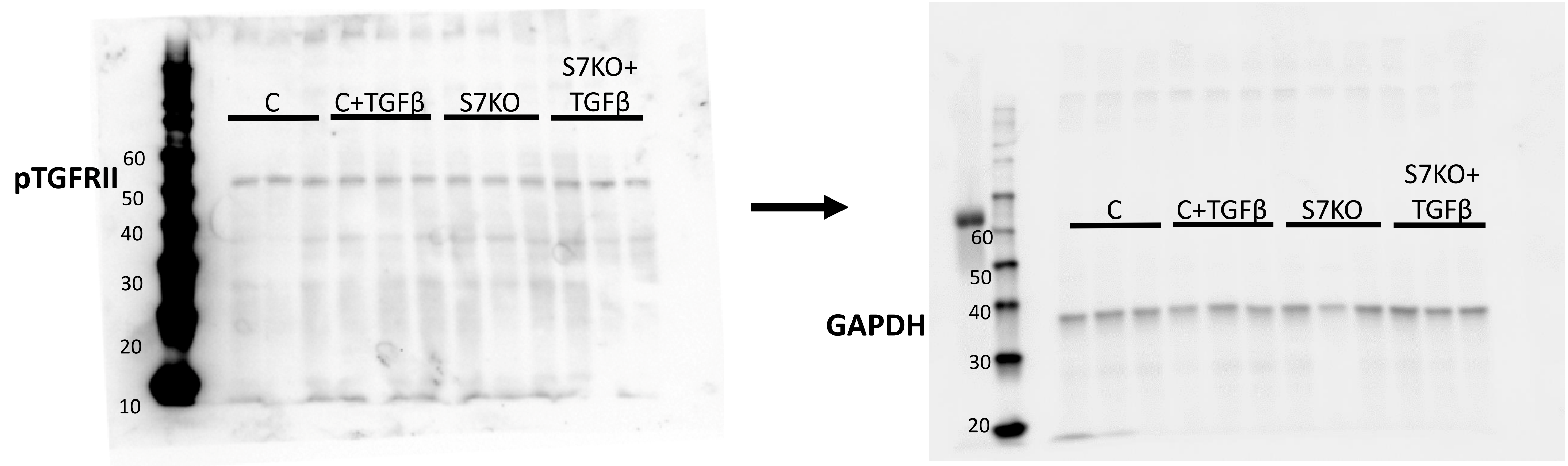

Full unedited gel for FIGURE 7B

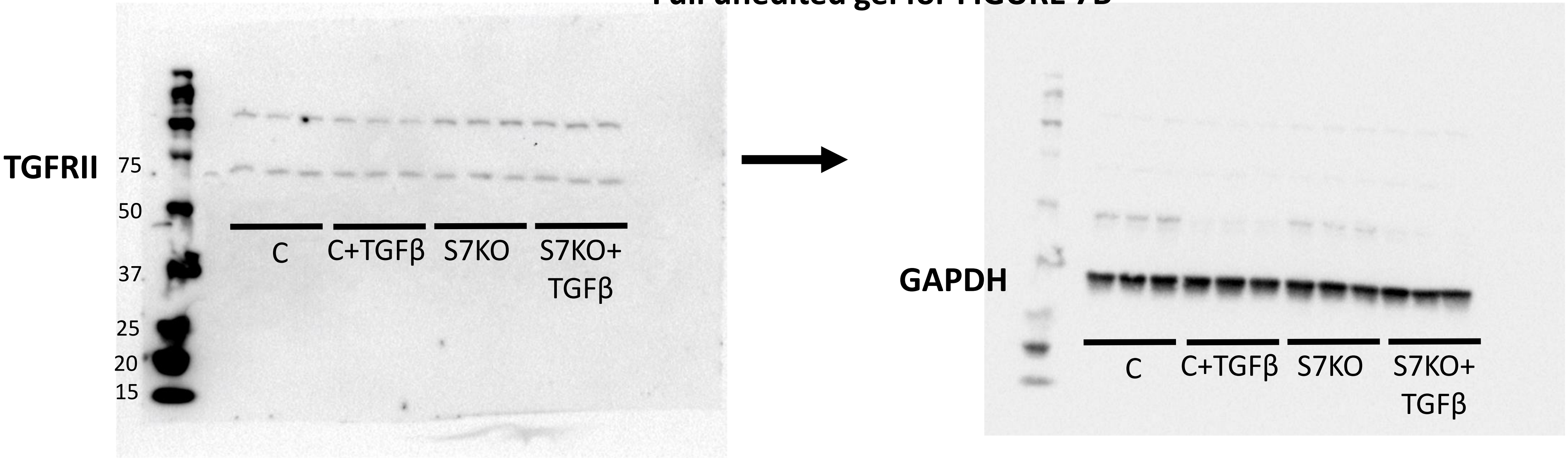

Full unedited gels for FIGURE 7C

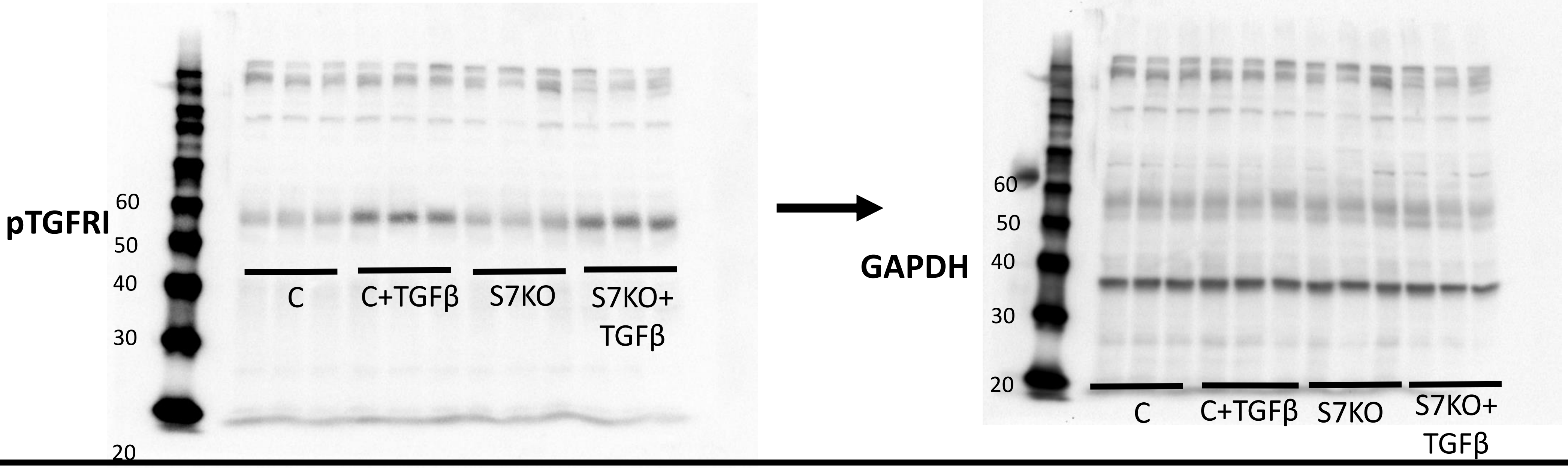

Full unedited gels for FIGURE 7D

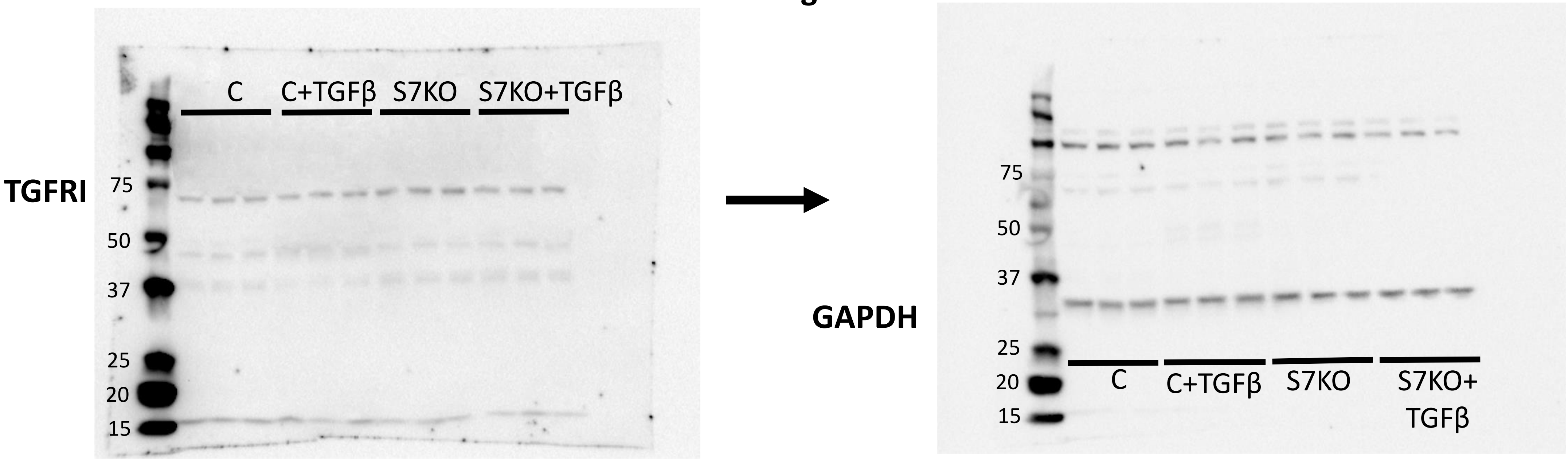

Full unedited figure for FIGURE 7E

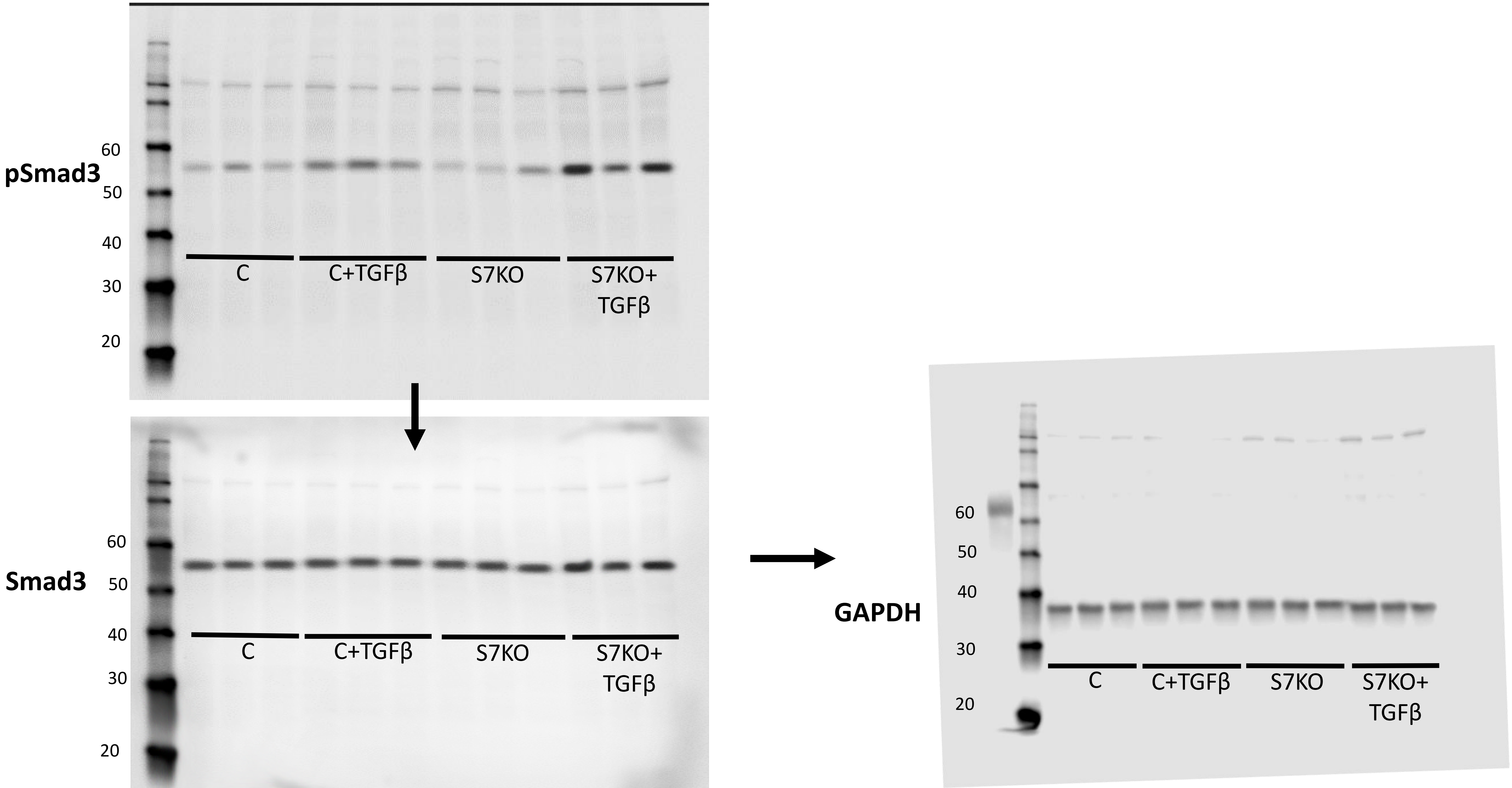

Full unedited blot for FIGURE 7F

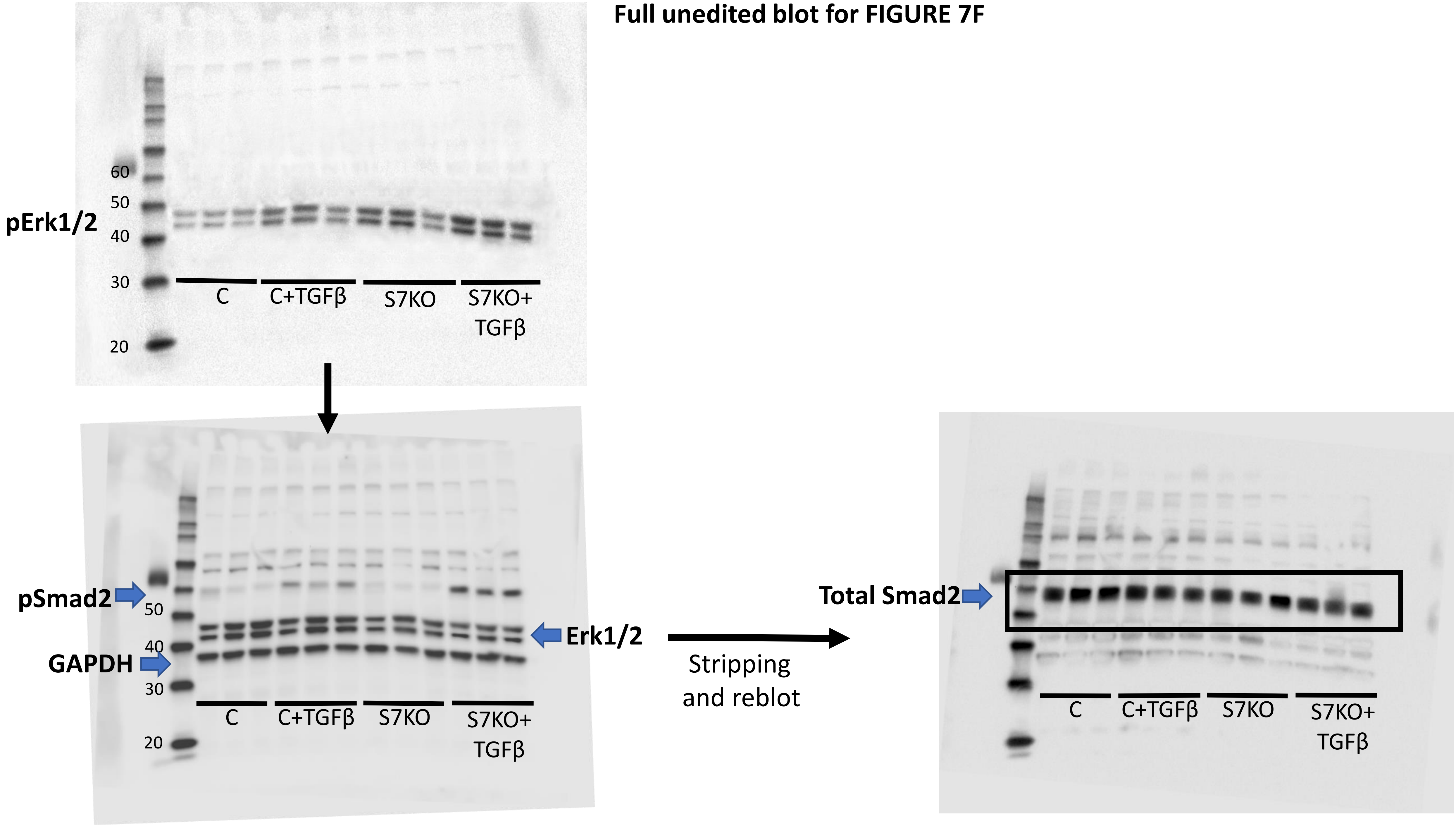

Full unedited blot for FIGURE 7G

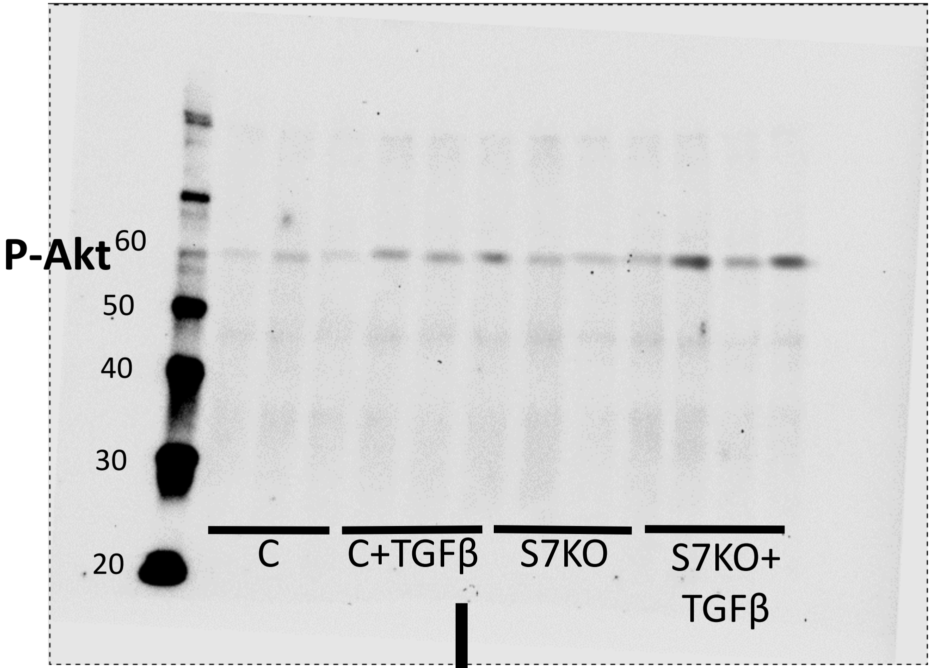

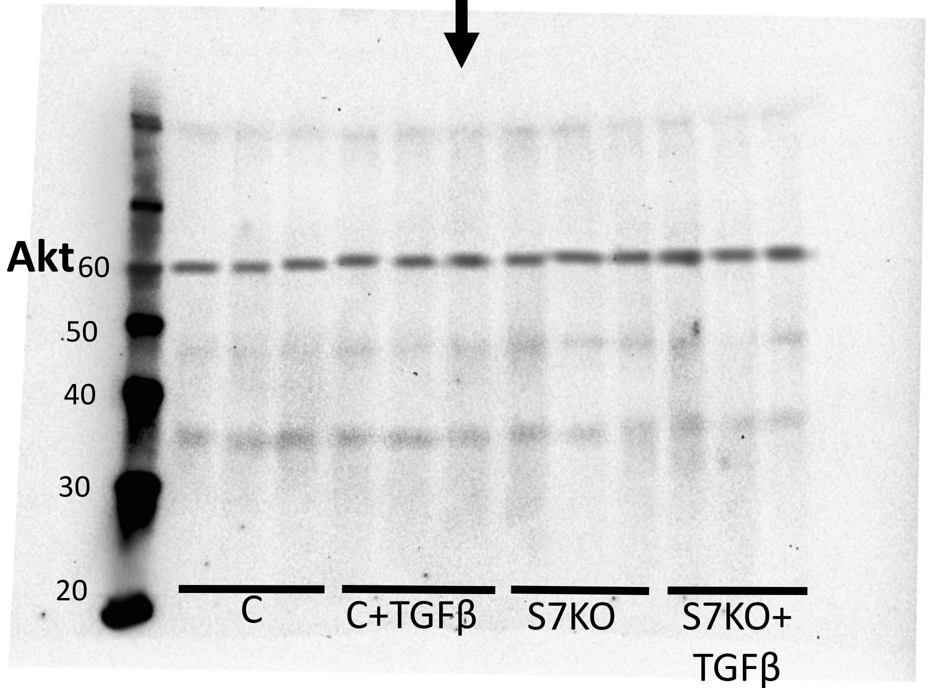

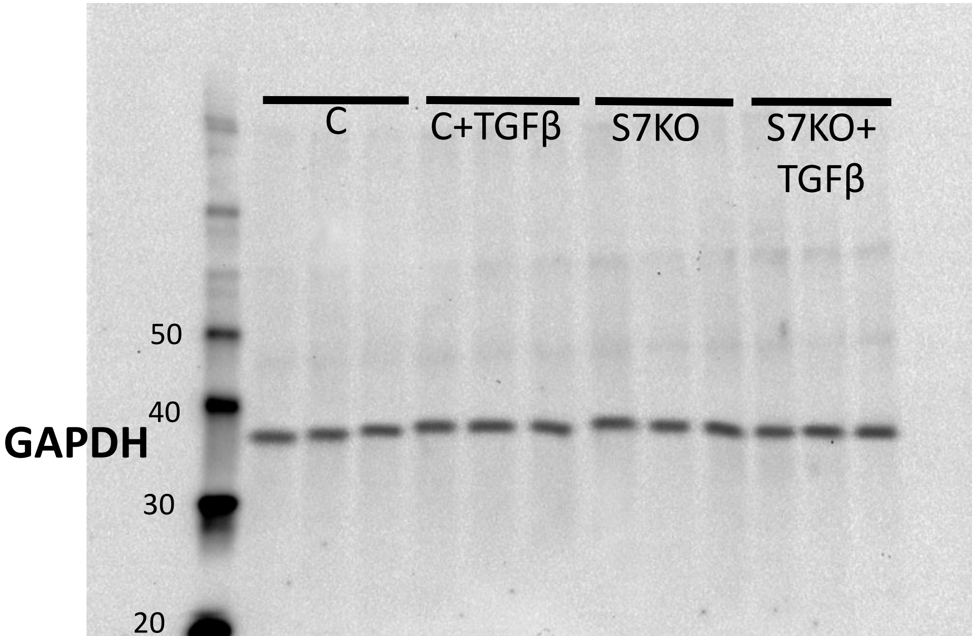

Full unedited blots for FIGURE 9A

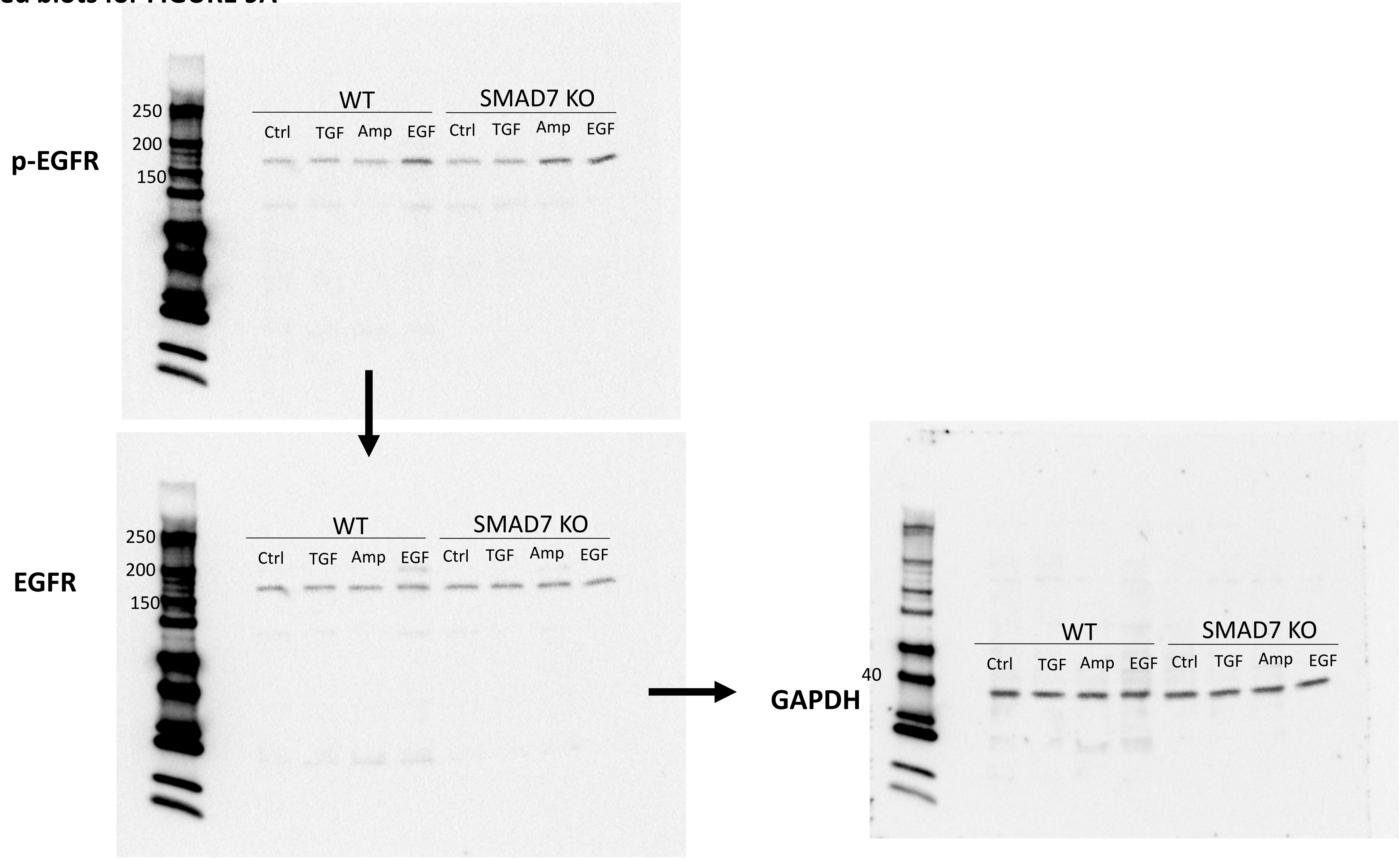

Full unedited blots for FIGURE 9E

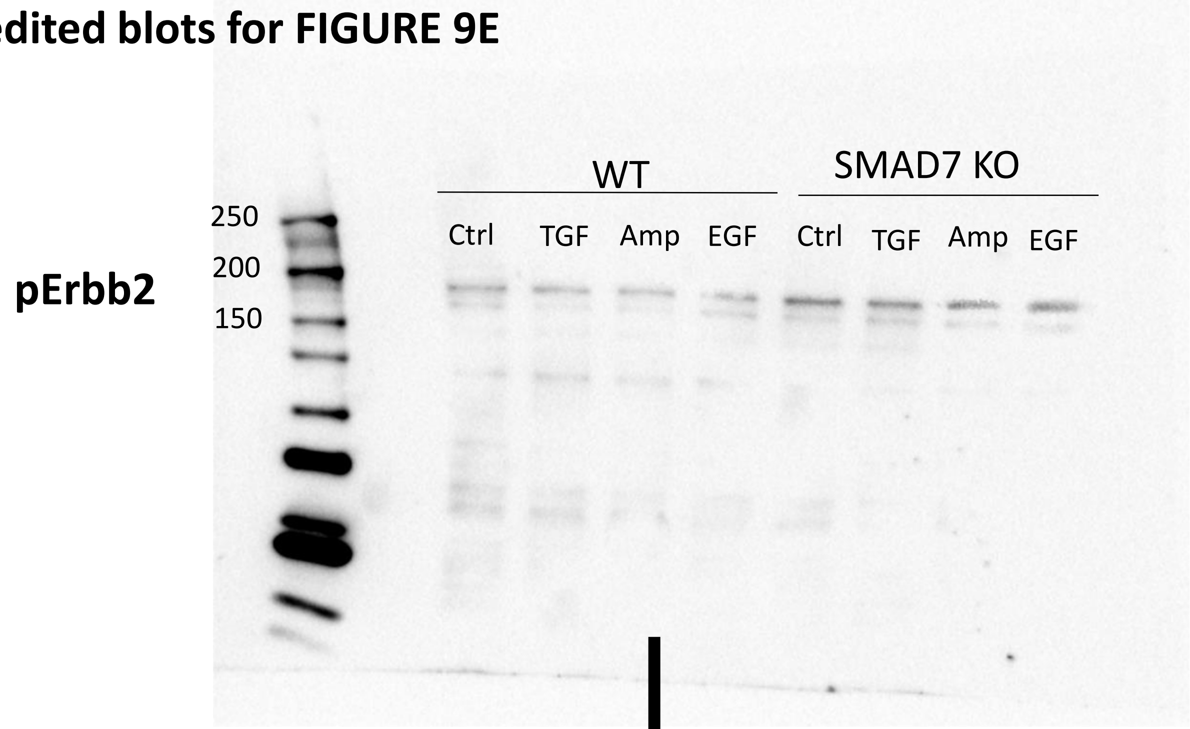

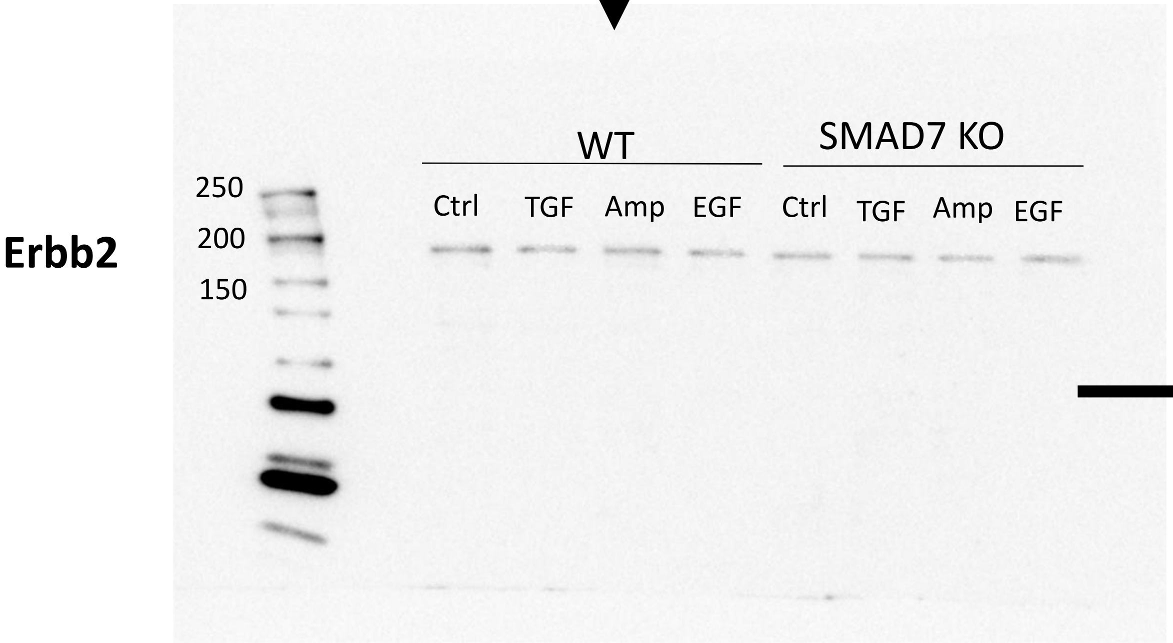

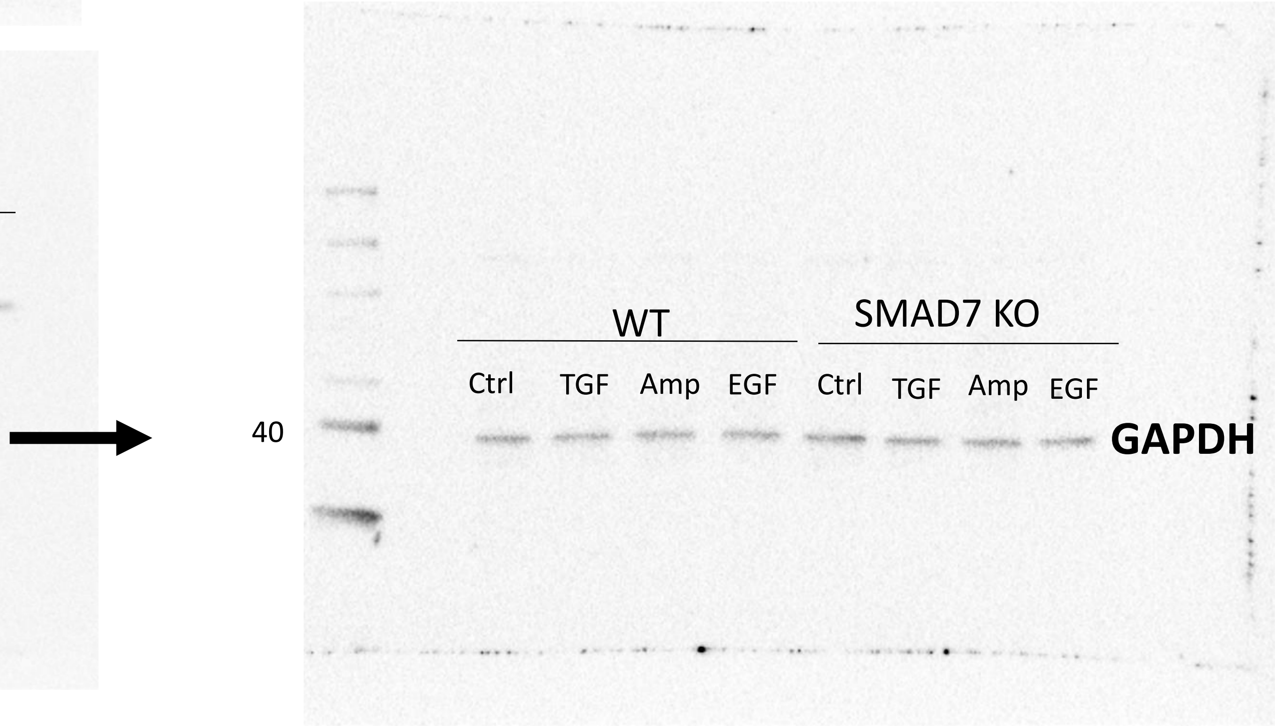

Full unedited blots for FIGURE 9K

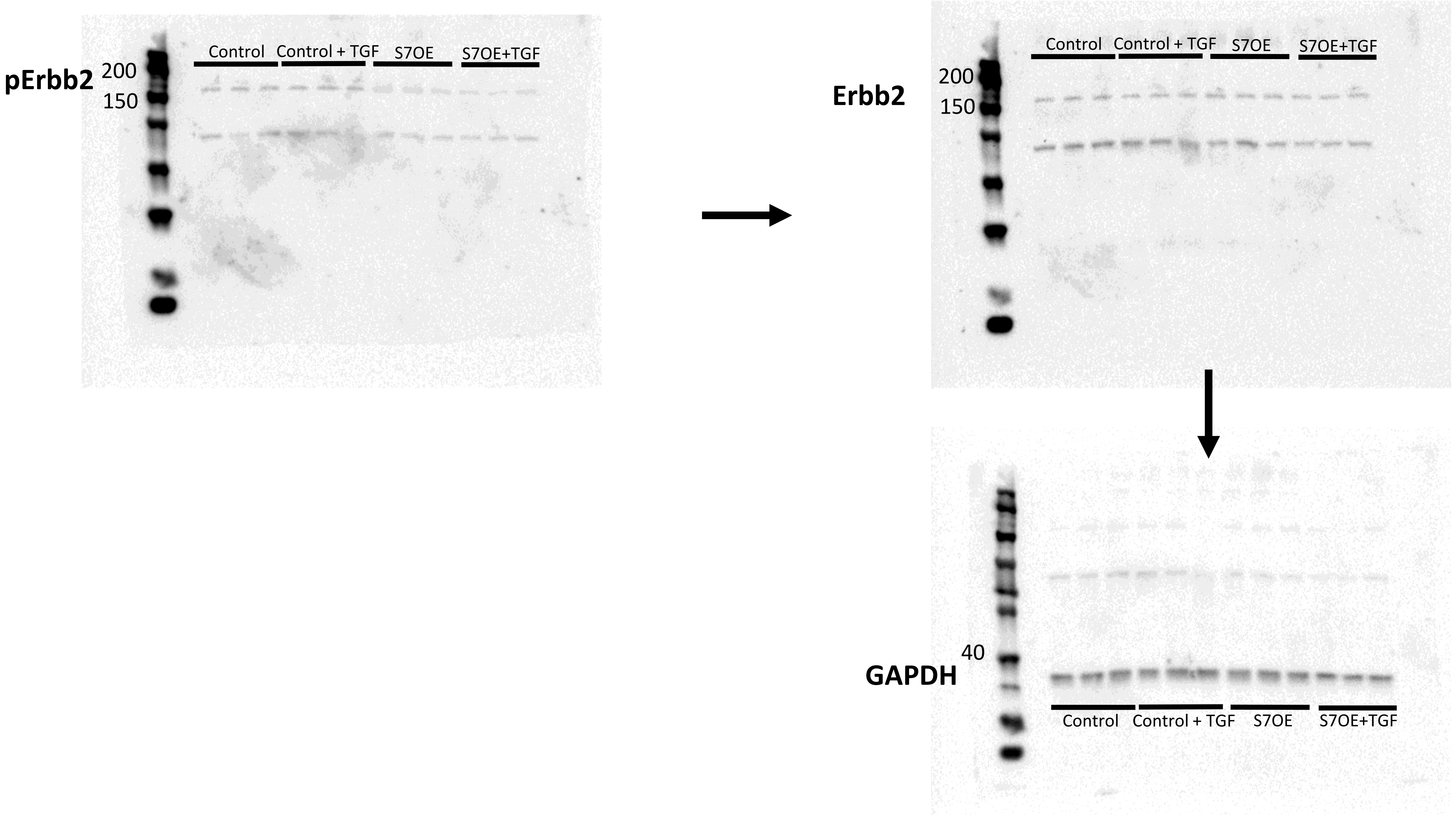

Full unedited blots for FIGURE 11: IP fraction

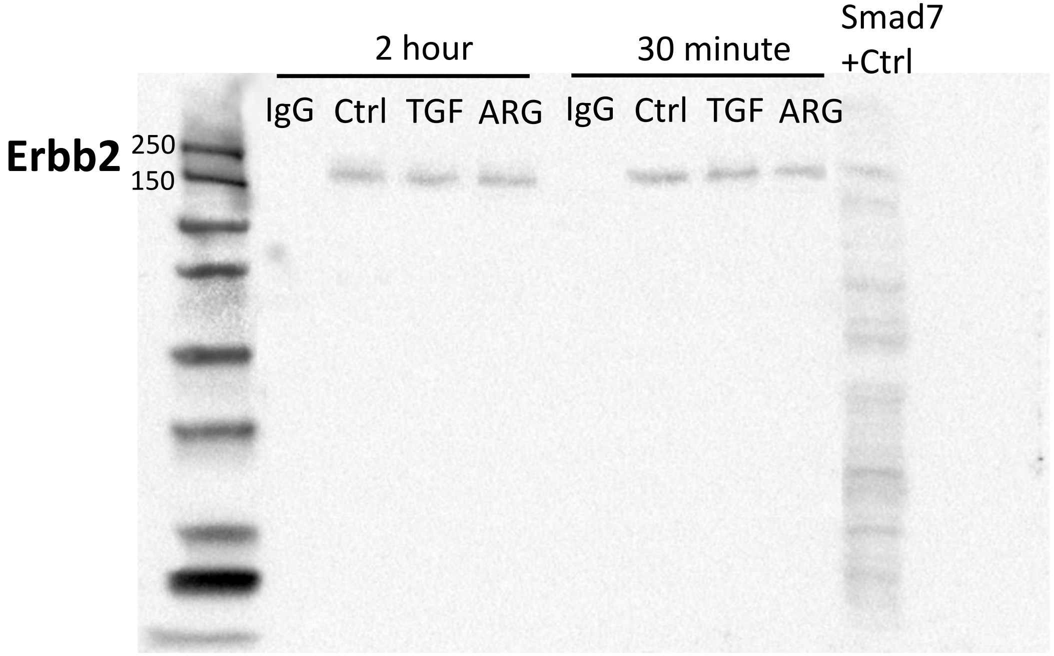

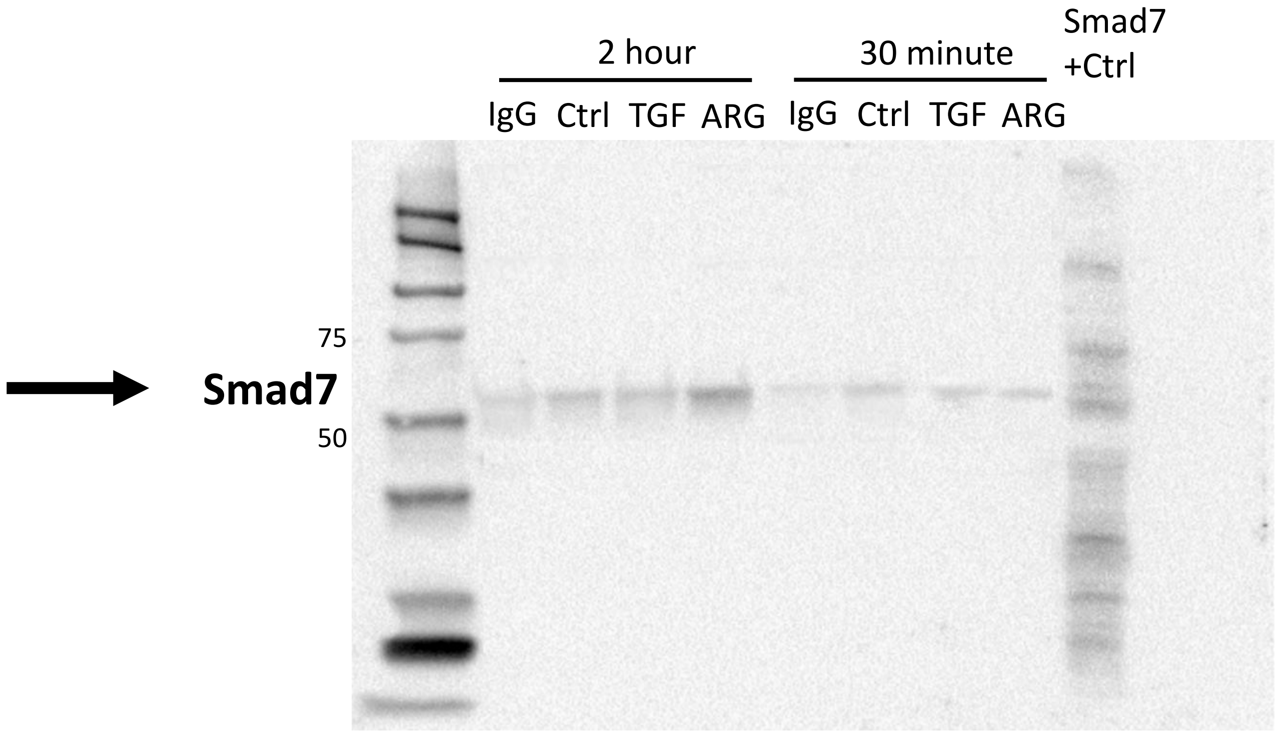

Full unedited blots for FIGURE 11: Whole supernatant (WCL)

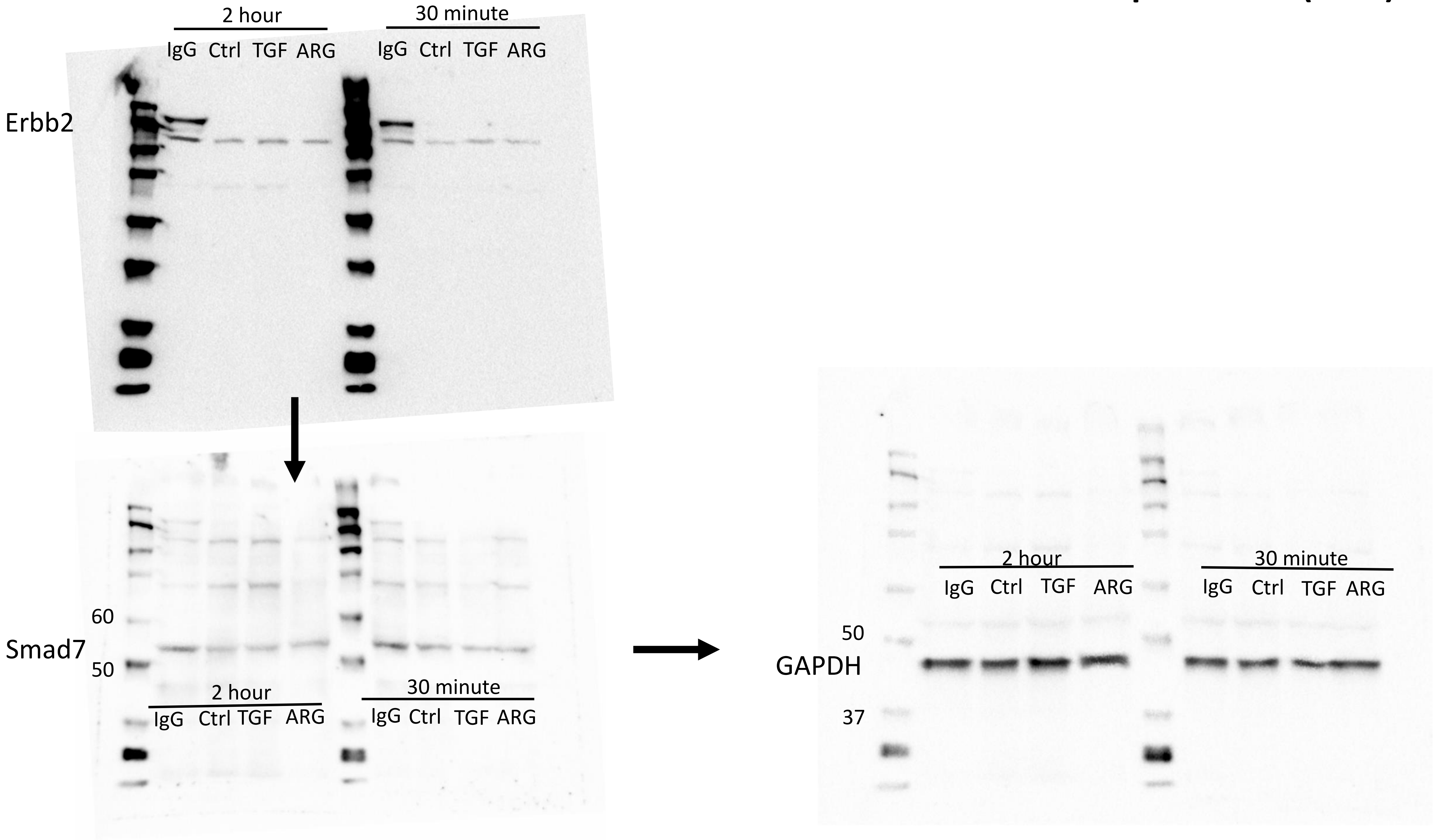

Smad7 effects on TGF-β and Erbb2 restrain myofibroblast activation, and protect from post-infarction heart failure

Claudio Humeres, Arti V Shinde, Anis Hanna, Linda Alex, Silvia C Hernández, Ruoshui Li, Bijun Chen, Simon J Conway and Nikolaos G Frangogiannis.

Full Unedited/Uncropped blots JCI R2
Blots for supplemental figures

Full unedited blots for Supplementary 12C

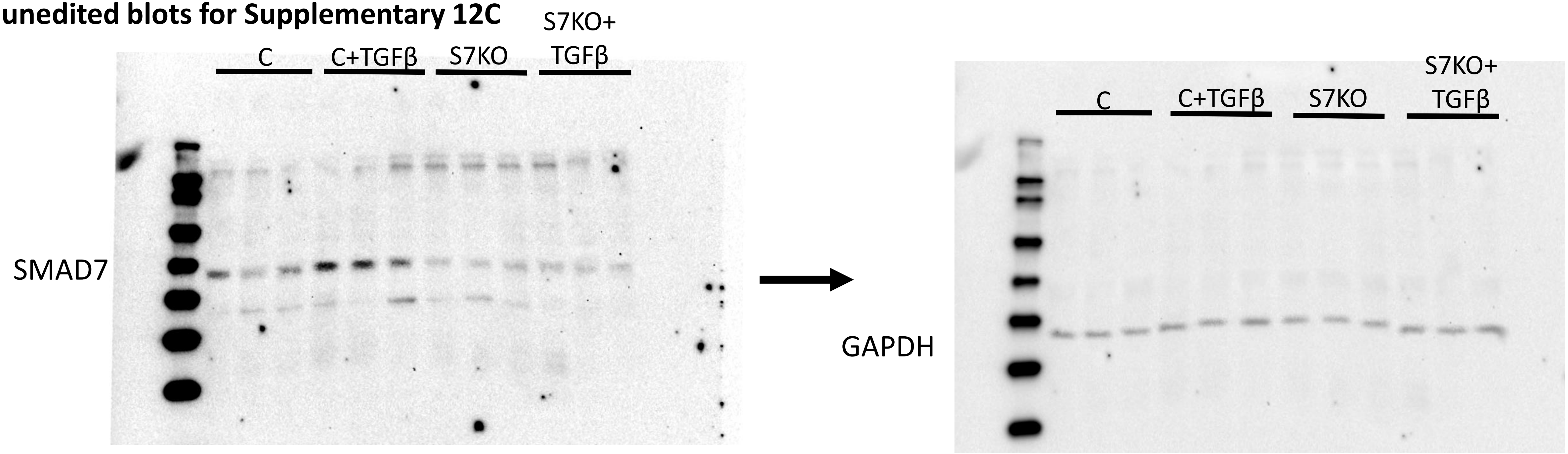

Full unedited blots for Supplementary 12F

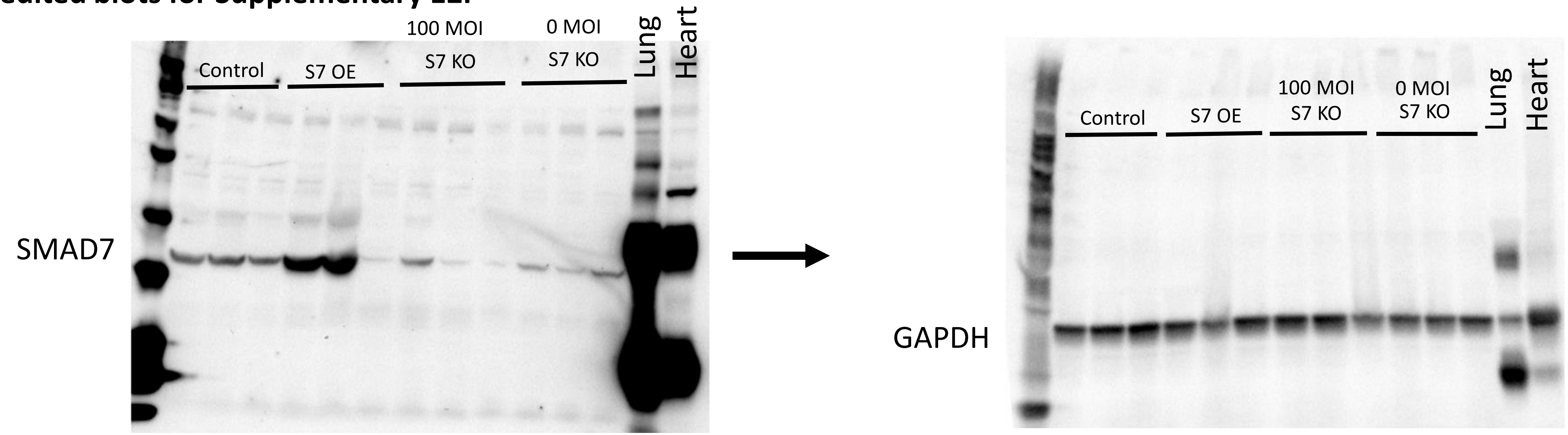

Full unedited blots for SUPPLEMENTARY FIGURE 21A

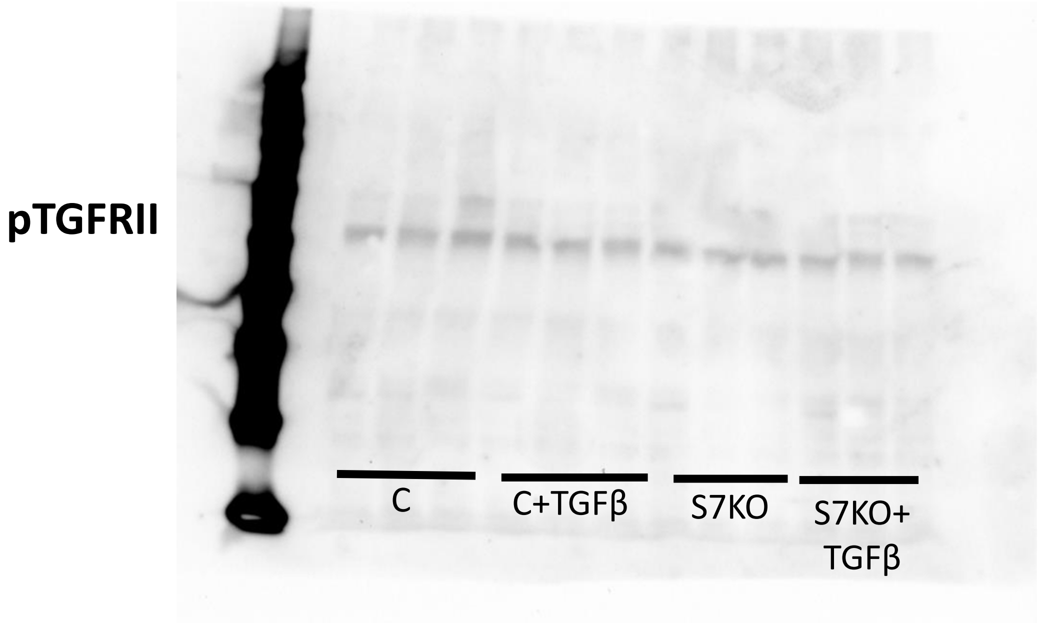

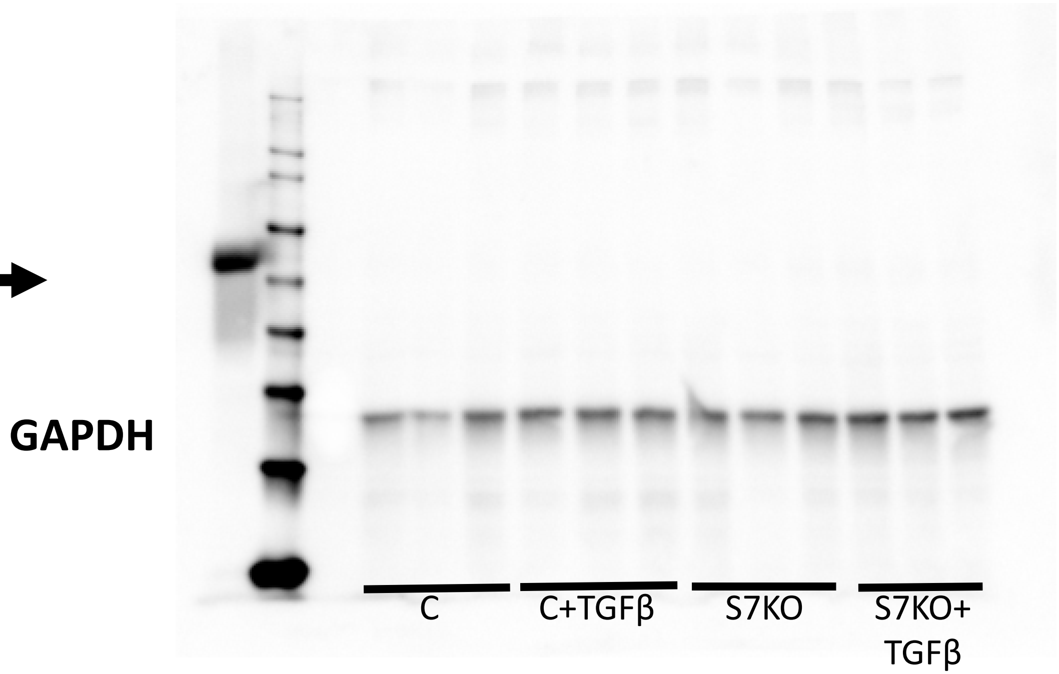

Full unedited blots for SUPPLEMENTARY FIGURE 21B

TGFRII

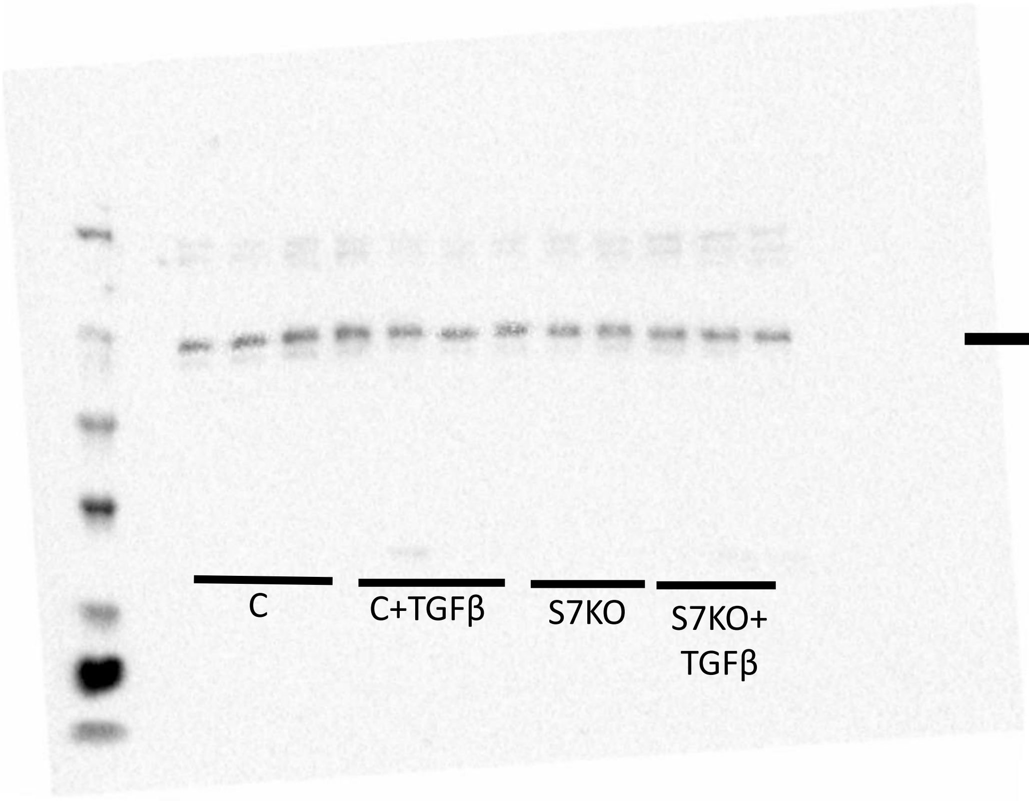

GAPDH

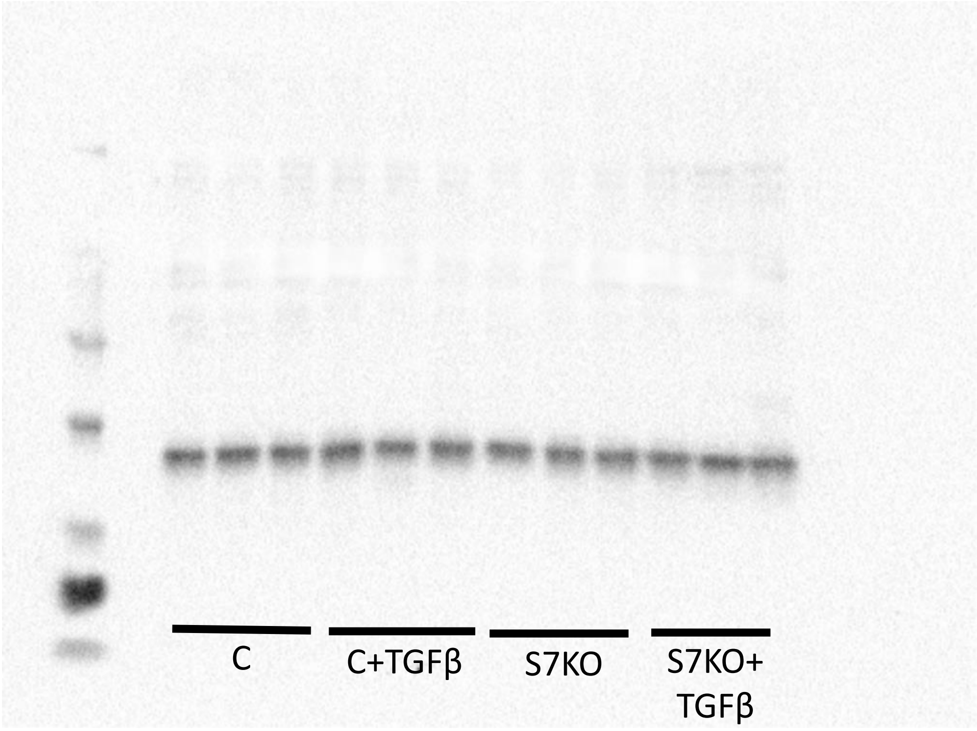

Full unedited blots for SUPPLEMENTARY FIGURE 21C

p-TGFRI

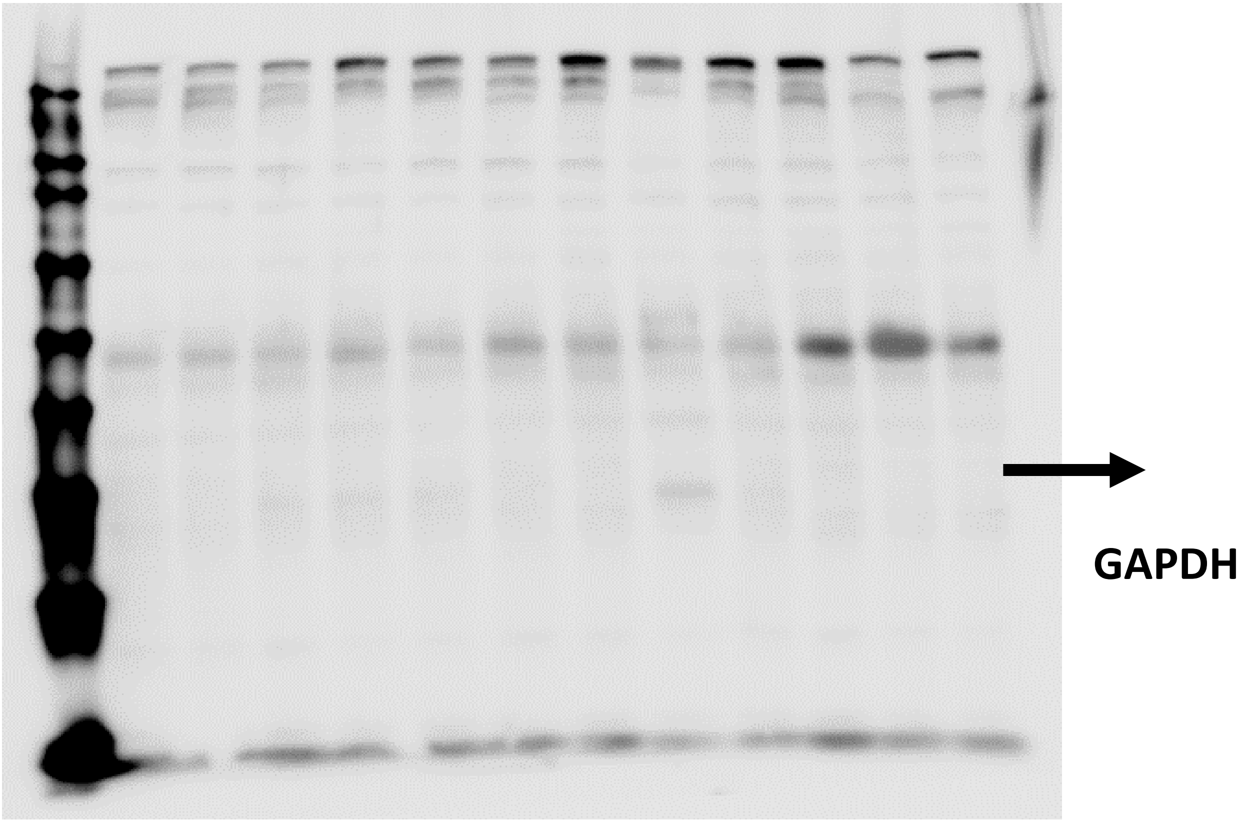

C C+TGFβ S7KO S7KO+ TGFβ

GAPDH

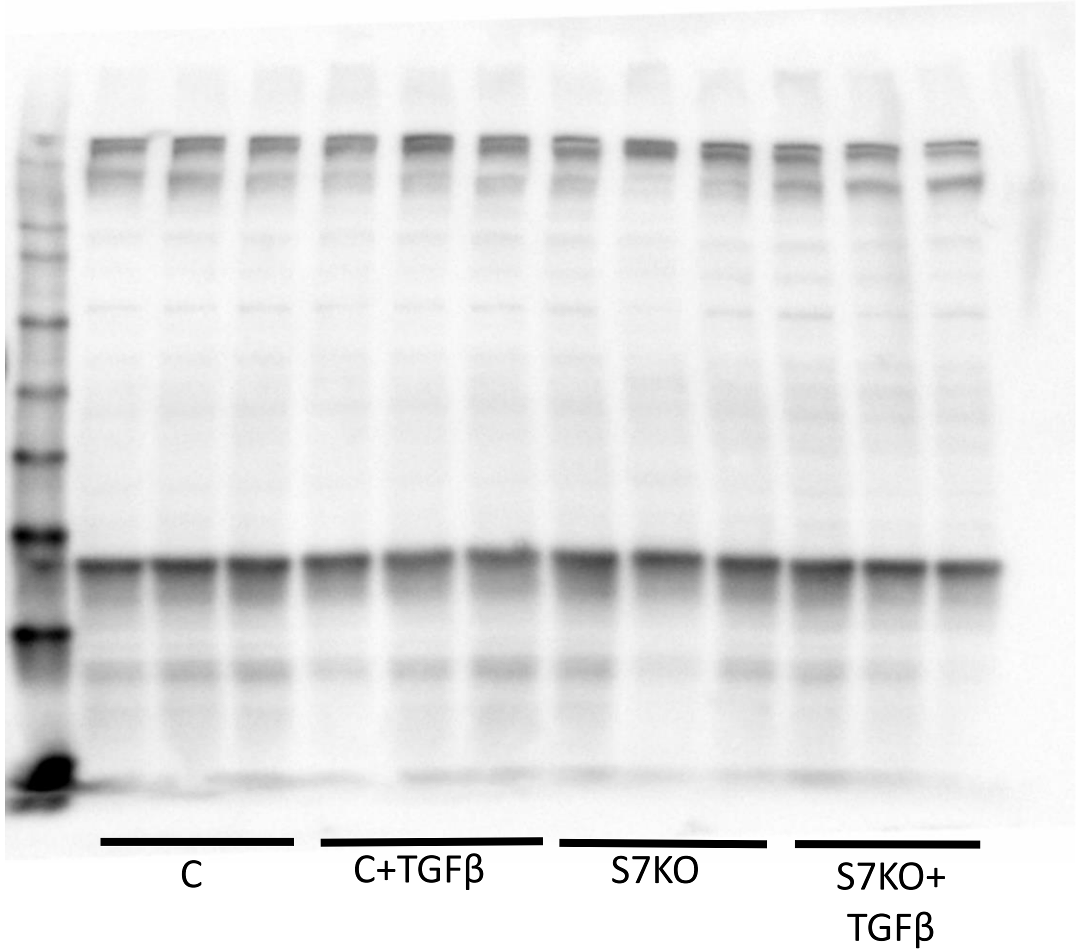

C C+TGFβ S7KO S7KO+ TGFβ

Full unedited blots for SUPPLEMENTARY FIGURE 21D

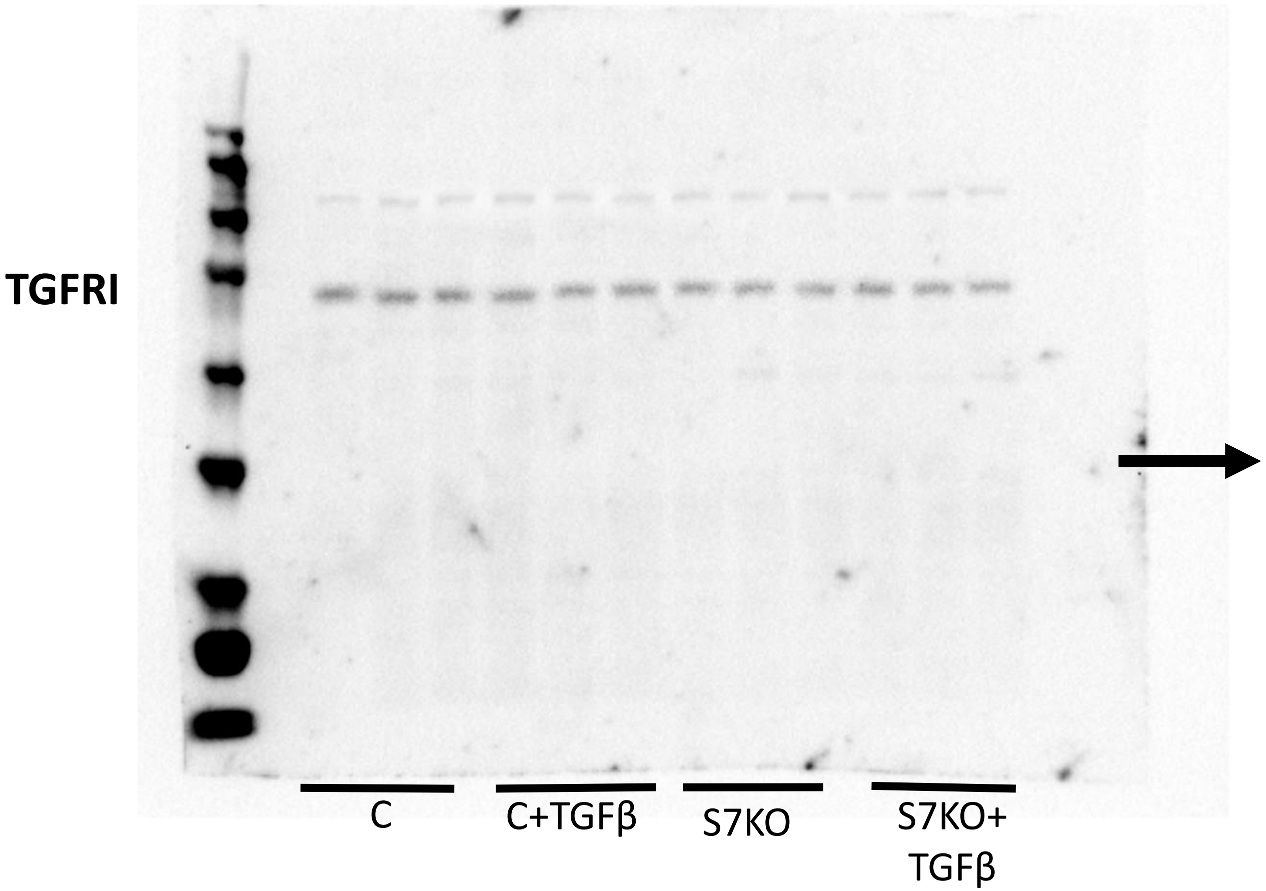

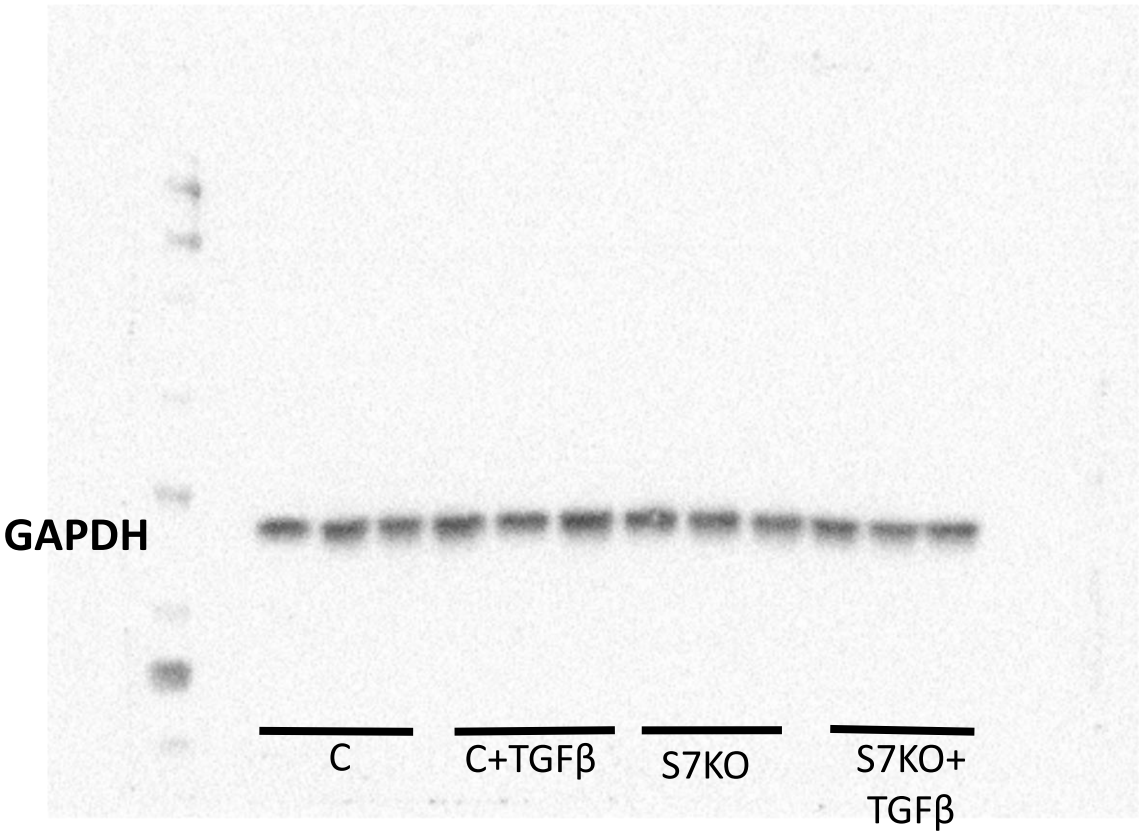

Full unedited blots for SUPPLEMENTARY FIGURE 21E

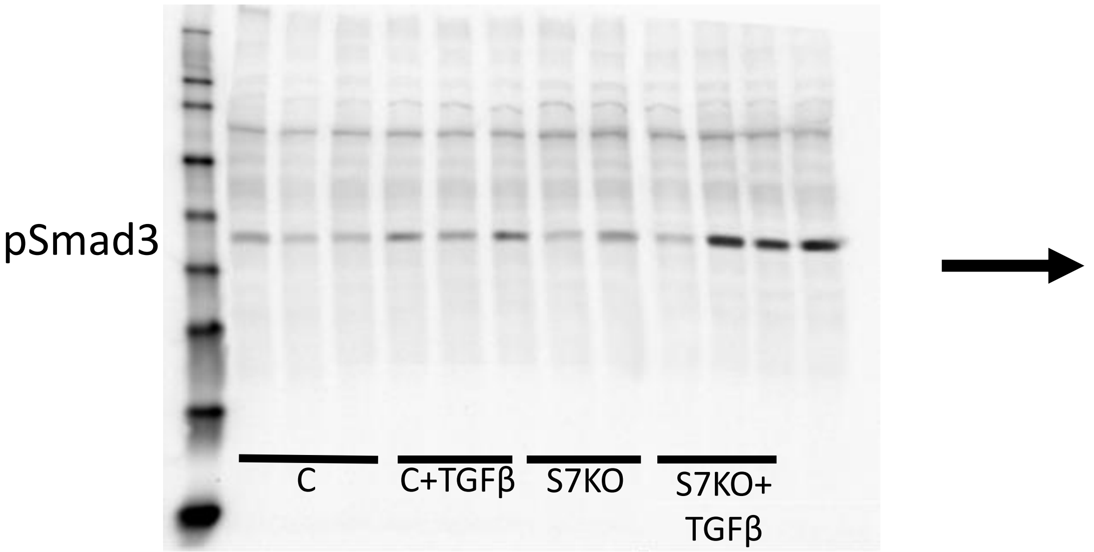

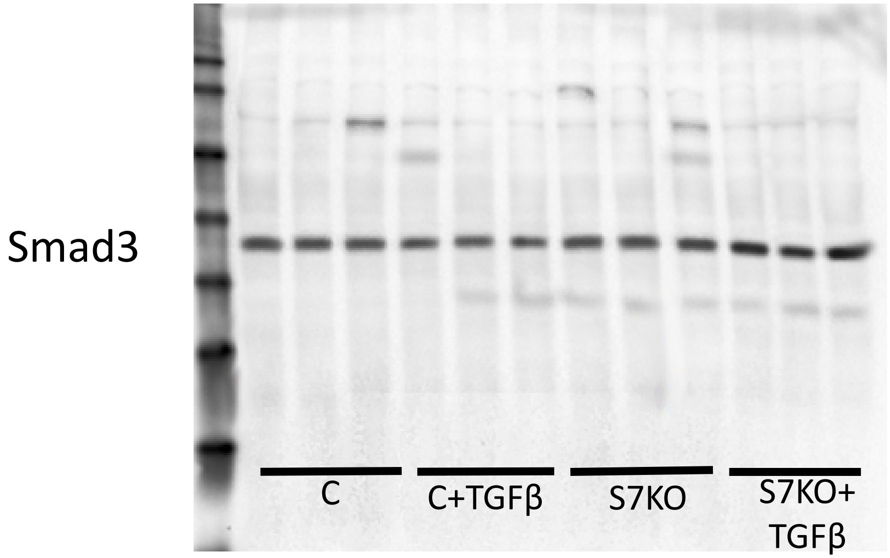

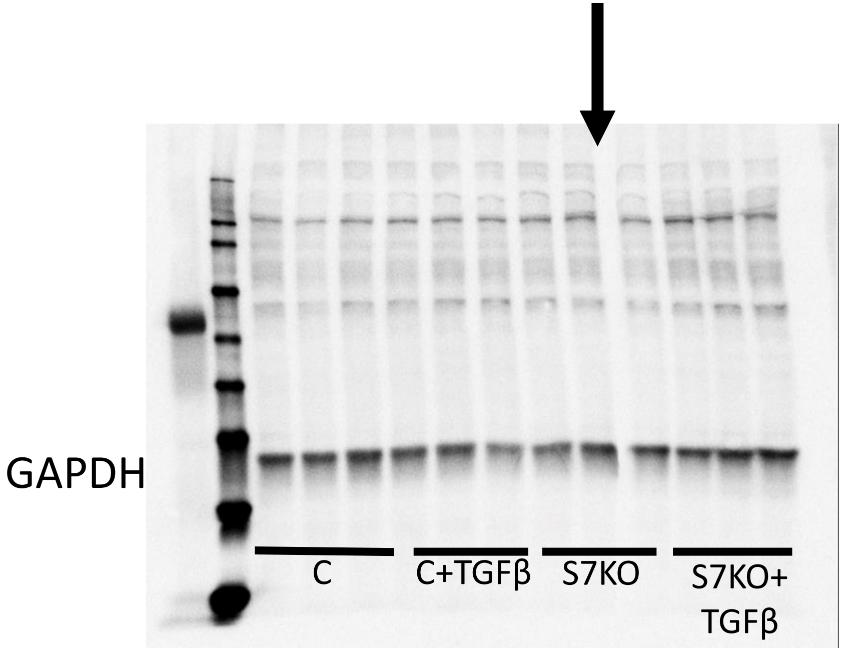

Full unedited blots for SUPPLEMENTARY FIGURE 21F.

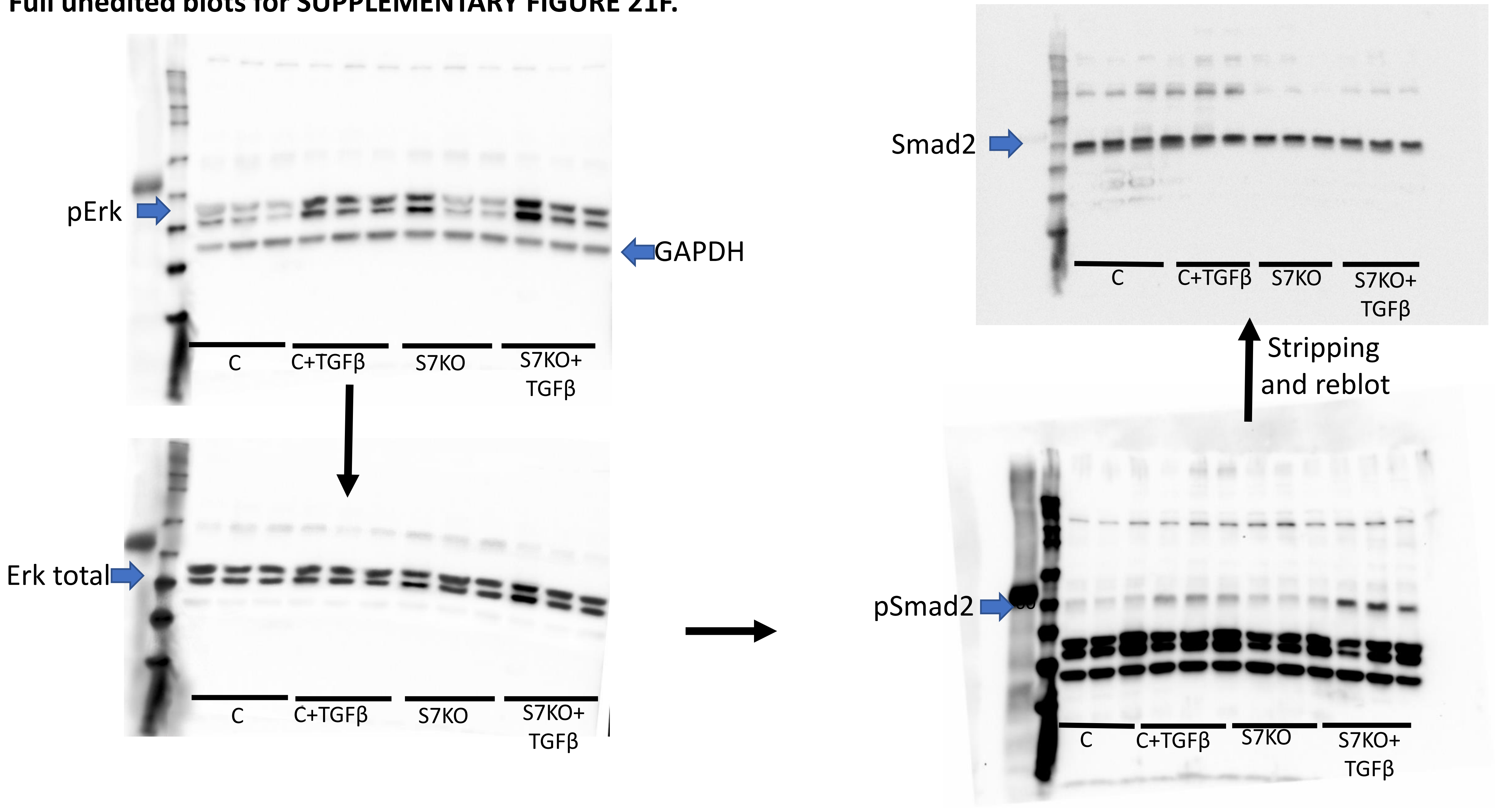

Full unedited blots for SUPPLEMENTARY FIGURE 21G

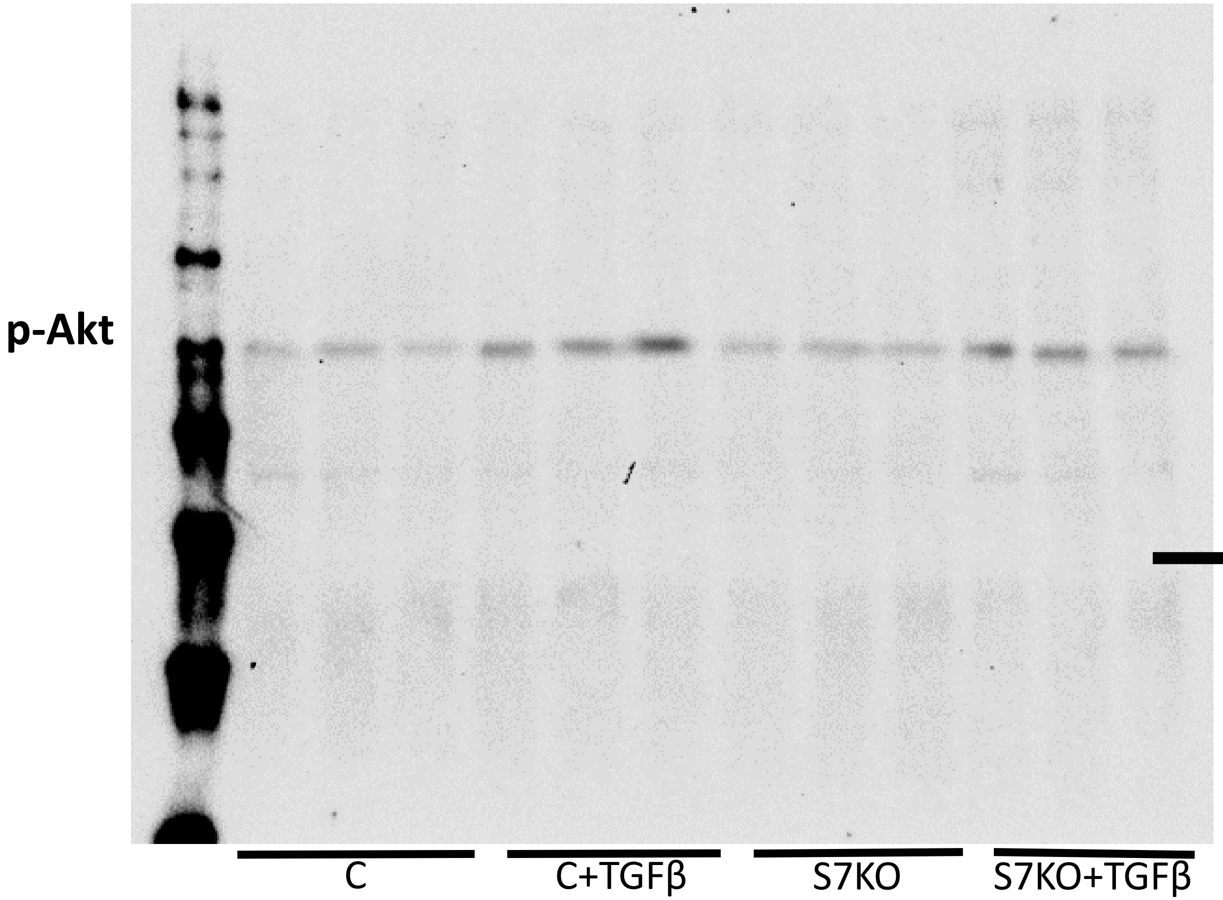

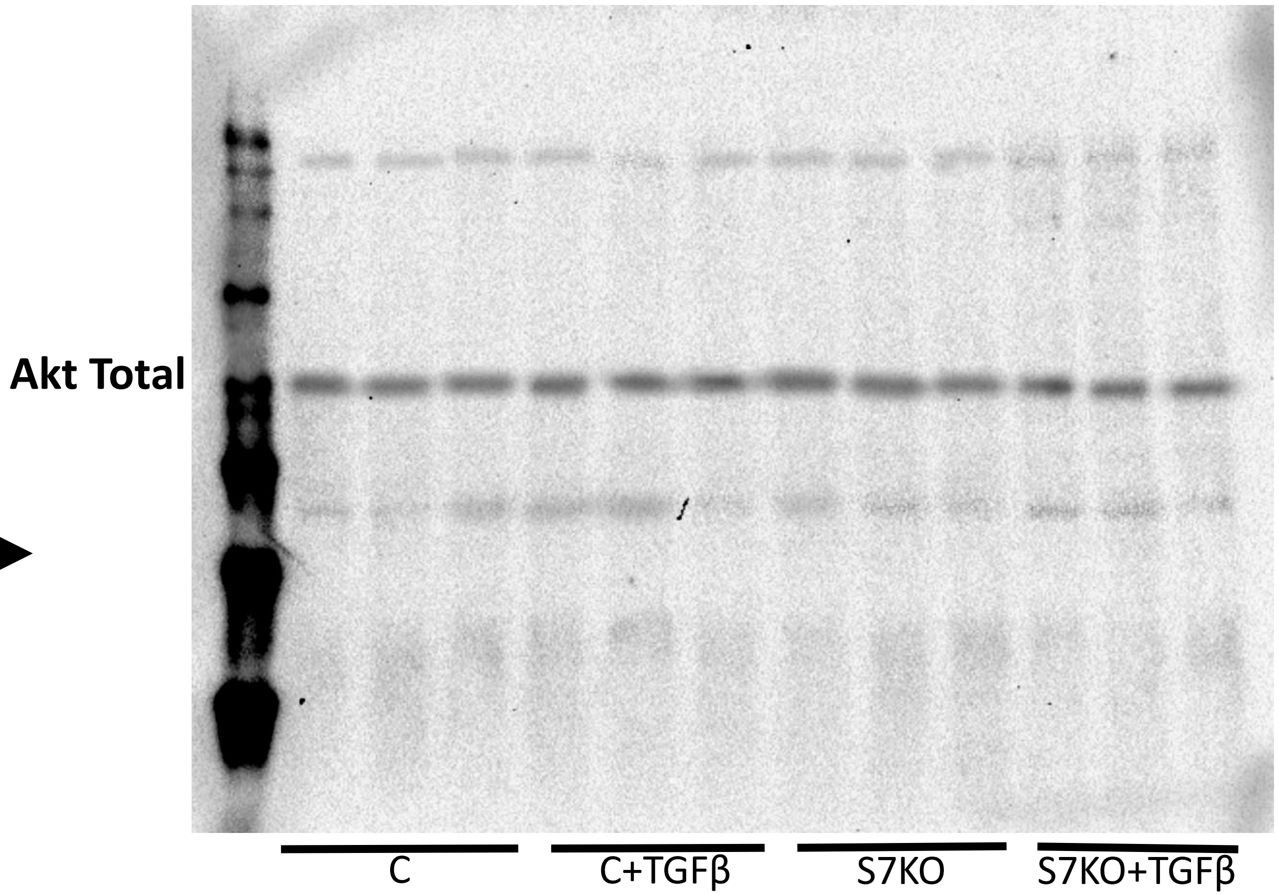

Full unedited blots for SUPPLEMENTARY FIGURE 24A

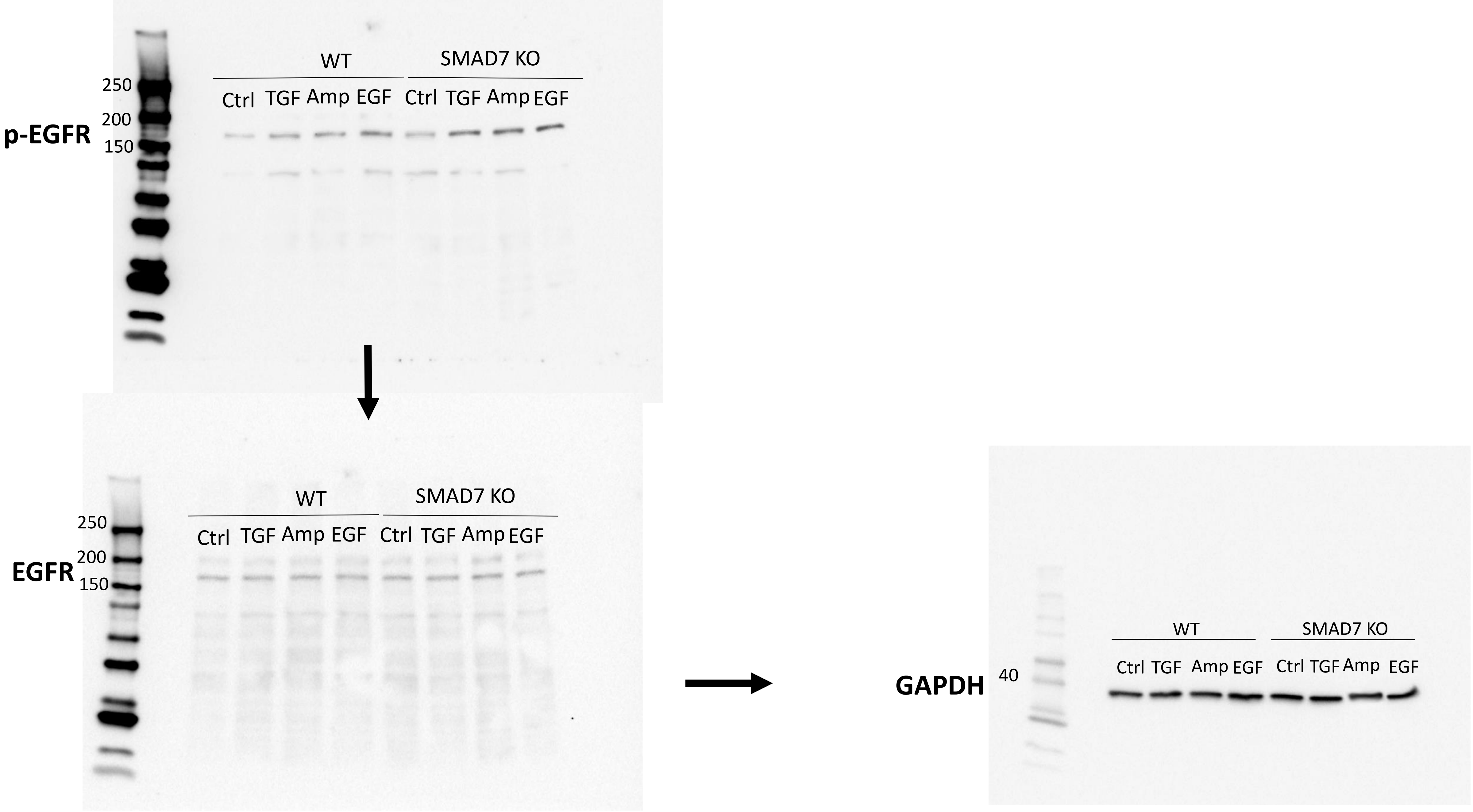

Full unedited blots for SUPPLEMENTARY FIGURE 24E

pErbb2

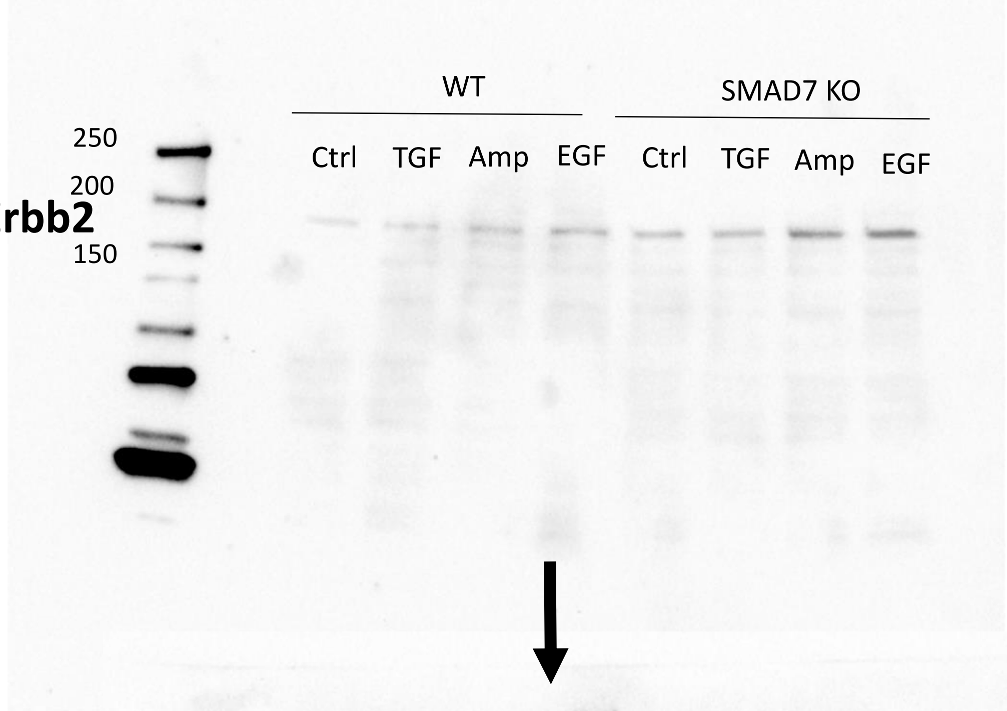

Erbb2

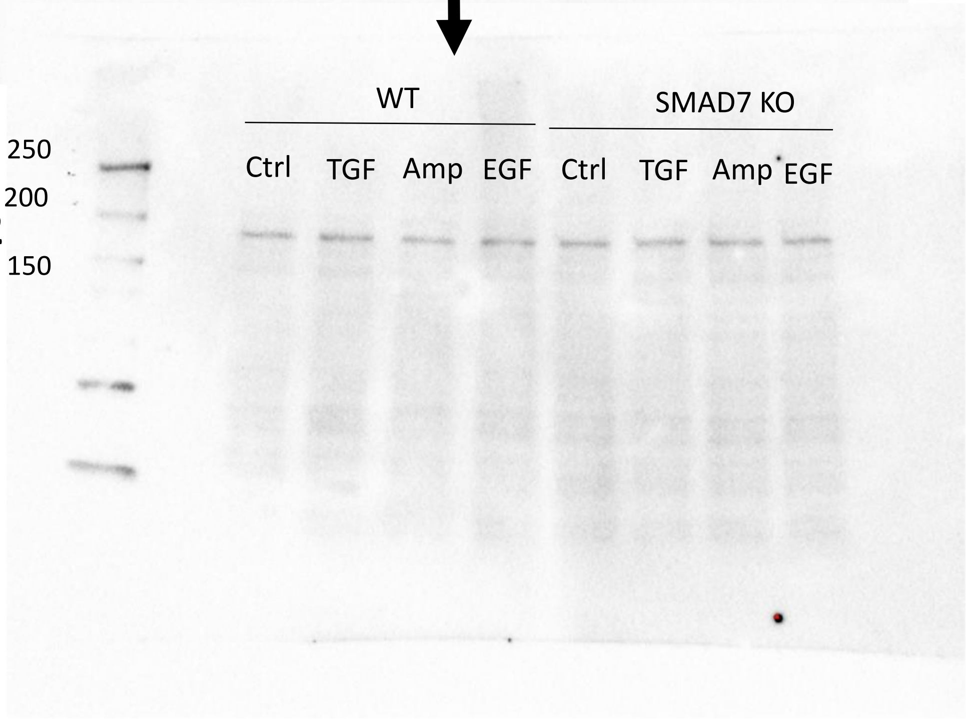

GAPDH

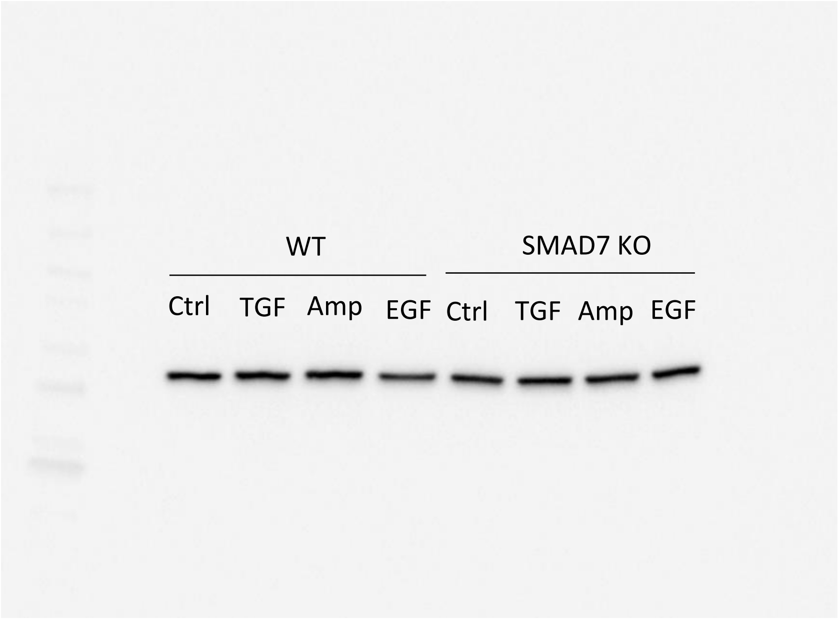

Full unedited blots for SUPPLEMENTARY FIGURE 25A

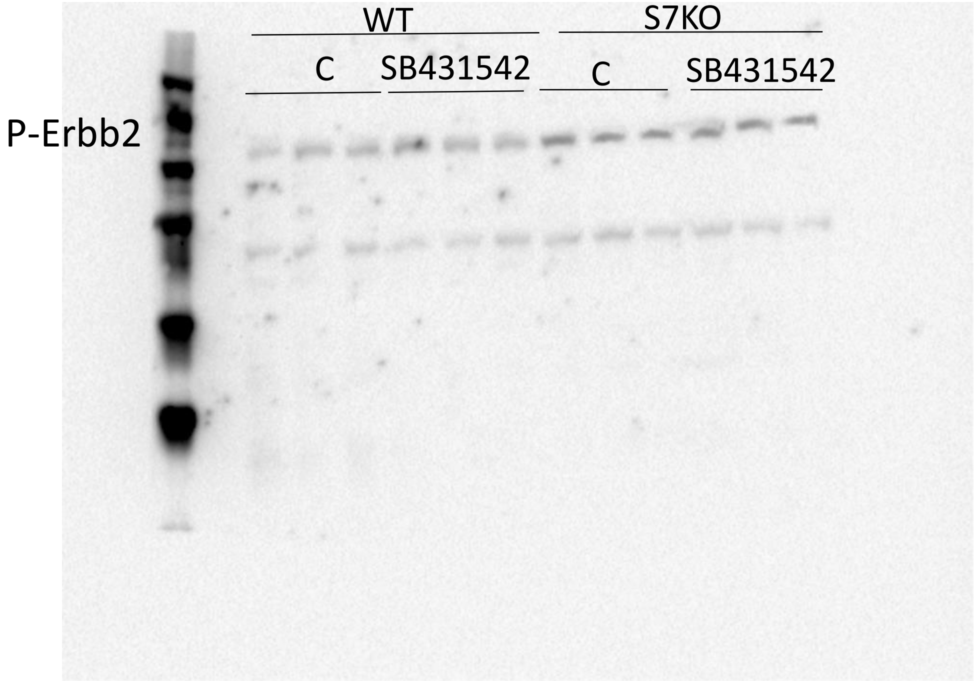

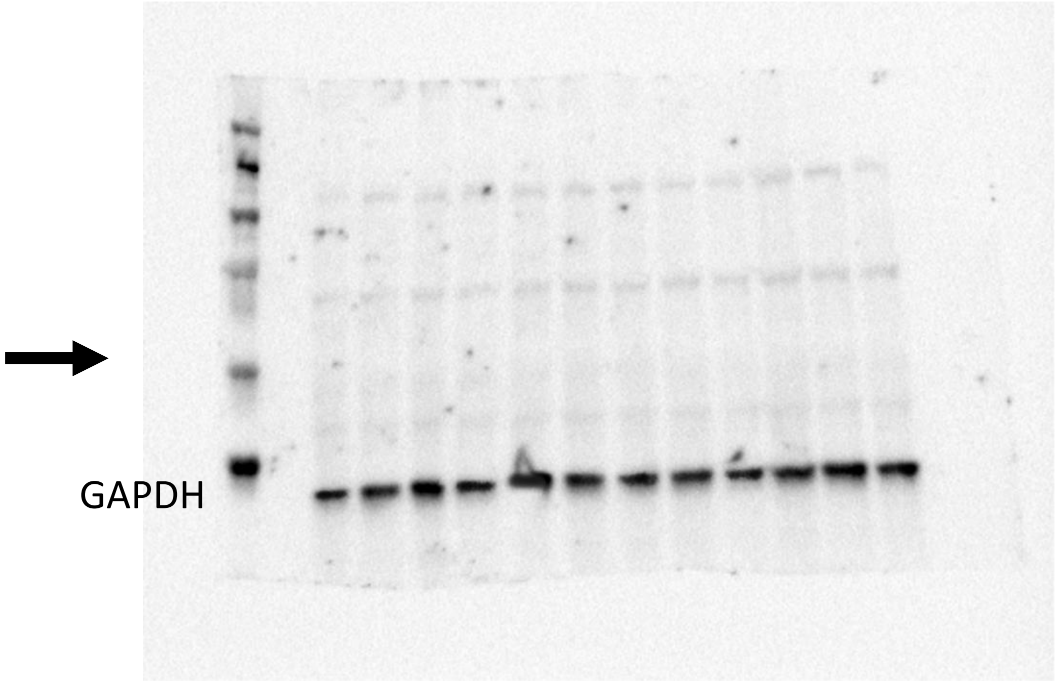

Full unedited blots for SUPPLEMENTARY FIGURE 25C

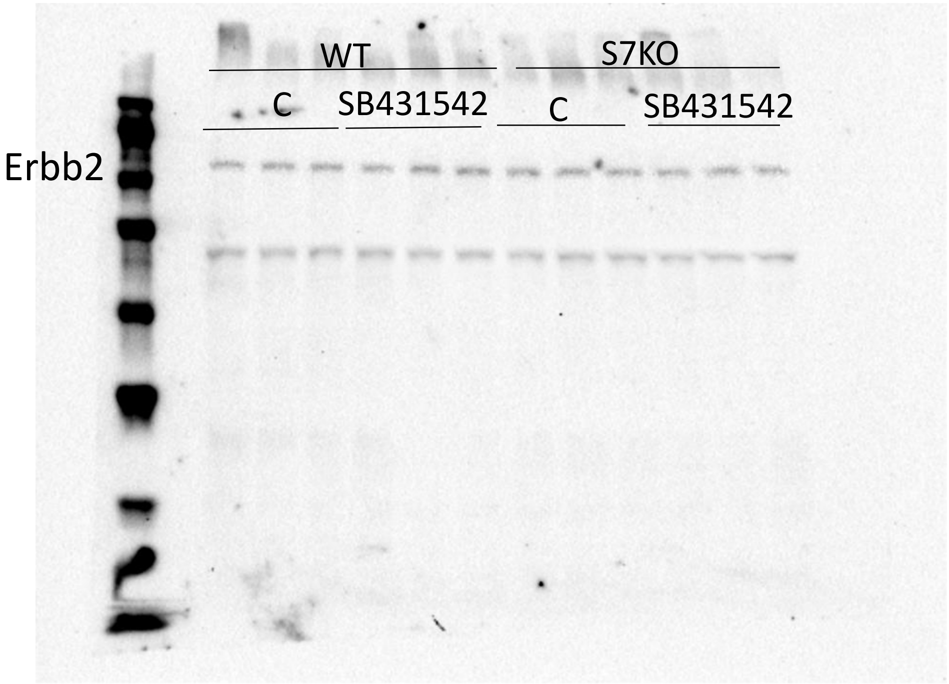

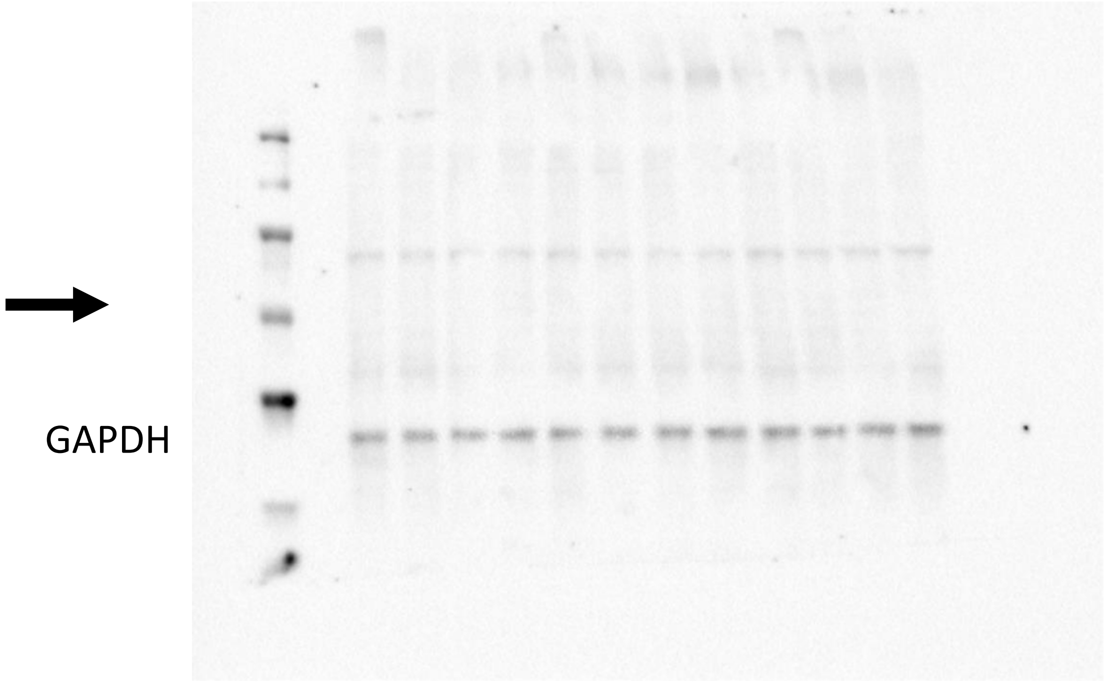

Full unedited blots for SUPPLEMENTARY FIGURE 25F

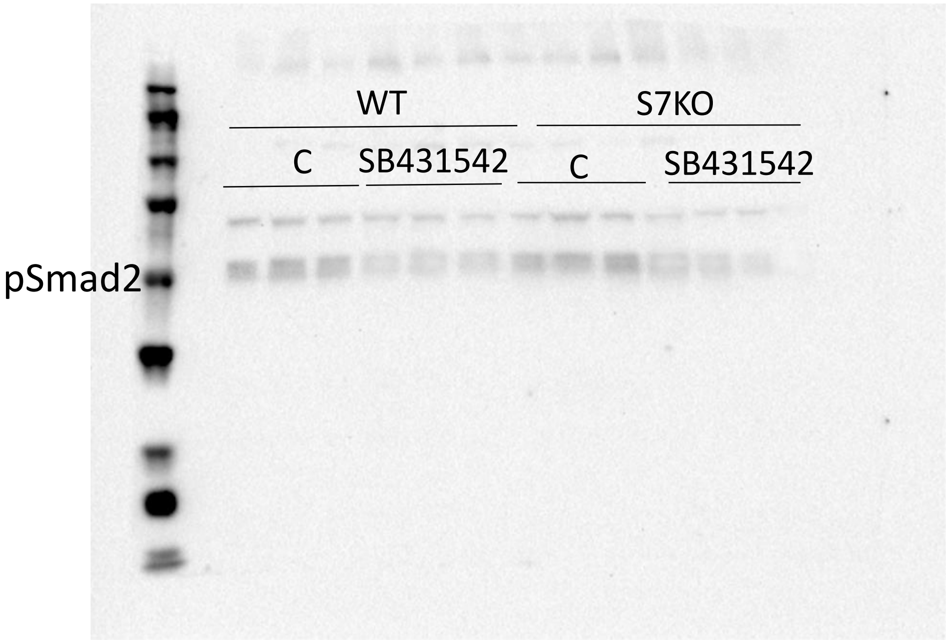

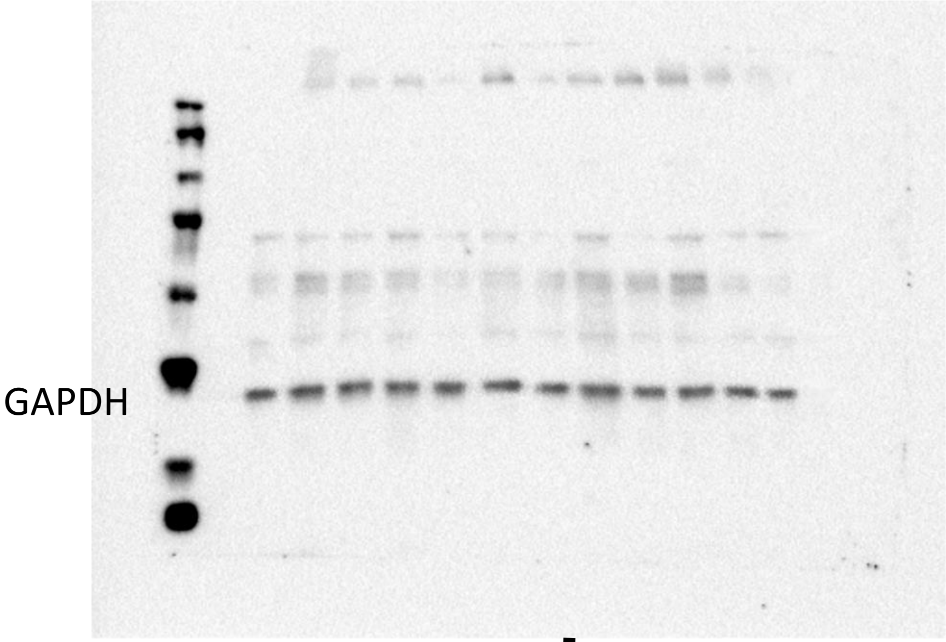

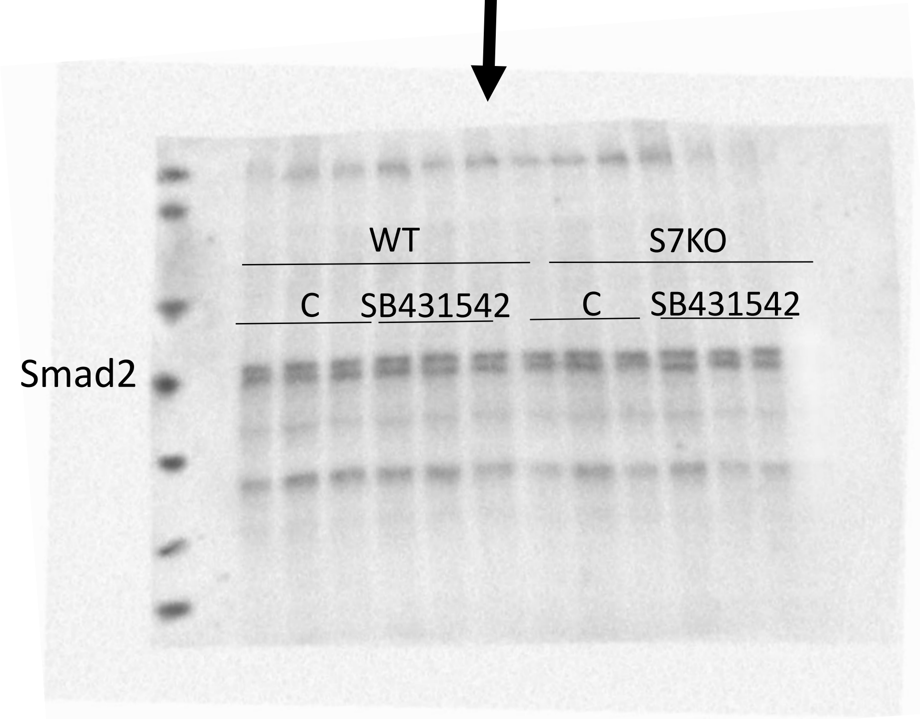

Full unedited blots for SUPPLEMENTARY FIGURE 26A

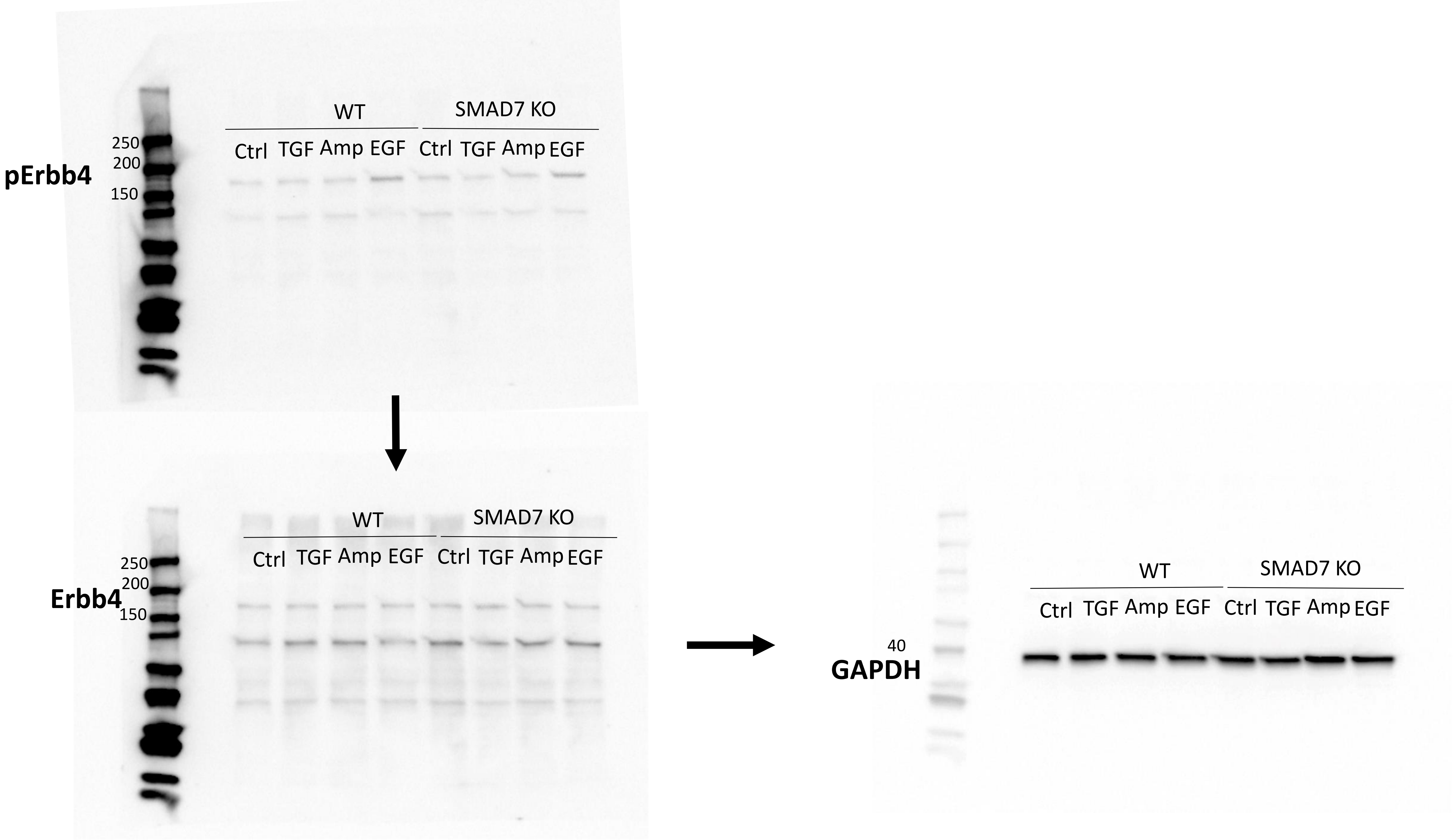

Full unedited blots for SUPPLEMENTARY FIGURE 26E

pErbb4

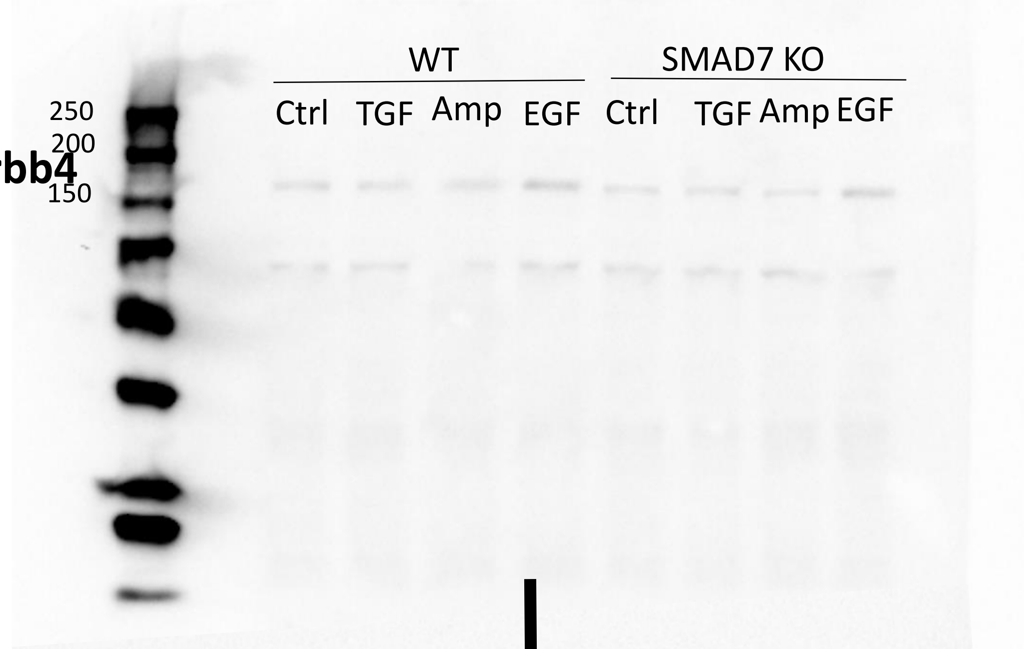

Erbb4

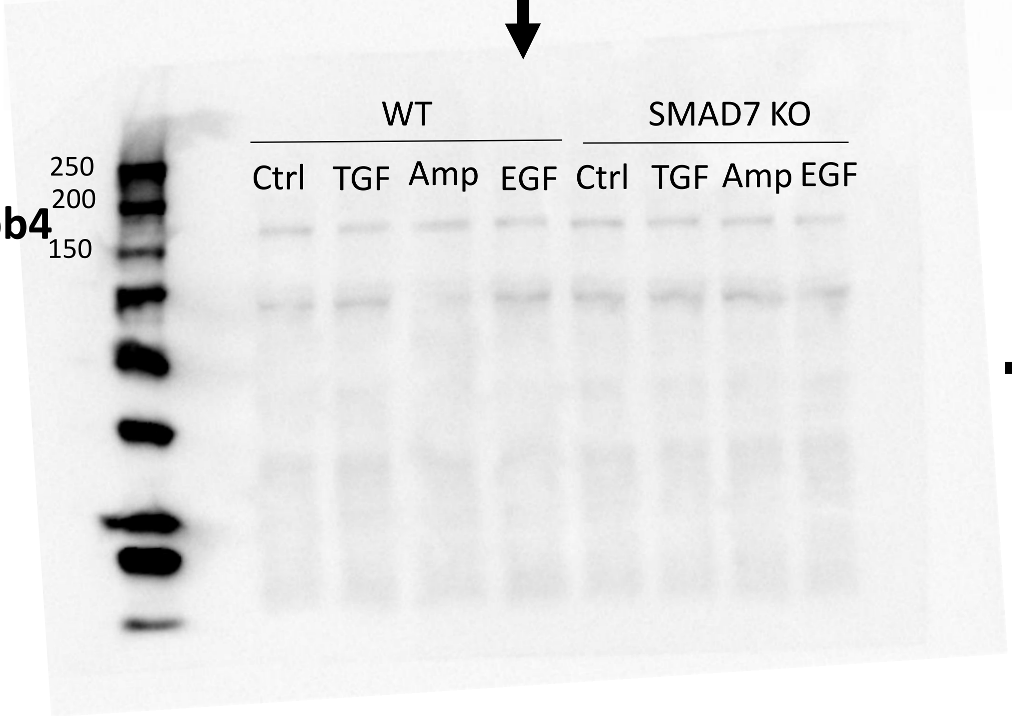

GAPDH

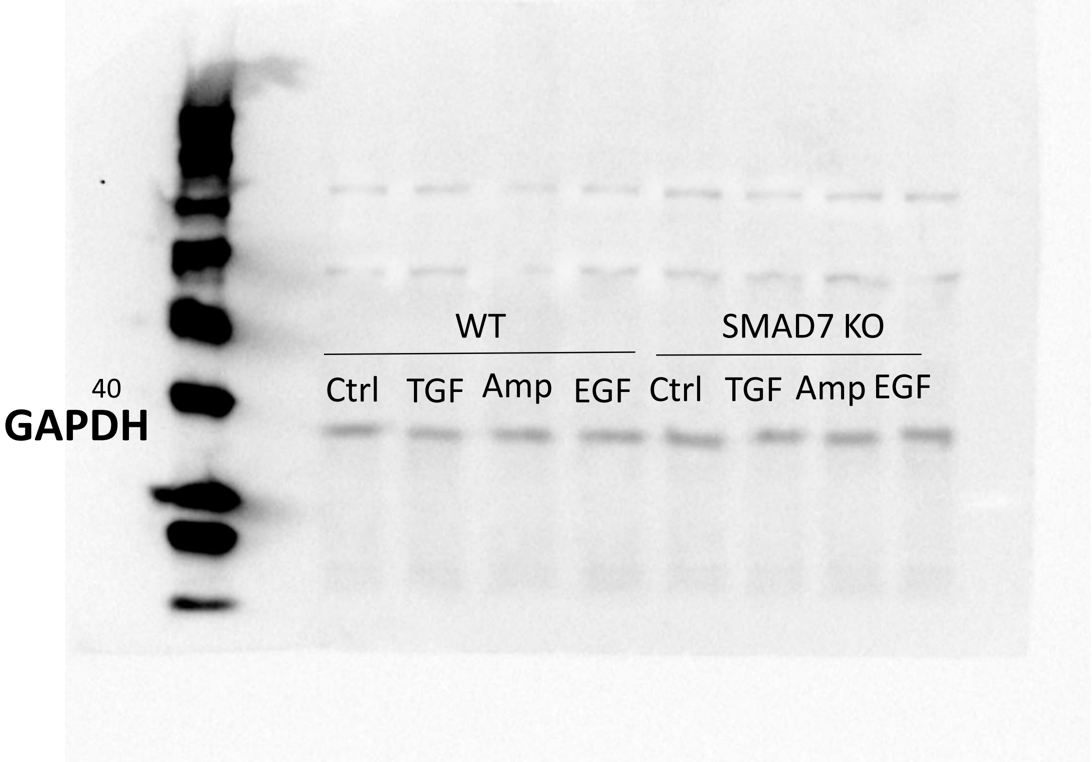

Supplement: Supplemental data [file jci-132-146926-s054.pdf]
